# On‐Resin Recycling of Acid‐Labile Linker Enables the Reuse of Solid Support for Fmoc‐Based Solid Phase Synthesis

**DOI:** 10.1002/marc.202500073

**Published:** 2025-03-08

**Authors:** Nicholas Jäck, Laura Hartmann

**Affiliations:** ^1^ Department for Macromolecular Chemistry University Freiburg Stefan‐Meier‐Straße 32 79104 Freiburg i.Br. Germany

**Keywords:** cyclic acetal, Fmoc peptide chemistry, recycling, reusability, solid phase synthesis

## Abstract

In this study, the first recyclable and reusable polystyrene solid support (resin with functional linker) for Fmoc‐based solid phase synthesis (SPS) for the synthesis of sequence‐defined oligoamides and peptides is presented. By introducing an acid‐labile cyclic acetal linker, efficient oligomer cleavage under mildly acidic conditions comparable to conventional linkers is achieve while also enabling efficient on‐resin regeneration of the linker. This regeneration ability allows the support to be reused for multiple synthesis cycles without compromising the flexibility, high reproducibility, and structural control inherent to solid‐phase synthesis. As a proof of concept, the robustness of this approach is demonstrated by synthesizing different dimeric structures in an alternating manner on the same resin. For each cycle, the oligomer is first elongated through building block coupling, followed by cleavage from the solid support to release the product. The linker is then regenerated on the functionalized solid support, allowing the cycle to be repeated for the synthesis of subsequent oligomers. This approach maintains high yields and purity across multiple cycles, illustrating the potential as a versatile and more sustainable methodology for Fmoc‐based solid phase synthesis.

## Graphical abstract

This study introduces the first recyclable and reusable resin for Fmoc‐based solid phase synthesis (SPS) of oligamide‐based structures through the regeneration of a cyclic acetal‐based linker on Tentagel resins. Even after multiple recycling and reuse cycles of the solid phase, consistently high yields and high‐purity products are obtained.

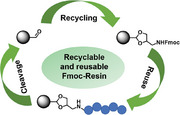

## 1 Introduction

Since its introduction by Merrifield in 1963, solid phase synthesis (SPS) has become a highly versatile method for chemically accessing biomacromolecules such as peptides, oligonucleotides, and oligosaccharides.^[^ ^1^, ^2^ ^]^ Merrifield originally introduced SPS as an innovative technique for efficiently assembling peptides on a solid support. This technique enabled the synthesis of complex peptides with high sequence control and low dispersity, omitting time‐ and material‐consuming chromatographic purification steps during the synthesis.^[^ ^3^ ^]^ Modern SPS typically employs a polystyrene (PS)‐based resin with a cleavable, mostly acid‐labile linker and terminally protected amino groups.^[^ ^4^ ^]^ Amino acids and other carboxylic acid‐functionalized building blocks can be covalently linked to the solid support via an amide linkage, allowing for the stepwise deprotection of the amine and coupling of the next building block to create the desired macromolecule.^[^ ^4^ ^]^ Overall, this method enables the synthesis of various custom‐designed biomacromolecules with high‐purity and efficiency, which is crucial for various areas of research and application, including pharmaceuticals, biotechnology, and material sciences.^[^ ^5^ ^]^ Today, SPS is also used to synthesize complex organic compounds and sequence‐defined non‐natural macromolecules or polymers.^[^ ^6^, ^7^ ^]^ Inspired by peptides, various types of peptidomimetics have been developed on solid support.^[^ ^8^ ^]^ For example, Hartmann et al. demonstrated the use of SPS to gain access to monodisperse oligoamidoamines by the stepwise assembly of diacid and diamine building blocks for use as non‐viral vectors in gene therapy.^[^ ^9^ ^]^ Recently, the preparation of sequence‐defined “digital” polymers on solid supports to encode data within the polymer chains for molecular data storage applications has gained increasing interest.^[^ ^10^ ^]^ Today, various types of non‐natural building blocks are assembled on solid support to derive a large variety of synthetic and biomacromolecules or conjugates thereof.^[^ ^11^ ^]^ In our group, we derive various sequence‐defined glycooligomers as bacterial and viral inhibitors or as mimetics of native cell surface glycans, demonstrating the advantage of SPS to achieve sequence control even in complex biomimetic structures.^[^ ^12^ ^]^

SPS provides several advantages over the synthesis in solution. These include ease of purification through simple washing steps, compatibility with automation and scalability, and the ability to use excess reagents to drive reactions to completion.^[^ ^13^ ^]^ Furthermore, the method minimizes the handling of intermediates and offers superior control over sequential synthesis.^[^ ^8^, ^14^ ^]^ These attributes make SPS particularly well‐suited for the synthesis of complex macromolecules and for applications in combinatorial chemistry, where large libraries of compounds can be synthesized efficiently.^[^ ^15^ ^]^ Despite their advantages in precision and automation, traditional SPS protocols generate significant waste due to the solid support's single‐use nature, high amounts of hazardous solvent, and the requirement for high equivalents of building blocks to ensure complete reactions.^[^ ^16^, ^17^ ^]^

In the quest for more sustainable synthetic processes, recent advances have focused on reducing waste and improving the efficiency of SPS methodologies. For instance, advancements have been made in reagent recycling, solvent reduction, and adopting more environmentally friendly solvents.^[^ ^16^, ^17^, ^18^ ^]^ Our group has previously demonstrated the potential for recycling and reusing building blocks in SPS. We introduced a method for the recovery, purification, and reuse of Fmoc‐protected building blocks and functionalized carbohydrate ligands.^[^ ^16^ ^]^ This process, which relies on simple techniques like precipitation and recrystallization, can be integrated into automated peptide synthesizers and adapted for various chemically distinct building blocks. Pon et al. introduced a general method for oligonucleotide synthesis on reusable solid supports. Their system employed a hydroquinone‐O,O'‐diacetic acid (Q‐linker) for nucleoside attachment, which enabled efficient cleavage and surface regeneration, allowing the solid support to be reused for multiple synthesis cycles without compromising product quality.^[^ ^19^ ^]^ However, to the best of our knowledge, there has been no attempt to functionalize a resin with a recyclable linker for the repeated reuse of solid support in multiple synthesis and cleavage cycles under standard Fmoc peptide chemistry protocols, e.g., for the synthesis of amide‐based oligomers.

**Figure 1:**
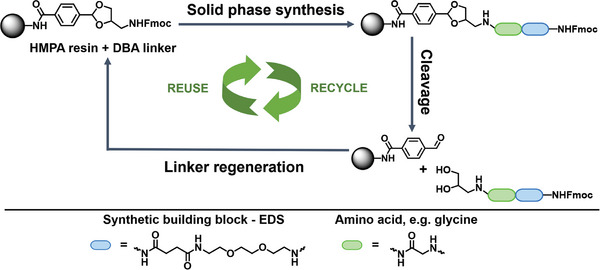
Schematic representation of a recyclable and reusable resin for Fmoc‐based SPS using the cyclic acetal‐based DBA linker.

Our concept is based on an acid‐labile building block suitable for Fmoc‐based solid phase synthesis and termed DBA (4‐(4‐(((((9H‐fluoren‐9‐yl)methoxy)carbonyl)amino)methyl)‐1,3‐Dioxolan‐2‐yl)Benzoic Acid), that we recently introduced.^[^ ^20^ ^]^ This building block features a cyclic acetal group as the cleavable moiety with a terminal Fmoc‐protected amine and a free carboxylic acid for chain elongation. In this work, we aim to use DBA as a cleavable linker replacing conventional linker systems used in SPS such as trityl linkers or Rink amide linkers.^[^ ^4^ ^]^ Upon acid‐catalyzed hydrolysis, DBA is converted into an aldehyde‐functionalized solid support, which can then be utilized to regenerate the cleavable acetal linker. This allows for repetitive on‐resin building block (e.g., amino acids or tailor‐made building blocks) coupling, oligomer cleavage, and linker regeneration, enabling recycling of the solid support and reuse for multiple independent SPS syntheses (**Figure** **1**).

## 2 Results and Discussion

### 2.1 A Cyclic Acetal‐Based Cleavable Linker for Solid Phase Synthesis

**Figure 2:**
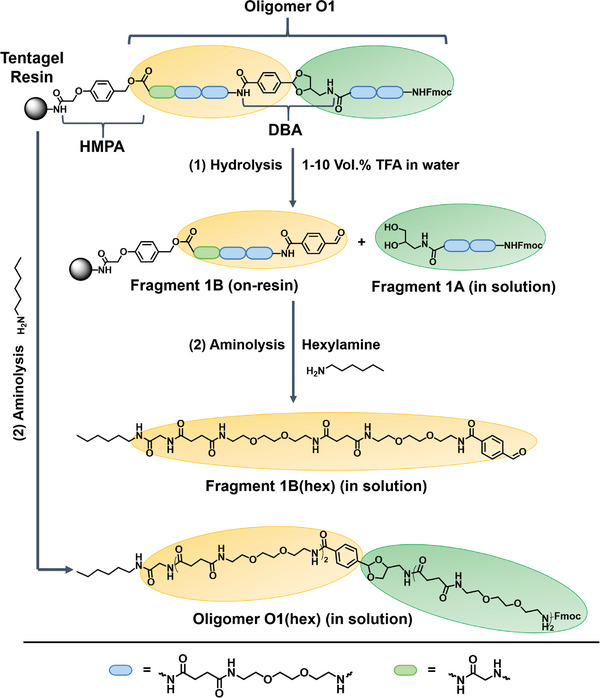
Cleavage pathways for Oligomer O1 were utilized as a model structure to determine the on‐resin DBA cleavage efficiency. 1) Hydrolysis pathway: Oligomer O1 is treated with varying concentrations of TFA (1‐10 vol.%) in water, selectively cleaving fragment 1A from the solid support while leaving fragment 1B resin‐bound. After hydrolysis, 1B undergoes subsequent aminolysis to determine the conversion rate of O1 into 1B. 2) Aminolysis pathway: Oligomer O1 is fully cleaved from the resin via aminolysis, releasing the entire oligomer into the solution for structural analysis of O1(hex).

As our goal was to develop a recyclable solid support capable of withstanding multiple synthesis and cleavage cycles under standard Fmoc‐based SPS conditions, we began by evaluating the suitability of DBA as a cleavable linker for SPS and optimizing its on‐resin cleavage conditions. Therefore, a DBA‐containing oligomeric structure was synthesized using standard Fmoc‐based SPS protocols. The Tentagel R HMPA (4‐Hydroxymethyl‐phenoxy acetic acid) resin was selected to allow for the sequential cleavage of the orthogonal linkers DBA and HMPA without cross‐interference. Although the HMPA linker is commonly cleaved under strongly acidic conditions with TFA, we have previously shown that this linker can also be cleaved via aminolysis, while DBA is cleaved under mildly acidic conditions thus enabling orthogonal cleaving conditions.^[^ ^20^ ^]^ This resin was primarily used for reaction analysis and optimization before being transferred to the Tentagel S Amine resin, which lacks an inherent linker. The results regarding transfer to this resin will be described later on in this manuscript. This combination enables the assessment of the on‐resin DBA linker cleavage efficiency by first cleaving DBA under mildly acidic conditions and releasing fragment A (**Figure** **2**) from the resin. After subsequential aminolysis, the previously still attached fragment B (Figure 2) will now also be cleaved off the resin and can be analyzed to determine the hydrolysis rate of DBA via RP‐HPLC‐MS (reversed phase – high‐performance liquid chromatography – mass spectrometry) analysis (see Supporting Information).

The oligomer used for this purpose was assembled through standard SPS protocols utilizing Fmoc‐protected building blocks 1‐(9H‐fluoren‐9‐yl)‐3,14‐dioxo‐2,7,10‐trioxa‐4,13‐diazaheptade‐can‐17‐oic acid (EDS) and DBA with *N,N*‐dimethylformamide (DMF) as the solvent and (Benzotriazol‐1‐yloxy)tris‐(pyrrolidino)phosphonium hexafluorophosphate (PyBOP) and N,N‐Diisopropyl‐ethylamine (DIPEA) as coupling agent and base. Following each coupling step, the Fmoc group was removed using 20% piperidine in DMF, allowing the next building block to be attached (see SI for further details on the synthesis). This cycle was repeated until the desired sequence of oligomer O1 was achieved (see Figure 2). During hydrolysis, DBA is cleaved, releasing the diol‐terminated fragment 1A (Figure 2), while the aldehyde‐terminated fragment 1B (Figure 2) remains bound to the resin. To enable efficient RP‐HPLC‐MS analysis after subsequent aminolysis, EDS was chosen as a building block to increase the molecular weight of the resin‐bound fragment 1B (Figure 2). This aldehyde‐terminated fragment is crucial for recycling the cleavable cyclic acetal linker, which initiates the reusability of the solid support, a process that will be discussed in detail later.

To determine the on‐resin cleavage efficiency of DBA, various conditions were tested to identify the most efficient protocol based on their varying strengths in promoting acid‐catalyzed hydrolysis. As demonstrated in our previous study, DBA can be quantitatively hydrolyzed in solution within 45 min with a 1 vol.% TFA solution in water. However, we also found that the stability of DBA is enhanced through the proximity of several DBA units (as in our previously investigated DBA containing micelles), suggesting that the hydrolysis rate of DBA on resin could also be slower than in solution.^[^ ^20^ ^]^

Consequently, we treated resin‐bound O1 with different concentrations of TFA in water (1, 3, 5, and 10 vol.%) for 30 min. After thoroughly washing the resin to remove residual TFA and fragment 1A, a portion of the resin was subjected to aminolysis for on‐resin DBA cleavage analysis via RP‐HPLC‐MS analysis (**Table** **1**). The remaining resin was incubated again under the same conditions for a second cycle, followed by aminolysis and RP‐HPLC‐MS analysis.

**Table 1:** Determination of the on‐resin cleavage rate of DBA with various TFA solutions after either 30 or 2 × 30 min.

	t = 30 min				t = 2 × 30 min			
TFA (Vol.%)	1	3	5	10	1	3	5	10
cleavage (%)^a)^	89	95	96	97	97	100	100	100

^a)^ Cleavage rate determined via RP‐HPLC‐MS.

The results confirmed that complete cleavage of DBA was achieved after 1 h for all cleavage solutions above 1 vol.% TFA. In solution, full cleavage was already observed after 45 min with 1 vol.% TFA, suggesting that DBA is slightly more stable when bound to the resin. This increased stability may also be attributed to limited accessibility, as DBA is incorporated within the resin matrix, restricting reagent diffusion compared to the solution phase. Additionally, diffusion limitations within the PEG‐polystyrene resin, potentially influenced by solvent volume, could also contribute to the observed cleavage behavior. Based on these findings, we aimed to select the mildest cleavage conditions that quantitatively cleave DBA on resin. Thus, a 3 vol.% TFA solution with two 30 min cleavage cycles was chosen as the standard condition.

### 2.2 On‐Resin Regeneration of Cyclic Acetals

Next, after successful cleavage, the regeneration of the DBA linker was investigated as a critical step in the overall recycling and reusing process. After acidic hydrolysis of O1, resin‐bound fragment 1B presents a terminal aldehyde group (See Supporting Information for ^1^H‐NMR, ESI‐MS, and HPLC). This enables us to adapt the reaction strategy for the in‐solution synthesis of cyclic acetals, and, to the best of our knowledge, shows this type of reaction on solid support via SPS for the first time. Typically, the in‐solution synthesis of cyclic acetals like DBA involves two key steps: the activation of the aldehyde as a dimethylacetal, followed by its cyclization with a diol component.^[^ ^21^ ^]^ Both reactions are usually carried out at high‐temperatures in the presence of an acid catalyst, with excess water being removed using a Dean–Stark apparatus.^[^ ^20^, ^22^ ^]^ However, these conditions are not feasible for solid phase synthesis.

#### 2.2.1 Dimethylacetal Activation of an Aldehyde‐Functionalized Solid Support (Step 1)

**Figure 3:**
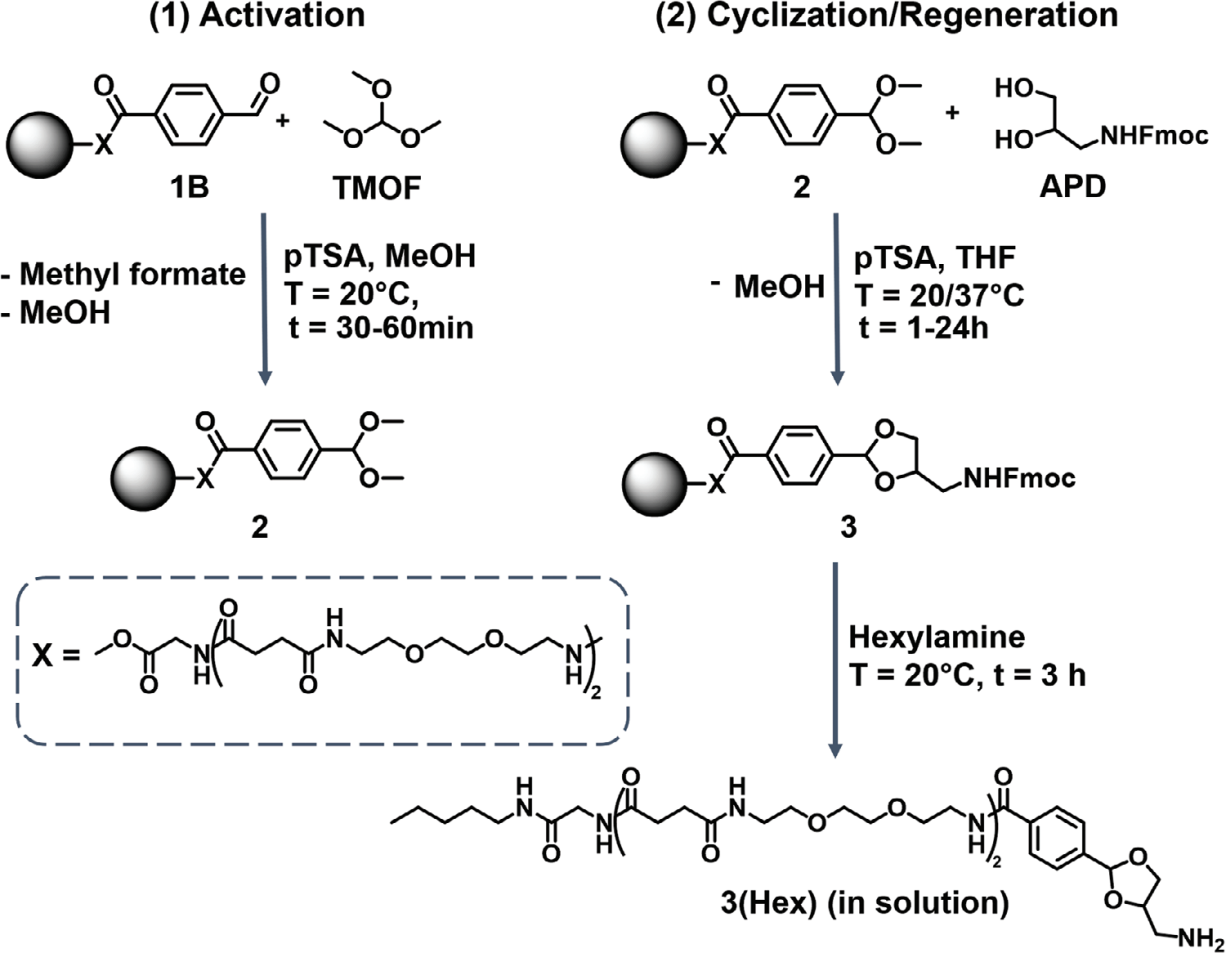
On‐resin linker regeneration pathway of cyclic acetal containing DBA.

To enable the synthesis of the cyclic acetals on the solid support and thus the regeneration of the DBA linker, we first investigated the optimal conditions for the conversion of the resin‐bound aldehyde fragment 1B into the dimethylacetal containing and resin‐bound structure 2 (**Figure** **3**) by varying the amount of the activation reagent trimethyl orthoformate (TMOF) and catalyst p‐toluenesulfonic acid (pTSA) in methanol. The conversion rate was determined via RP‐HPLC‐MS analysis (See Supporting Information).

All reaction conditions (See Supporting Information for experimental details) yielded ≈80% of the resin‐bound and activated structure 2, with the remaining 20% corresponding to the unreacted aldehyde‐functionalized and resin‐bound fragment 1B. Interestingly, samples activated in two 30 min cycles with either 0.01 eq. or 0.03 eq. pTSA exhibited a slightly higher conversion rate of ≈85% (see Supporting Information). This yield is slightly lower than the 95% efficiency achieved during DBA synthesis in solution, however, to demonstrate the feasibility of linker regeneration in this study, we refrained from further optimization at this time.^[^ ^20^ ^]^ Consequently, all subsequent activation steps for 1B (0.025 mmol) employed two 30 min cycles with 1 mol% pTSA in 1.5 mL of dry TMOF and 0.5 mL of dry methanol as optimized conditions.

#### 2.2.2 Solid Phase Synthesis of Cyclic Acetals (Step 2)

As the next step in regenerating the linker, the cyclization of activated 2 with the diol‐containing fragment (9H‐fluoren‐9‐yl)methyl (2,3‐dihydroxypropyl)carbamate, termed APD, into cyclic acetal containing 3 (Figure 3) was studied. APD was chosen as the diol‐containing fragment, as it contains a Fmoc‐protected amine for further chain elongation options (see Supporting Information for APD synthesis).

In the cyclization reaction, we systematically varied different reaction parameters. As the starting protocol, a resin bearing 0.025 mmol of 2 was incubated with 0.01 eq. pTSA and 12.5 eq. of APD in anhydrous THF for 3 h at 37 °C. As the first reaction parameters, reaction time and temperature were varied (1, 3, and 5 h at 37 °C, and 5 and 24 h at 20 °C), followed by varying the amounts of pTSA and APD. The results are summarized in Table 2, following the conversion of activated 2 to cyclic acetal containing 3 and the formation of the aldehyde terminated byproduct 1B by RP‐HPLC‐MS analysis (See Supporting Information for RP‐HPLC‐MS chromatograms).

**Table 2:** Optimization parameters and conversion rates for the on‐resin cyclic acetal formation of resin‐bound 3.

	T = 20 °C^b)^		T = 37 °C^c)^				APD^d)^			pTSA^e)^		
Time (h)	5	24	1	3	5	Equiv.	5	12.5	25	0	0.01	0.02
Yield (%)^a)^	42	70	18	57	69	Yield (%)^a)^	43	57	62	0	57	42

^a)^ determined via RP‐HPLC‐MS;

^b)^ 0.025 mmol of resin‐bound 2 was incubated with 0.01 eq. of pTSA and 12.5 eq. of APD in 2 mL anhydrous THF for 5 h or 24 h at 20 °C;

^c)^ 0.025 mmol of resin‐bound 2 was incubated with 0.01 eq. of pTSA and 12.5 eq. of APD in 2 mL anhydrous THF for 1–5 h at 37 °C;

^d)^ 0.025 mmol of resin‐bound 2 was incubated with 0.01 eq. of pTSA and 5–25 eq. of APD in 2 mL anhydrous THF for 3 h at 37 °C;

^e)^ 0.025 mmol of resin‐bound 2 was incubated with 0‐0.02 eq. of pTSA and 12.5 eq. of APD in 2 mL anhydrous THF for 3 h at 37 °C.

The data indicate that increasing the reaction time enhances product formation, with a complete conversion of 2 achieved after 5 h at 37 °C and 24 h at 20 °C. Before these time points, residual 2 remained partially unreacted on the resin, as confirmed by RP‐HPLC‐MS analysis. At 37 °C, the yield increased from 18% after 1 h to 69% after 5 h, while at room temperature, the yield improved from 42% after 5 h to 70% after 24 h. These results demonstrate that higher temperatures accelerate reaction rates and improve yields. The predominant byproduct in these reactions was the aldehyde‐containing fragment 1B. This suggests that partial hydrolysis of either 2 or 3 occurs during the cyclization step, as the activation step from 1B to 2 preceding the reaction yielded ≈85%, leaving 15% 1B unreacted on the resin.

Reactions using no pTSA did not yield any product, demonstrating the necessity of an acid catalyst for effective cyclization. The highest yield of 57% was achieved with 0.01 eq. In contrast, an excess amount of 0.02 eq. decreases the yield, likely due to the promotion of hydrolysis back to the aldehyde at higher acid concentrations. Consequently, all subsequent experiments used 0.01 eq. of pTSA to ensure efficient cyclization. Increasing the amount of diol building block APD also led to higher yields, as is expected. With 5 eq. of APD, the yield was 43%, whereas using 25 eq. increased the yield to 62%.

Based on our previously established strategy to recycle building blocks, also ≈50% of APD can be recovered from the excess in solution after its use in this reaction by precipitation in water and reused for further cyclization steps, producing comparable conversion rates (See Supporting Information).^[^ ^16^ ^]^

The combined optimized conditions (37 °C for 5 h, 1 mg (0.01 eq.) of pTSA, and 25 eq. of APD) initially resulted in a 67% yield.

Often in SPS approaches, multiple couplings are used to increase yields. Accordingly, a double coupling step was introduced to maximize product yield by reapplying the activation step after cyclization followed by repeating the cyclization with APD, which improved the yield to 76%. For comparison, the double coupling procedure was also applied to the original protocol, which led to a similar yield of 77%. Given the comparable efficiency but with reduced reaction time and lower consumption of APD, the original protocol combined with a double coupling procedure was chosen for subsequent experiments. Specifically, all following on‐resin cyclization reactions were performed at 37 °C for 3 h with 1 mg (0.01 eq.) of pTSA, and 12.5 eq. of APD, utilizing a double coupling procedure.

To evaluate the number of times DBA linker regeneration can be repeated on the Tentagel R HMPA resin, we carried out three consecutive cycles of linker cleavage (1B formation), aldehyde activation (2 formation), and regeneration (3 formation). For each regeneration cycle, reaction conditions as previously established were applied, and the reaction was followed by RP‐HPLC‐MS analysis (See Supporting Information). Product yield remained consistent across all three cycles, ranging from 57% in the first cycle to 55% in the second, and 59% in the third cycle. Thus, the linker and, accordingly, the linker‐functionalized resin can be effectively regenerated multiple times. We further demonstrated the recycling ability of this resin by successfully deprotecting the regenerated terminal amine and successfully coupling one EDS moiety to the resin (see Supporting Information for RP‐HPLC‐MS).

### 2.3 From Regeneration to Recycling: Combination of DBA with the Linker‐Free Tentagel S Amine Resin

**Figure 4:**
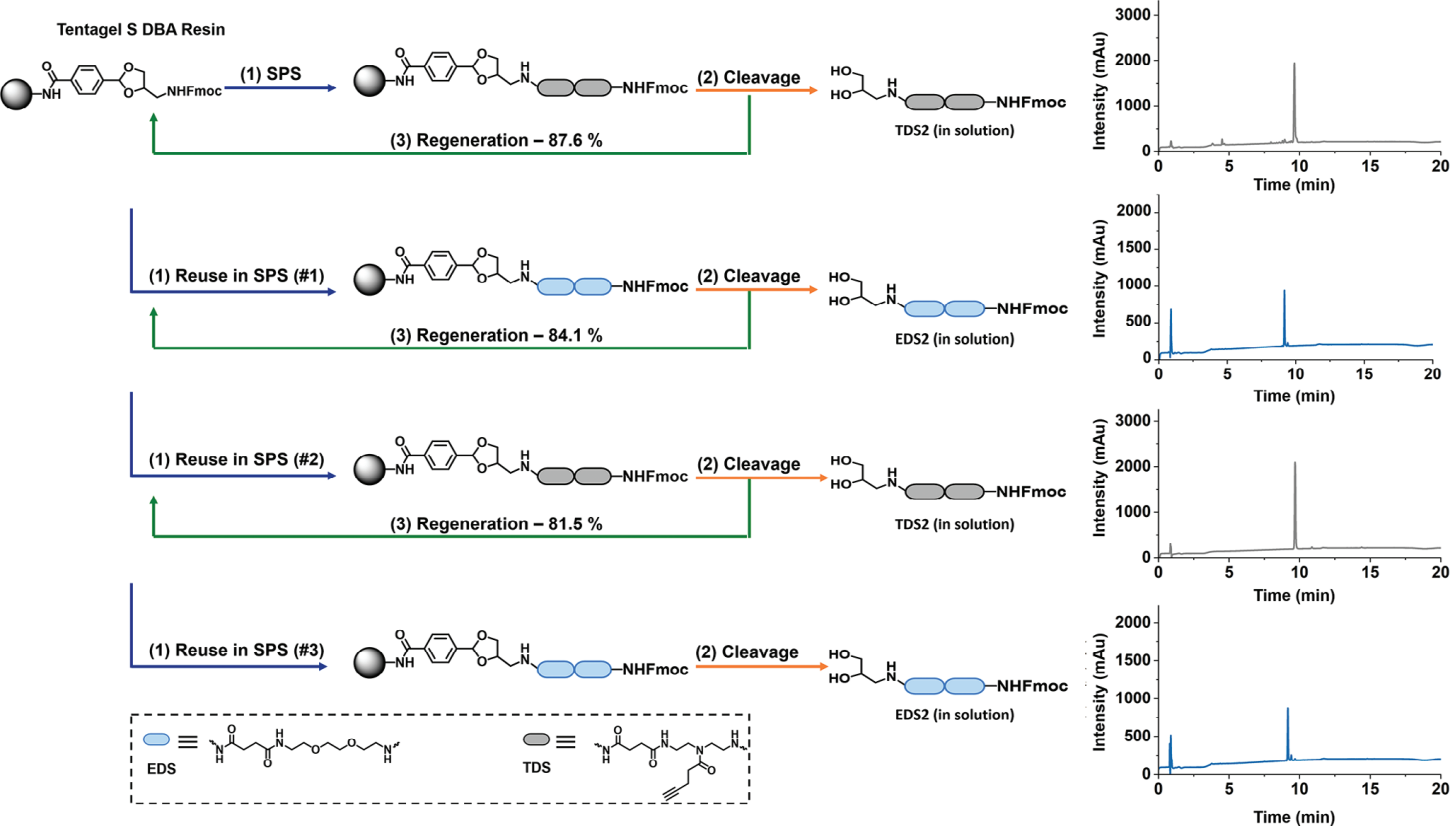
Schematic representation of resin recycling using the Tentagel S DBA resin as the solid support. Each cycle consists of three steps: 1) elongation/reuse via SPS, 2) linker cleavage, and 3) linker regeneration. Two alternating sequences, TDS2 and EDS2, were synthesized in each cycle, demonstrating the reusability of the system. The cleaved products were analyzed by RP‐HPLC‐MS (right diagrams, gradient: 95/5–5/95 vol.% water/acetonitrile with 0.1% formic acid over 20 min at 25 °C) and validated via ¹H‐NMR (see Supporting Information), confirming successful coupling and cleavage.

The optimal conditions for linker regeneration, established using the Tentagel R HMPA resin, were then transferred to the Tentagel S Amine resin. The initial HMPA resin was utilized for analytical purposes to identify both cleaved and resin‐bound fragments of the linker during cleavage and regeneration. For standard SPS procedures, only one linker – now the recyclable DBA linker – is sufficient. Therefore, this linker was coupled onto a Tentagel S Amine resin. To further test this new resin, termed Tentagel S DBA Resin (**Figure** **4**), a first dimeric structure was synthesized consisting of two 1‐(9H‐fluoren‐9‐yl)‐3,11‐dioxo‐7‐(pent‐4‐ynoyl)‐2‐oxa‐4,7,10‐triazatetradecan‐14‐oic acid (TDS) building blocks. This dimer, termed TDS2 (Figure 4), was cleaved and isolated for analysis, and in parallel, the linker was regenerated and the resin was recycled for the synthesis of a second dimer, now consisting of two EDS building blocks. The second dimer (EDS2) was cleaved and isolated for analysis, and the resin was recycled for a second, and then a third time to resynthesize TDS2 and EDS2, respectively (Figure 4).

Following each cleavage cycle, TDS2 and EDS2 were analyzed via RP‐HPLC‐MS (Figure 4) and ¹H‐NMR (see Supporting Information for details) to verify the products and determine their relative purities. All four dimers were obtained with high‐purity, comparable to their synthesis on standard solid supports, confirming the suitability of the DBA resin for the synthesis of sequence‐defined peptides and oligoamides. Additionally, the RP‐HPLC chromatograms in Figure 4 show the absence of alternating structures, confirming that EDS2 was fully cleaved from the solid support, as indicated by the absence of its signal in the chromatogram of TDS2, and vice versa.

The efficacy of DBA regeneration on the Tentagel S Amine DBA resin was quantitatively assessed by comparing the yields of the isolated dimers after each cleavage cycle with the initial yield obtained from the first dimer cleaved before regeneration (TDS2). We observed a slight decrease in yields, with 87.6% after the first cycle (EDS2), 84.1% after the second cycle (TDS2), and 81.5% after the third cycle (EDS2). However, we mainly attribute this to resin loss during handling, as the resin was transferred multiple times between glass vials and polystyrene syringes (see Supporting Information for experimental details). Since the desired product can only form when DBA is effectively regenerated on the resin, the yield directly reflects the amount of DBA successfully regenerated.

## 3 Conclusion

This study introduces the first recyclable and reusable resin for Fmoc‐based solid phase synthesis (SPS), leveraging the cyclic acetal‐based cleavable linker dioxolane benzoic acid (DBA). DBA cleavage and regeneration were systematically optimized on an HMPA resin, achieving consistent yields across multiple synthesis cycles. Successful adaptation to a standard Tentagel S Amine resin was demonstrated, and multiple cycles of solid support recycling (elongation, product cleavage, regeneration of the linker) were performed, confirming high‐purity and yields again via HPLC‐MS and ^1^H‐NMR analysis. This methodology marks a significant advancement in SPS by offering a new versatile and potentially more sustainable linker system. While this study does not yet provide an economic or ecological comparison between standard non‐recyclable and recyclable solid supports, we demonstrate that recycling and reusing resin and linker is feasible without compromising the flexibility, high reproducibility, or structural control of solid phase synthesis. Future work will focus on further optimizing the linker regeneration process, exploring the synthesis of more complex biomolecules using other coupling chemistries, and investigating the potential for full automation and scale‐up.

## Conflict of Interest

The authors declare no conflict of interest.

## Supporting information



Supporting Information

## Data Availability

The data that support the findings of this study are available in the supplementary material of this article.
