# Supplementary material for: On‐Resin Recycling of Acid‐Labile Linker Enables the Reuse of Solid Support for Fmoc‐Based Solid Phase Synthesis
Source: Macromol Rapid Commun. 2025 Mar 8;46(15):2500073. doi: 10.1002/marc.202500073 (PMC12344479; doi:10.1002/marc.202500073)
Supplement: Supplementary file 1 — Supporting Information [file MARC-46-2500073-s001.pdf]

Macromolecular Rapid Communications

Supporting Information

for *Macromol. Rapid Commun.*, DOI 10.1002/marc.202500073

On-Resin Recycling of Acid-Labile Linker Enables the Reuse of Solid Support for Fmoc-Based Solid Phase Synthesis

*Nicholas Jäck and Laura Hartmann**

Supplementary Information

On-Resin Recycling of Acid-Labile Linker Enables the Reuse of Solid Support for Fmoc-Based Solid Phase Synthesis

Nicholas Jäck[a] and Laura Hartmann*[a]

[a] N. Jäck, Prof. Dr. L. Hartmann
Department for Macromolecular Chemistry
University Freiburg
Stefan-Meier-Straße 32, 79104 Freiburg i.Br., Germany
E-mail: Laura.hartmann@makro.uni-freiburg.de

* Correspondence to Tel: +49 761 203 6270; E-mail: laura.hartmann@makro.uni-freiburg.de

Materials

3-amino-1,2-propandiol (fluorochemicals, 98 %), 4-Carboxybenzaldehyde (BLD, 98 %), 9-Fluorenyl methoxycarbonyl chloride(BLD, 98 %), 4-Pentyonic acid (BLD, 98 %), Benzotriazol-1-yloxytripyrrolidinophosphonium hexafluorophosphate (BLD, 98 %), Citric acid monohydrate (Carl Roth, p.a.), Diethylenetriamine (Sigma-Aldrich, 99%), Diisopropylethylamine (Thermo scientific, 99 %), Ethylenedioxybisethylamine (Sigma-Aldrich, 98%), Ethyl trifluoroacetate (Thermo scientific, 99 %), Hexylamine (Thermo scientific, 99 %), Oxalyl chloride (Sigma-Aldrich, 99 %), p-Toluenesulfonic acid monohydrate (Sigma-Aldrich, 98 %), Piperidine (Fisher scientific, 98 %), Succinic anhydride (Sigma-Aldrich, 99 %), Sulfuric acid (Sigma-Aldrich, 99 %), THF anhydrous (Thermo scientific), Triethylamine (TCI, 98 %), Triethylsilane (Thermo Scientific, 98 %), Trifluoroacetic acid (Thermo Scientific, 99 %), Trimethylorthoformate anhydrous (Thermo Scientific, 99 %), Trityl Chloride (Thermo Scientific, 99 %).

All solvents and reagents used were purchased in the highest purity available and used without further purification.

Fmoc-glycine preloaded TentaGel® R HMPA (loading: 0,2 mmol/g) and TentaGel® S Amine resin (loading: 0,25 mmol/g) were purchased from RAPP Polymere GmbH.

Dialysis was performed via diafiltration in VIVASPIN 20 centrifugal concentrators (MWCO: 10 kDa; PES) from sartorius.

Instruments

Nuclear Magnetic Resonance Spectroscopy (NMR)

^{1}H-NMR and ^{13}C-NMR were taken using either a Bruker Advance III 300 or III 400. Coupling constants were given as J in Hertz (Hz) and chemical shifts as delta (δ) in parts per million (ppm). How multiplicities are expressed: s = singlet, d = doublet, t = triplet, q = quartet, m = multiplet.

Reversed Phase – High Pressure Liquid Chromatography (RP-HPLC)

Agilent Technologies 1260 Infinity II series paired with an Agilent quadrupole mass spectrometer with an Electrospray Ionization (ESI) source operating in the 200–2000 m/z range was used for analytical RP–HPLC studies. Solvents A: 95 % H2O, 5 % ACN, +0.1 % formic acid, and B: 5 % H2O, 95 % ACN, +0.1 % formic acid were used to measure each spectrum. The reversed phase column utilized was an MZ-Aqua Perfect C18 column (3.0x50 mm, 3 µM). The UV signal from a wavelength detector tuned to 214 nm was integrated with Agilent Technologies' OpenLab ChemStation LC/MS software to determine the indicated purities.

Lyophilization

The accomplished structures were lyophilized using a Martin Christ Freeze Dryers GmbH Alpha 1-4 LD plus device. The lyophilization process was carried out at -42°C and 0.1 mbar of pressure.

Ultrapure Water

A Thermo Scientific "Barnstead Micropure ST" was used to purify the ultrapure water. 18.20 MΩ*cm was the conductivity.

Centrifuges

Thermo Scientific's "Heraeus Megafuge 8R Centrifuge" was utilized for all centrifugation steps.

Synthesis and analytical data

Synthesis procedures

1) Building block synthesis

   a) EDS, TDS, and DBA were synthesized based on literature protocols[1],[2],[3]

   b) Building block (9H-fluoren-9-yl)methyl (2,3-dihydroxypropyl)carbamate (APD)

2.6 g (29.2 mmol) of 3-amino-1,2propandiol was solubilized in 30 mL of water before 11 g of Fluorenylmethyloxycarbonyl chloride (43.8 mmol) solubilized in 150 mL of THF was added together with 5 mL (29.2 mmol) of DIPEA and stirred at room temperature overnight. Next, the solvent was fully removed under reduced pressure before 100 mL of cold ethyl acetate was added the the solid and stirred for 10 min. The solvent was removed by vacuum filtration, and washed with ethyl acetate dried under vacuum yielding 7.5 g (23.9 mmol, 82 % yield) of the desired product (APD) with a purity of 98 % according to RP-HPLC-MS analysis (**see Figure S2**).

2) Solid-phase synthesis (SPS)

Glycomacromolecules were created using the Fmoc approach in solid-phase polymer synthesis with the building blocks DBA, triple bond-diethylenetriamine-succinic acid (TDS), and ethylene glycol diamine succinic acid (EDS).

Solid phase choices. Fmoc-glycine preloaded Tentagel R HMPA resin was employed to optimize DBA regeneration using SPS. This resin comprises a linker that can be cleaved with nucleophiles using aminolysis, assuring the stability of the DBA linker. In addition, Tentagel S Amine resin, which lacks a cleavable linker, was combined with DBA to create a reusable SPPS resin system. The techniques presented here can be utilized for a batch size of 0.1 mmol, with the option to scale up or down.

Resin swelling. The swelling process began by introducing resin and 7 mL of DCM to a 20 mL polypropylene syringe reactor with a polyethylene frit. The mixture was shaken for 30 min to allow the resin to swell properly. To eliminate impurities, the resin was carefully washed 15 times with 7 mL of DMF. All following washing procedures were done with 7 mL of solvent.

Fmoc-deprotection and coupling. To effect Fmoc-deprotection, the resin was treated to two 15 min incubation cycles with 7 mL of 25 % piperidine in DMF solution. Three washes were conducted after each deprotection procedure, followed by 15 more DMF washes to eliminate the Fmoc-protecting groups completely. The custom-building blocks (5 eq.) were then coupled to the resin using benzotriazol-1-yloxytripyrrolidinophosphonium hexafluorophosphate (PyBOP) (5 eq.) and diisopropylethylamine (DIPEA) (20 eq.) in 4 mL DMF for 1 h. The resin was extensively washed 15 times with DMF to assure purity, and the deprotection and coupling processes followed the same protocol until the desired backbone structure was attained.

Microcleavage - HMPA linker. A small portion of the resin (approx. 10 mg) was incubated with 0.7 mL of hexylamine for 3 h followed by precipitation in 8 ml of cold diethyl ether, centrifugation for 10min at 4400 RPM and 0 °C, and decantation of the ether phase. After adding 0.6 mL of a 1:1 ultrapure water/acetonitril mixture and degassing the solution with nitrogen for 10 min the cleaved product was subjected to RP-HPLC-MS analysis.

Macrocleavage - DBA linker. The resin was washed five times with 7 mL of methanol and five times with 7 mL of ultrapure water to facilitate preparation for subsequent reactions. Detachment from the resin was then initiated by drawing an 8 mL TFA solution (3 Vol.% in

water) and shaking the syringe for 30 min. After washing the resin with 2ml of ultrapure water and adding the washing solution into the collected cleavage solution. The cleavage and washing solutions were combined, degassed with nitrogen for 30 min to remove excess TFA, and lyophilized to isolate the desired product.

3) Optimization of linker cleavage and regeneration (Tentagel R HMPA Resin)

All reactions were performed utilizing a batch size of 0.025 mmol.

Oligomer synthesis (O1). The following oligomeric sequence was initially assembled onto the resin via SPS as mentioned above - ***EDS-EDS-DBA-EDS-EDS*** and termed **O1**. The structure was then analyzed after microcleavage via RP-HPLC-MS analysis.

DBA cleavage (1B(hex)). The resin was washed five times with 4 mL of methanol and five times with 4 mL of ultrapure water to remove any organic solvent residues on the resin. Cleavage of DBA was then initiated by drawing 2 mL of various TFA solutions (1, 3, 5, and 10 Vol.% in water) into the syringe and shaking for 30 min. After washing the resin with 2 mL of ultrapure water the cleavage was repeated with a fresh cleavage solution. After the second cleavage cycle, the resin was washed five times each with water, methanol, and DCM.

Optimal reaction conditions for this step: Two 30 min cycles with 2 mL of 3 Vol.% TFA in water.

Activation of the aldehyde (2). After quantitative DBA cleavage, the aldehyde-terminated resin was activated by incubating the resin with the following solution for 30-60 min: 1.5 mL Trimethylorthoformiate (TMOF), 0.5 mL methanol and either 1 mol% (1 mg) or 3 mol% (3 mg) of p-toluenesulfonic acid (pTSA). After this step, the resin was washed ten times with methanol and five times with DCM before the microcleavage was performed for RP-HPLC-MS analysis.

Optimal reaction conditions for this step: Two 30-minute cycles with 1 mol% pTSA in 1.5 ml of dry TMOF and 0.5 ml of dry methanol.

Cycling acetal formation (3). After activation, the resin was transferred to a glass vial containing 0-2 mol% (2 mg) of pTSA, 5-25 eq. (50-250 mg) of APD and degassed for 1 min with nitrogen before 1.5 mL of THF was added. The vial was shaken for either 5 h or 24 h

(20 °C) and reactions conducted at 37 °C were incubated for 1-5 h. After the reaction was complete, the resin was transferred back to the polypropylene syringe reactor and washed ten times with methanol and DCM before the microcleavage was performed for RP-HPLC-MS analysis.

To further enhance the yield, a double coupling procedure can be performed by repeating the activation and coupling step (double coupling procedure).

Optimal reaction conditions for this step: 37°C for 3 hours, 1 mg (1 mol%) of pTSA, and 12.5 equivalents of APD, utilizing the double coupling procedure.

Elongation (4). After linker regeneration, Fmoc-deprotection, and coupling with **EDS** were performed as mentioned above before creating the sequence ***EDS-EDS-DBA-EDS*** on solid phase, termed **4**. The structure was analyzed after microcleavage via RP-HPLC-MS analysis.

4) Recycling of Tentagel DBA Resin

The model sequence for this resin comprised of two EDS coupled onto 0.05 mmol of the Tentagel S Amine resin followed by DBA. After this, alternating sequences of either two TDS (oligomer **TDS2**) or two EDS (oligomer **EDS2**) units were coupled onto the resin. First, two TDS units were utilized and cleaved from the resin. The cleavage solutions were combined and lyophilized to obtain **TDS2(1).** Parallel to the isolation of the cleaved product, the regeneration of DBA was performed with the optimal conditions mentioned above for cleavage, activation, and cyclization followed by coupling of two EDS units and subsequent cleavage and isolation (**EDS2(1)**). The process of cleavage, isolation, regeneration, and coupling was repeated first with two TDS units and then with two EDS units, resynthesizing **TDS2(2)** and **EDS2(2)**, respectively. All four isolated products were analyzed via ^{1}H-NMR and RP-HPLC-MS analysis.

5) Recycling of APD

After performing the regeneration step on the solid phase, the APD-containing THF solution was precipitated in excess water. After centrifugation at 4400 RPM for 10 min the liquid phase was decanted. The tube was then filled with water, shaken for 30 s, and centrifugated again at 4400 RPM. After decantation, the process was repeated once before the solid was lyophilized

to obtain recycled APD (APD_Rec) with a yield of 50 % and a purity of 98 % determined via RP-HPLC-MS.

Analytical data

a) *Building block (9H-fluoren-9-yl)methyl (2,3-dihydroxypropyl)carbamate (APD) and recycled building block APD_Rec*

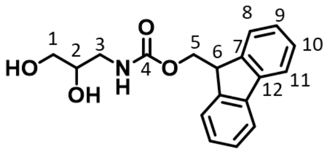

^{1}H NMR (300 MHz, MeOD): δ(ppm) = (d, J = 7.5 Hz, 2H, *H*-9), 7.65 (d, J = 1.3 Hz, 2H, *H*-6), 7.44 – 7.26 (m, 4H, *H*-7, *H*-8), 4.36 (d, J = 7.5 Hz, 2H, *H*-4), 4.20 (t, J = 6.9 Hz, 1H, *H*-5), 3.66 (q, J = 5.6 Hz, 1H, *H*-2), 3.56 – 3.39 (m, 2H, *H*-1), 3.29 – 3.21 (m, 1H, *H*-3), 3.18 – 3.06 (m, 1H, *H*-3).

^{13}C NMR (101 MHz, MeOD): δ(ppm) 159.20(C-4), 145.30(C-7), 142.59(C-12, 128.45 (C-10), 126.14(C-9), 120.91(C-8), 72.24(C-2), 67.78(C-5), 64.99(C-1), 48.45(C-6), 44.63(C-3).

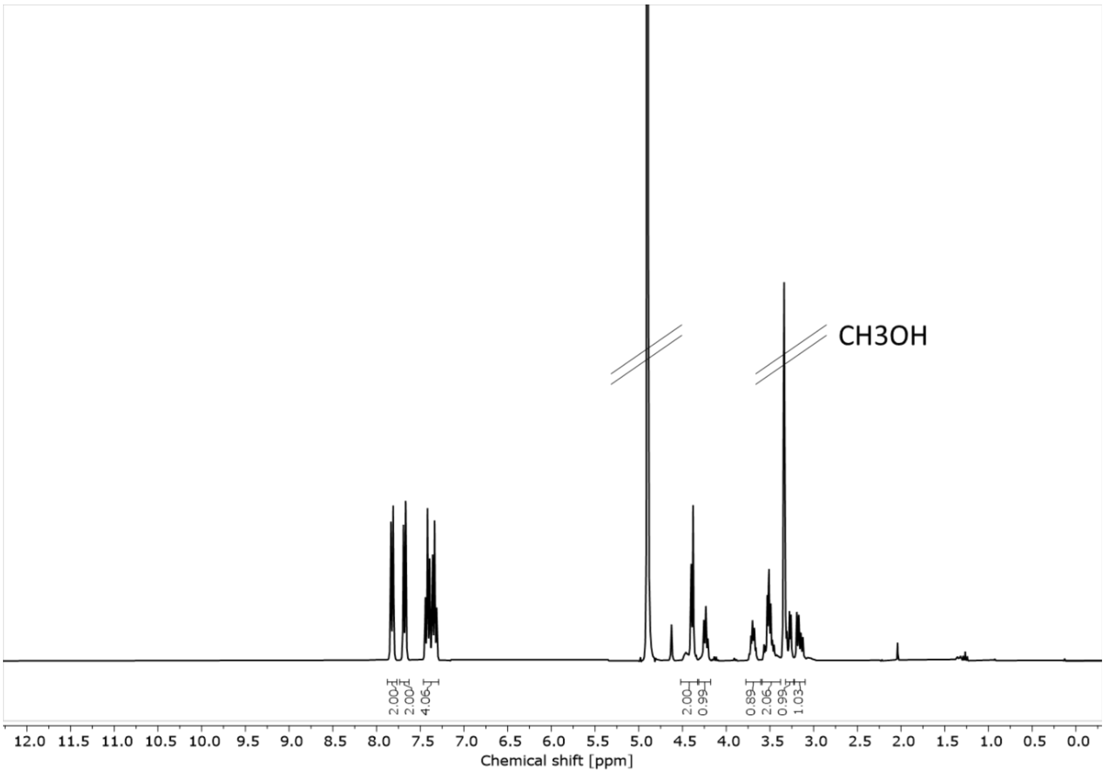

Figure S1 ^{1}H-NMR of APD (300 MHz, CD_3OD, 25 °C).

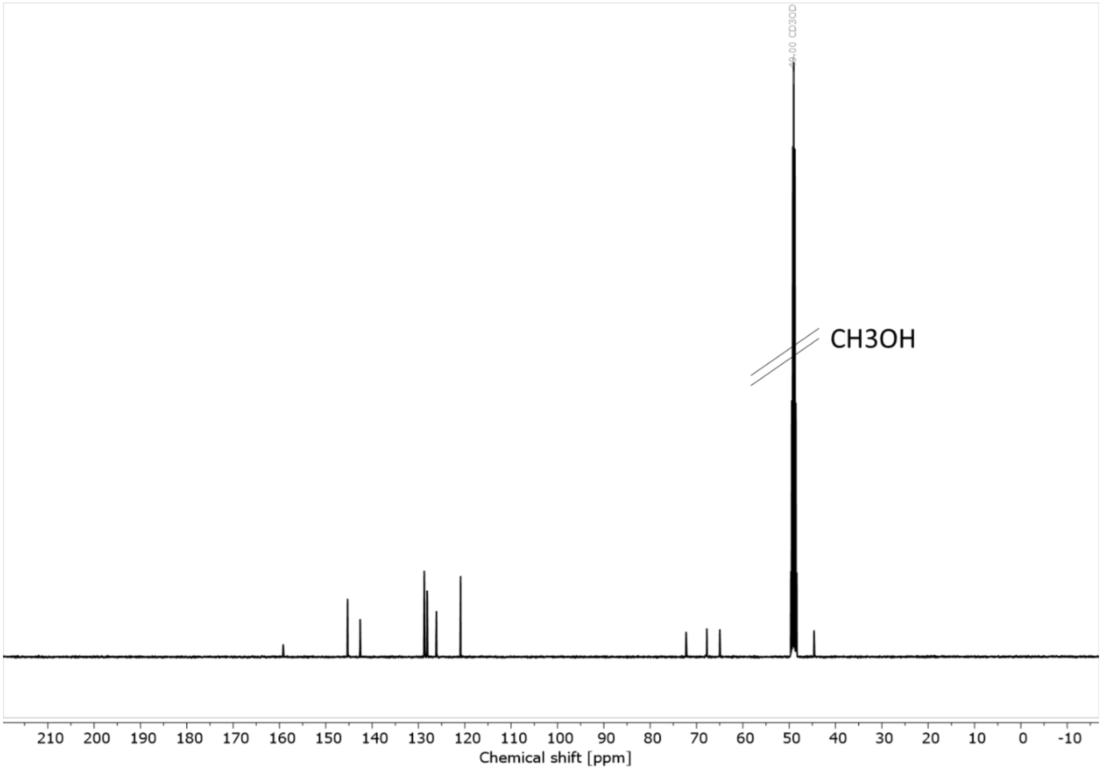

Figure S2 ^{13}C-NMR of APD (300 MHz, CD_3OD, 25 °C).

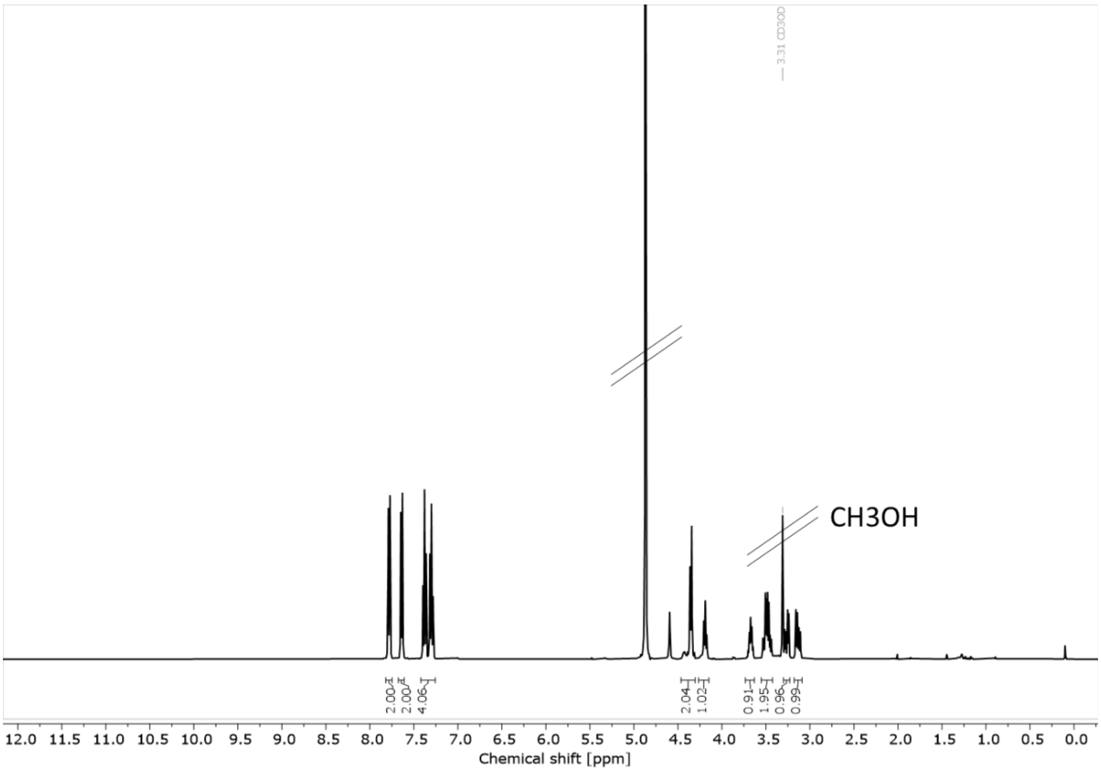

Figure S3 ^{1}H-NMR of APD_Rec (400 MHz, CD3OD, 25 °C).

RP-HPLC: t_R = 9.69 min, 98 % relative purity (UV), from 95/5 to 5/95 Vol.% Water/acetonitrile with 0.1 % formic acid in 20 min at 25 °C.

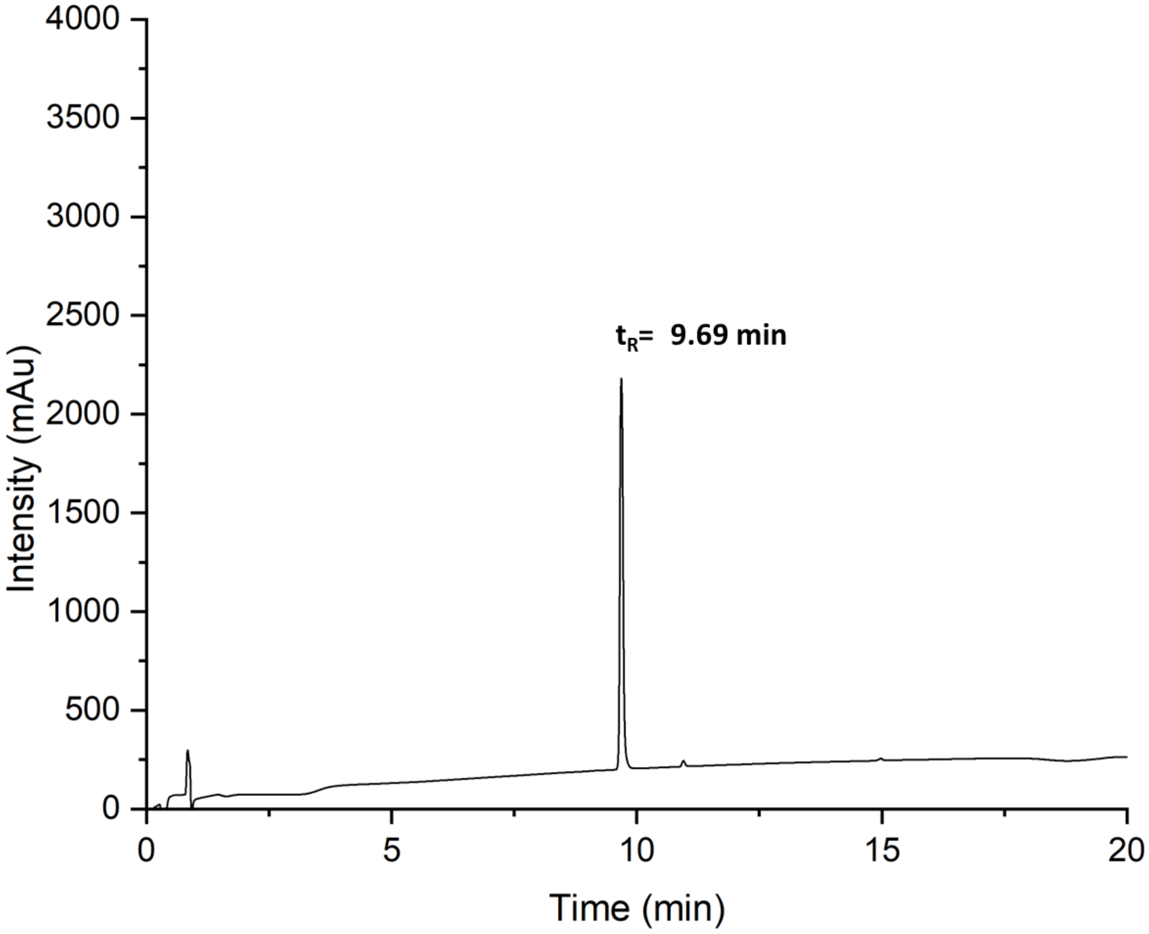

Figure S4 RP-HPLC chromatogram (from 95/5 to 5/95 Vol.% Water/acetonitrile with 0.1 % formic acid in 20 min at 25 °C) of APD**.**

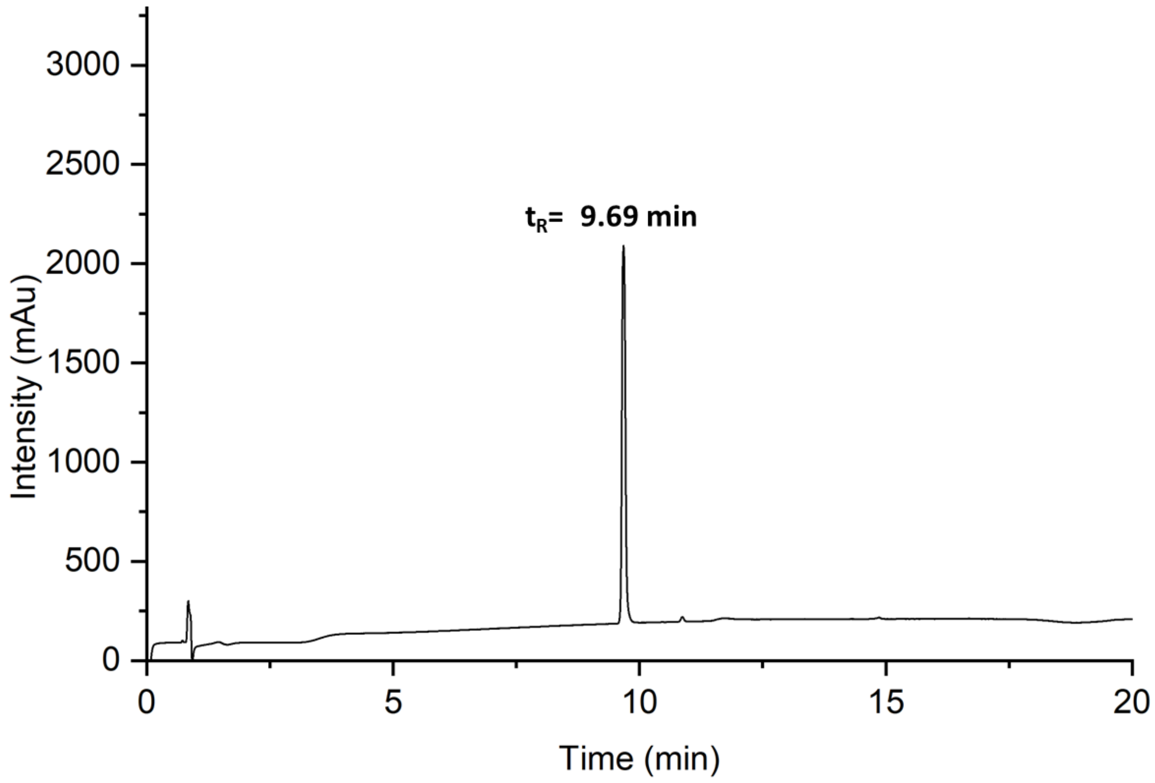

Figure S5 RP-HPLC chromatogram (from 95/5 to 5/95 Vol.% Water/acetonitrile with 0.1 % formic acid in 20 min at 25 °C) of APD_Rec.

ESI-MS calc. for $C_{18}H_{19}NO_4$: $[M+1H]^{2+}$ 314.1, $[M+1Na]^{3+}$ 336.1; found 314.2 $[M+1H]^{+}$, 336.2 $[M+1Na]^{+}$

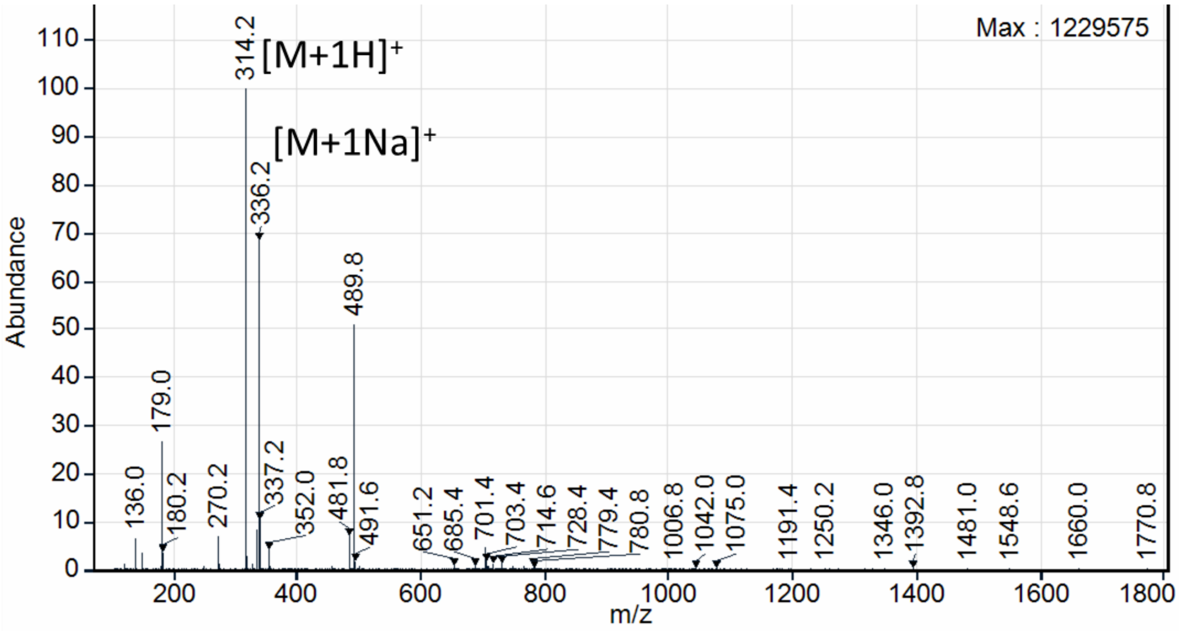

Figure S6 ESI-MS spectrum of the elution peak at t_R = 9.69 min (APD).

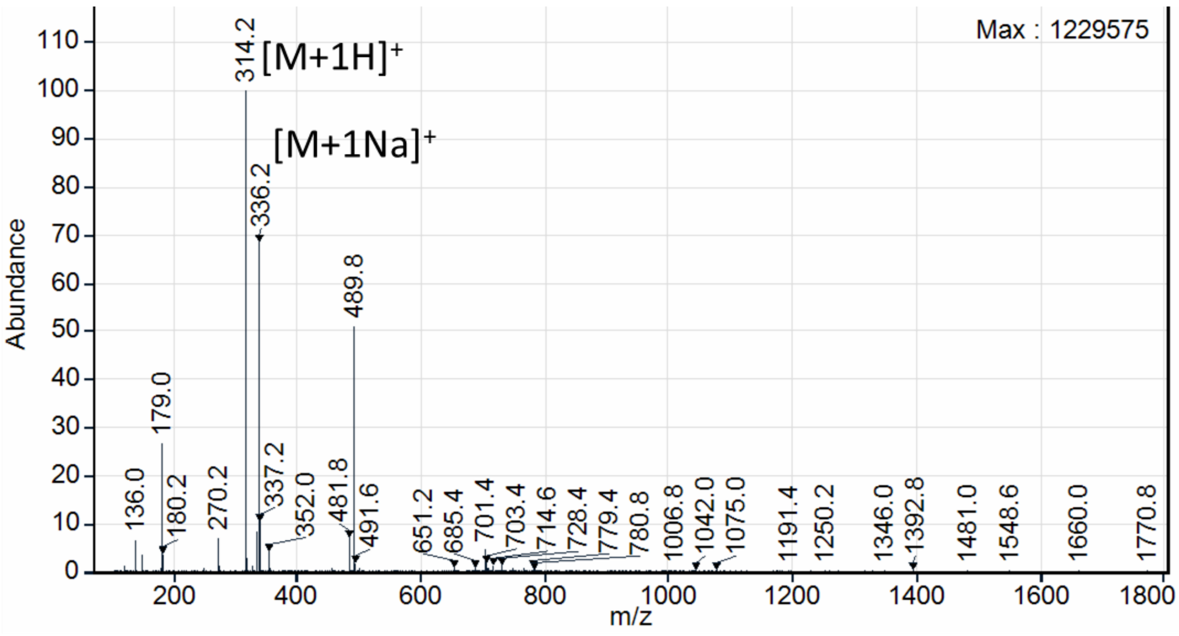

Figure S7 ESI-MS spectrum of the elution peak at t_R = 9.69 min (APD_Rec).

b) Structures synthesized with Fmoc-Gly TentaGel R HMPA Resin

Oligomer O1(hex)

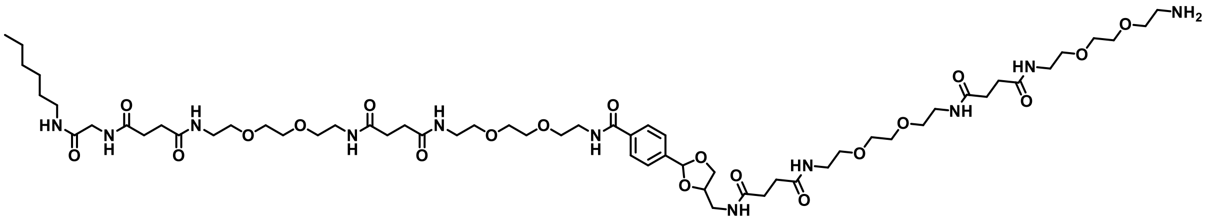

RP-HPLC: t_R = 7.14 min, 73% relative purity (UV), from 95/5 to 5/95 Vol.% Water/acetonitrile with 0.1 % formic acid in 20 min at 25 °C.

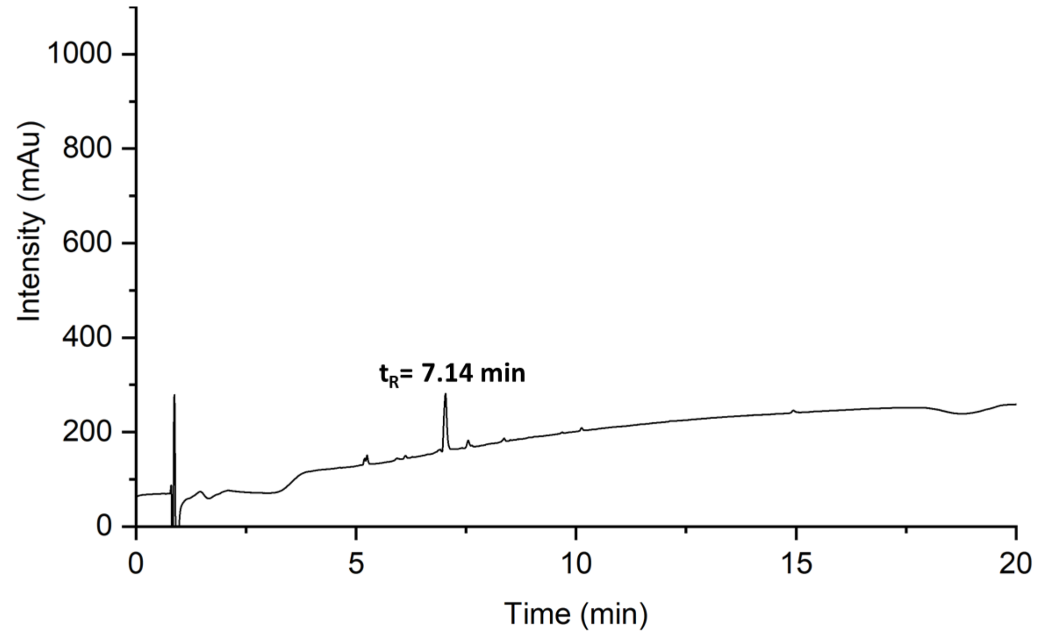

Figure S8 RP-HPLC chromatogram (from 95/5 to 5/95 Vol.% Water/acetonitrile with 0.1 % formic acid in 20 min at 25 °C) of O1(hex).

ESI-MS calc. for $C_{59}H_{10}1N_{11}O_{20}$: $[M+1H]^{1+}$ 1284.7, $[M+2H]^{2+}$ 642.9, $[M+3H]^{3+}$ 428.9; found 1284.8 $[M+1H]^{1+}$, 643.2 $[M+2H]^{2+}$, 429.2 $[M+3H]^{3+}$

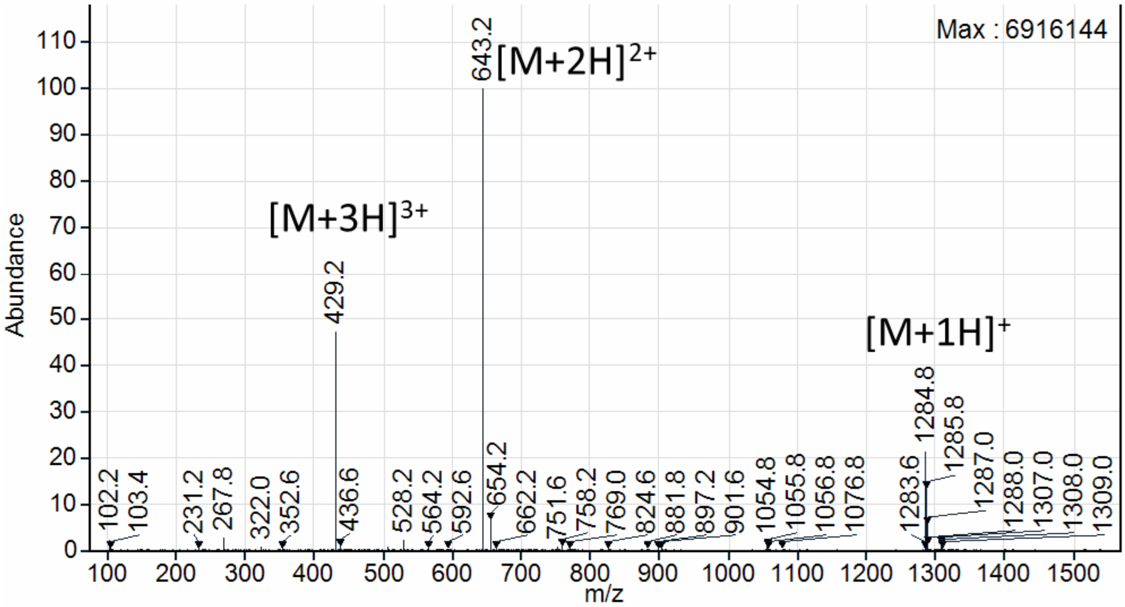

Figure S9 ESI-MS spectrum of the elution peak at t_R = 7.14 min (O1(hex))

*Fragment **1A***

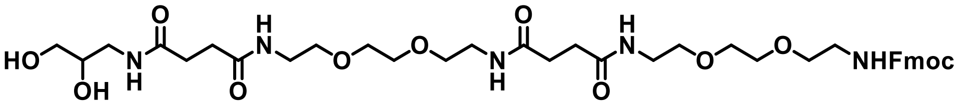

RP-HPLC: t_R = 9.21 min, 82 % relative purity (UV), from 95/5 to 5/95 Vol.% Water/acetonitrile with 0.1 % formic acid in 20 min at 25 °C.

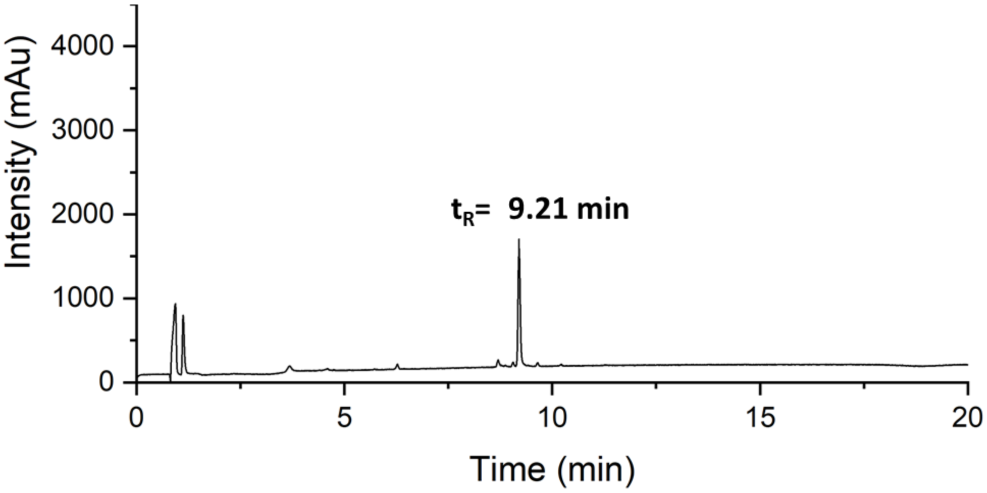

Figure S10 RP-HPLC chromatogram (from 95/5 to 5/95 Vol.% Water/acetonitrile with 0.1 % formic acid in 20 min at 25 °C) of 1A.

´

ESI-MS calc. for $C_{38}H_{55}N_5O_{12}$: $[M+1H]^{1+}$ 774.4, $[M+2H]^{2+}$ 387.7; found 774,4 $[M+1H]^{1+}$, 387.8 $[M+2H]^{2+}$

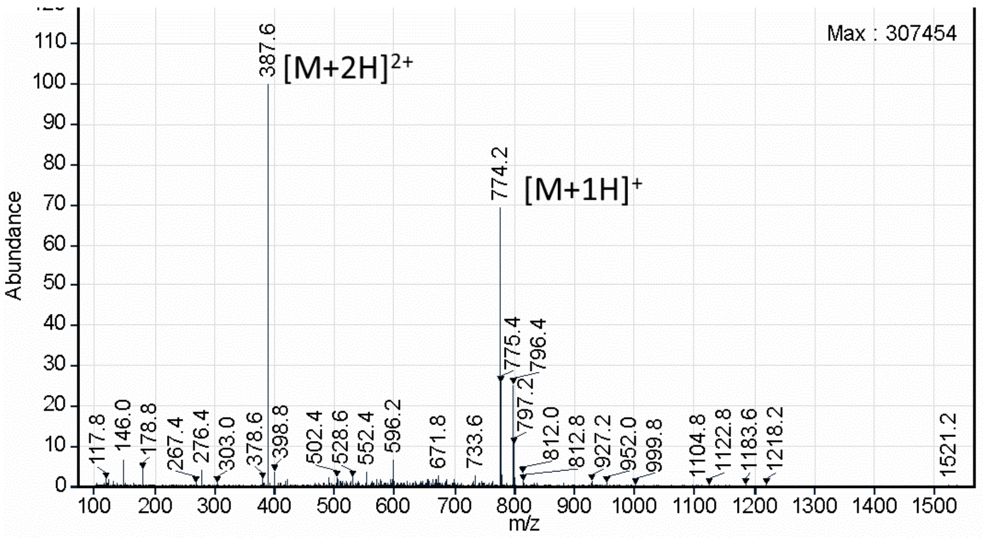

Figure S11 ESI-MS spectrum of the elution peak at t_R = 9.21 min (1A).

Side product of HMPA linker cleavage after 3 % TFA incubation of O1 for 60 min

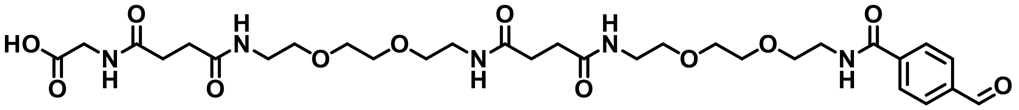

RP-HPLC: t_R = 9.21 min, 3 % relative purity (UV), from 95/5 to 5/95 Vol.% Water/acetonitrile with 0.1 % formic acid in 20 min at 25 °C.

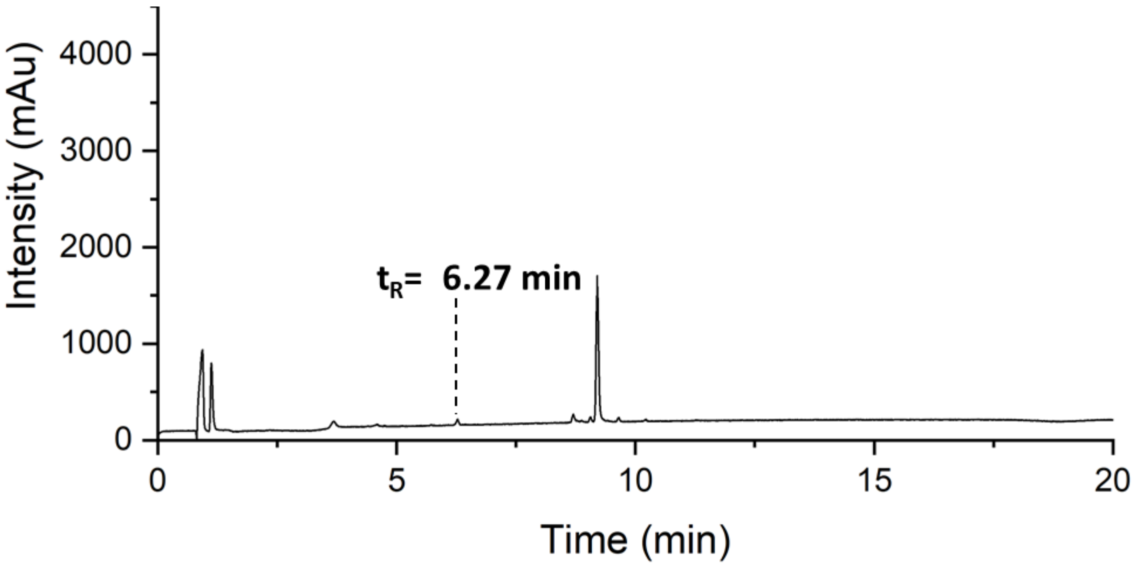

Figure S12 RP-HPLC chromatogram (from 95/5 to 5/95 Vol.% Water/acetonitrile with 0.1 % formic acid in 20 min at 25 °C) of the cleavage solution obtained after incubation of O1 with 3 Vol.% TFA for 60 min. The t_R = 6.27 min can be appointed to the side product obtained via HMPA linker cleavage.

ESI-MS calc. for $C_{30}H_{45}N_5O_{12}$: $[M+1H]^{1+}$ 668.3, $[M+2H]^{2+}$ 334.7; found 668.2 $[M+1H]^{1+}$, 334.6 $[M+2H]^{2+}$

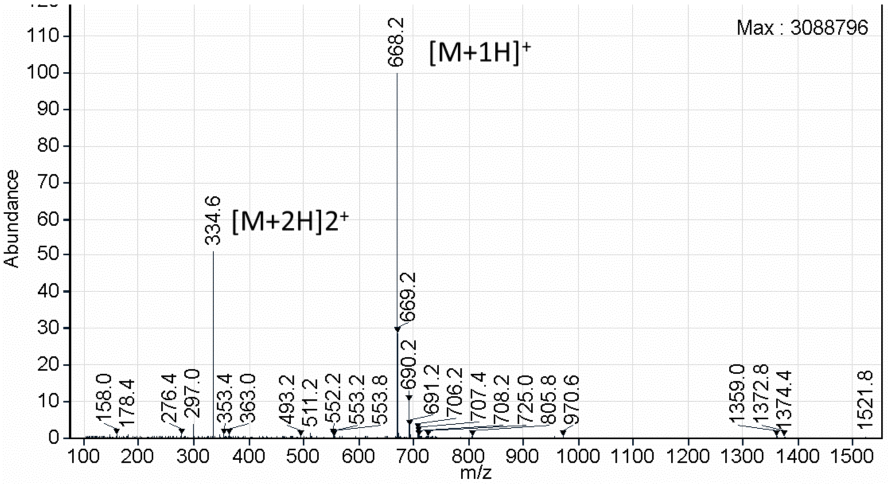

Figure S13 ESI-MS spectrum of the elution peak at t_R = 6.27 min (Side product – HMPA linker cleavage).

Fragment 1B(hex)

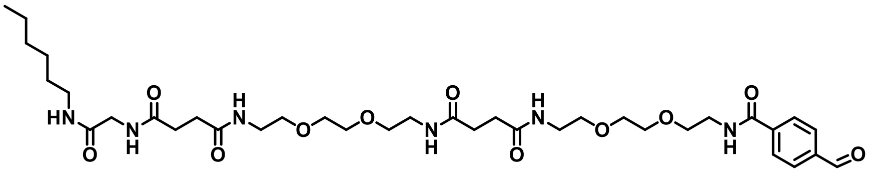

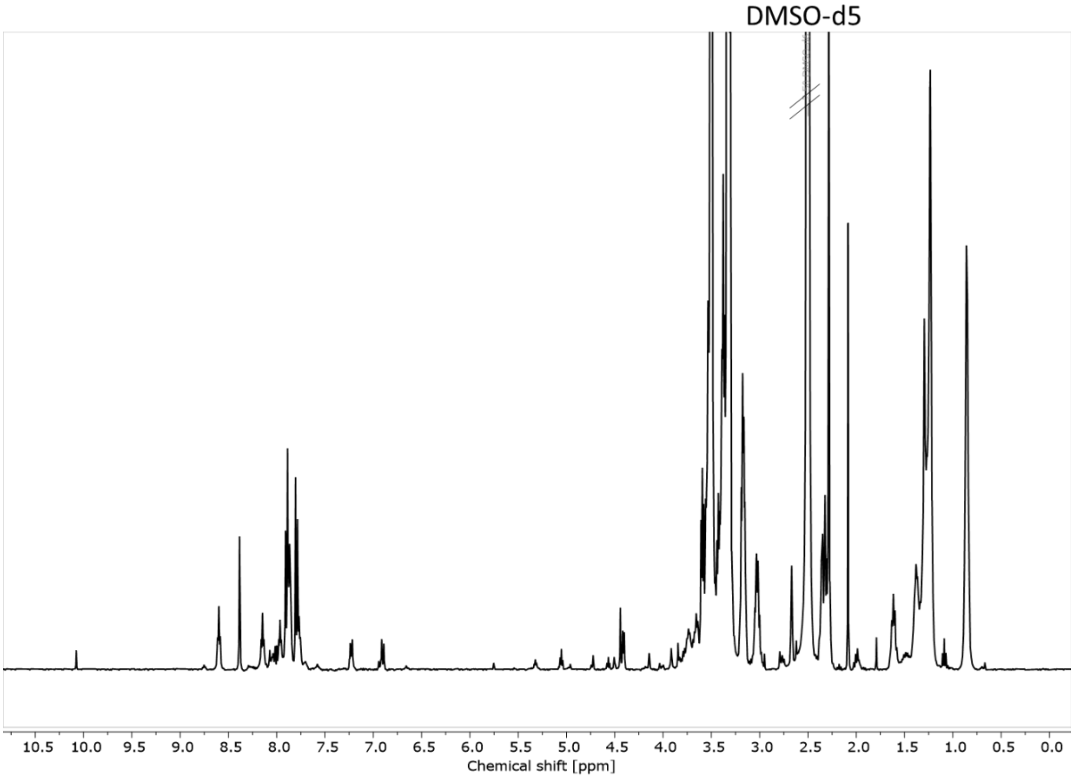

Figure S14 ^{1}H-NMR of 1B(hex) (400 MHz, DMSO, 25 °C).

RP-HPLC: t_R = 8.41 min, 98% relative purity (UV), from 95/5 to 5/95 Vol.% Water/acetonitrile with 0.1 % formic acid in 20 min at 25 °C.

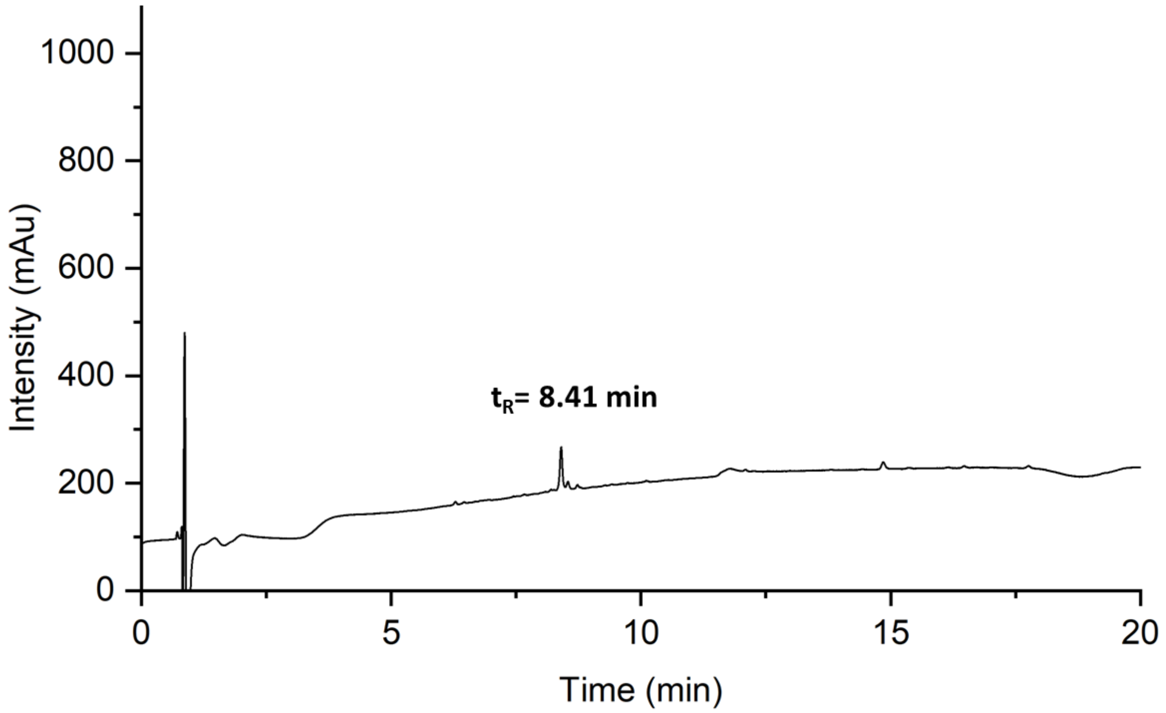

Figure S15 RP-HPLC chromatogram (from 95/5 to 5/95 Vol.% Water/acetonitrile with 0.1 % formic acid in 20 min at 25 °C) of 1B(hex).

ESI-MS calc. for $C_{36}H_{58}N_6O_{11}$: $[M+1H]^{1+}$ 751.4, $[M+2H]^{2+}$ 376.2; found 751.6 $[M+1H]^{1+}$, 376.4 $[M+2H]^{2+}$

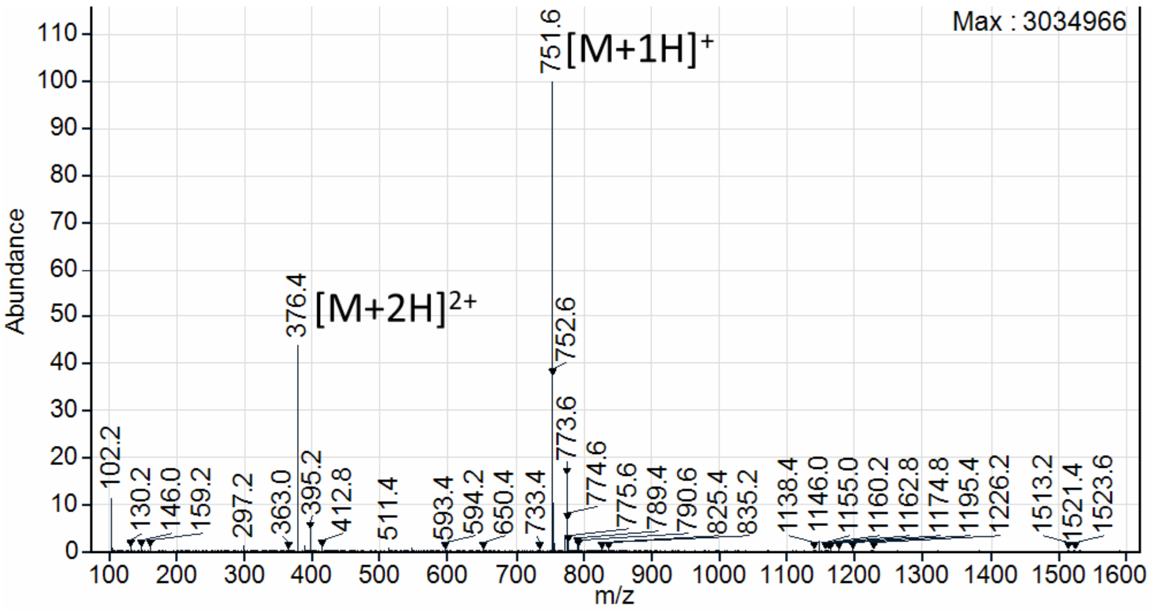

Figure S16 ESI-MS spectrum of the elution peak at t_R = 8.41 min (1B(hex)).

Structure 2(hex)

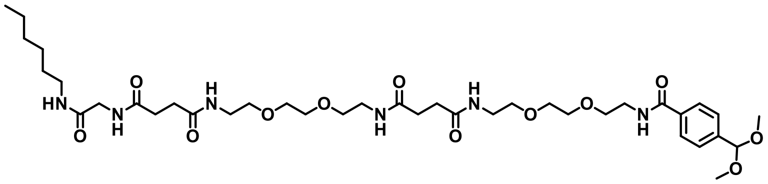

RP-HPLC: t_R = 8.80 min, 85 % relative purity (UV), from 95/5 to 5/95 Vol.% Water/acetonitrile with 0.1 % formic acid in 20 min at 25 °C.

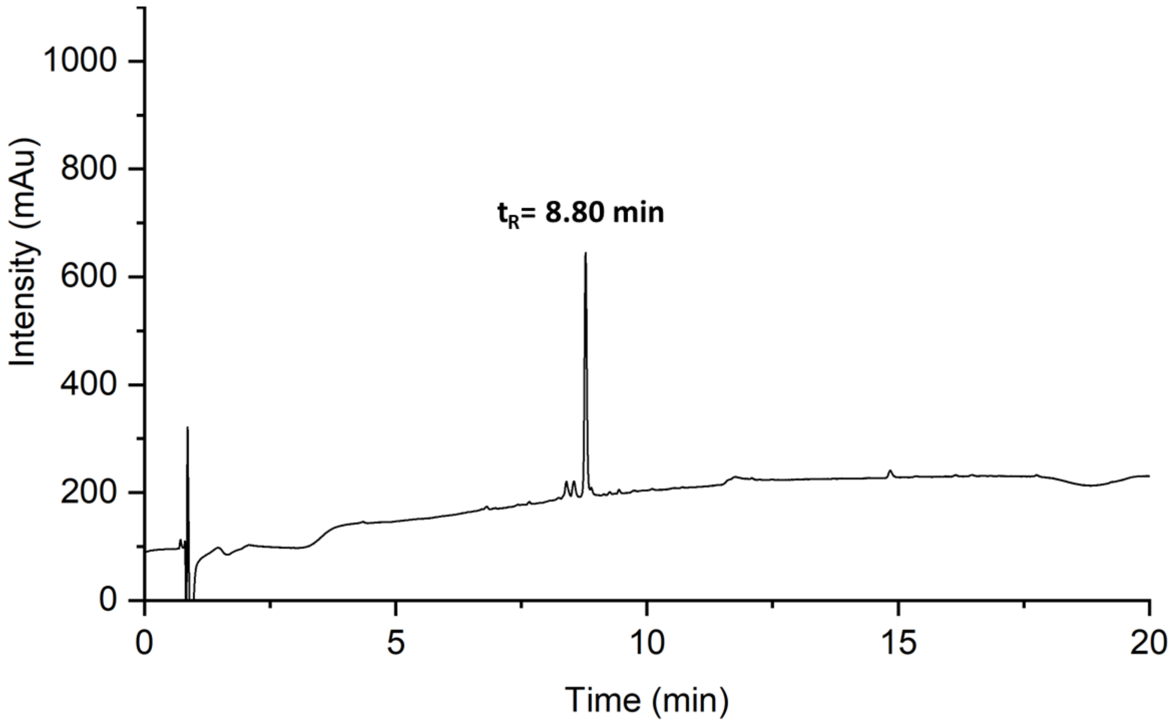

Figure S17 RP-HPLC chromatogram (from 95/5 to 5/95 Vol.% Water/acetonitrile with 0.1 % formic acid in 20 min at 25 °C) of 2(hex). The main byproduct at t_R = 8.41 min can be attributed to fragment 1B(hex).

ESI-MS calc. for $C_{38}H_{64}N_6O_{12}$: $[M+1Na]^{1+}$ 819.5; found 819.6 $[M+1Na]^{1+}$

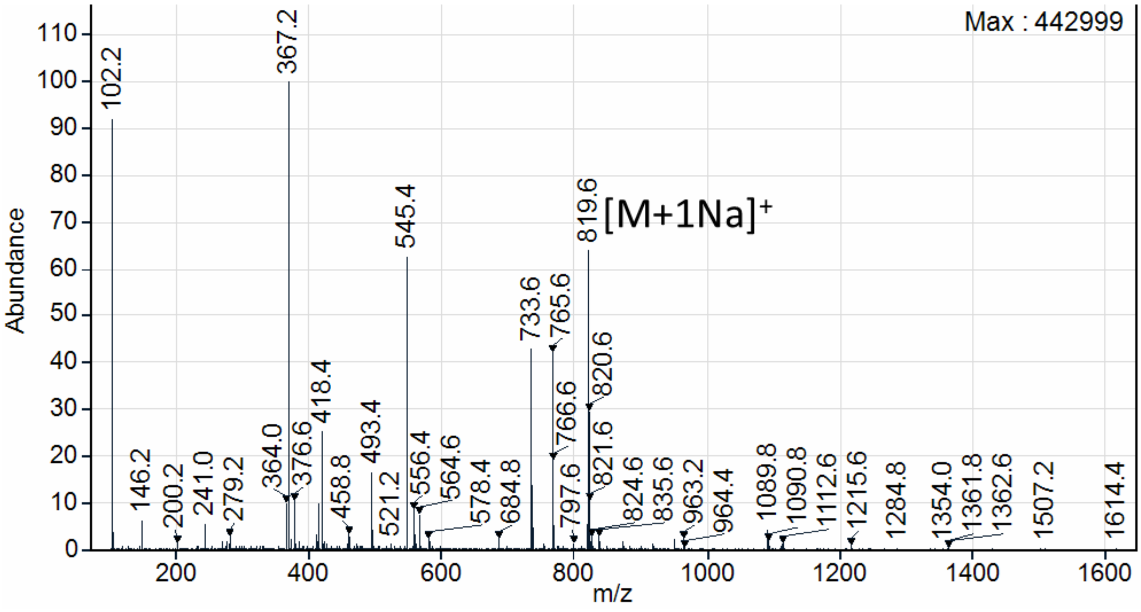

Figure S18 ESI-MS spectrum of the elution peak at t_R = 8.80 min 2(hex).

Structure 3(hex)

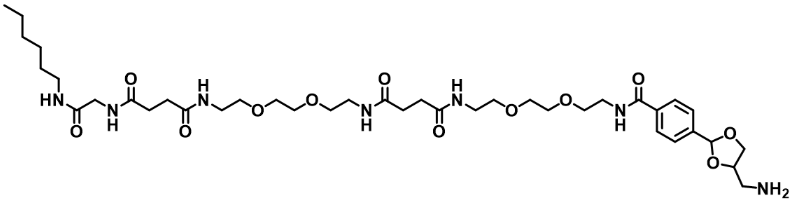

RP-HPLC: t_R = 7.10 min, 78% relative purity (UV), from 95/5 to 5/95 Vol.% Water/acetonitrile with 0.1 % formic acid in 20 min at 25 °C.

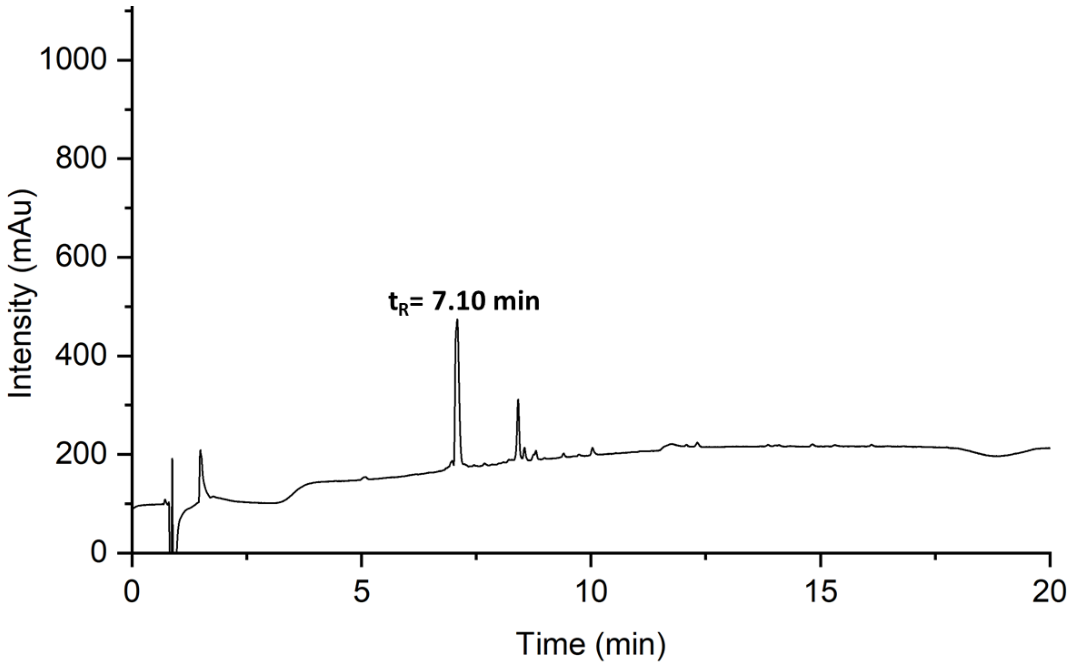

Figure S19 RP-HPLC chromatogram (from 95/5 to 5/95 Vol.% Water/acetonitrile with 0.1 % formic acid in 20 min at 25 °C) of 3(hex). The main byproduct at t_R = 8.41 min can be attributed to fragment 1B(hex).

ESI-MS calc. for $C_{39}H_{65}N_7O_{12}$: 824.5 $[M+1H]^{1+}$, 824.5 $[M+2H]^{2+}$ 412.7; found 824.6 $[M+1H]^{1+}$, 413.0 $[M+2H]^{2+}$

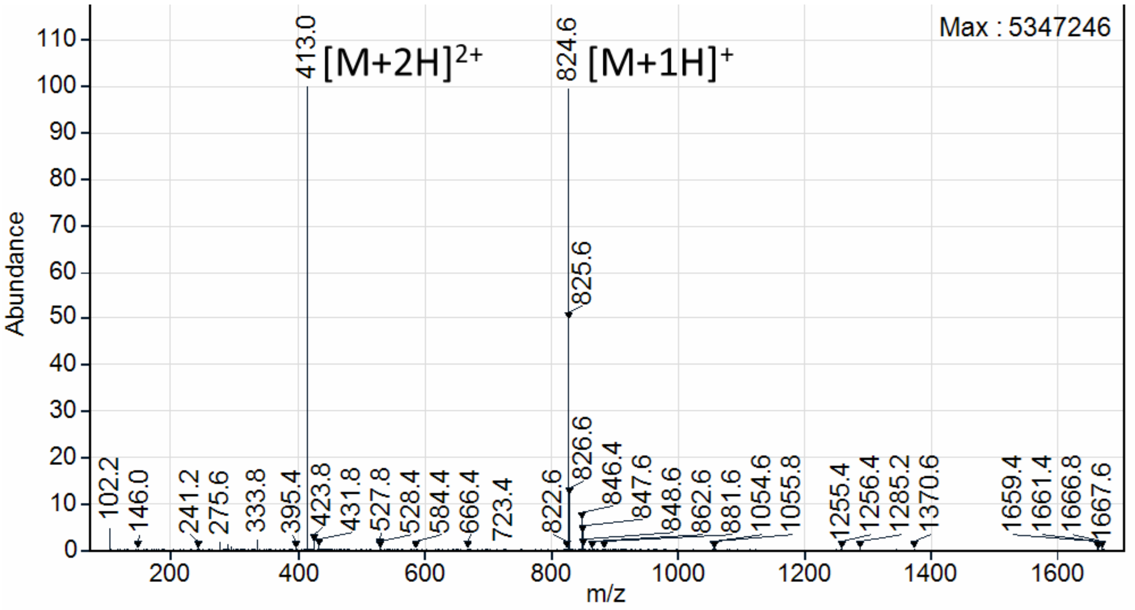

Figure S20 ESI-MS spectrum of the elution peak at t_R = 7.10 min 3(hex).

Structure 4(hex)

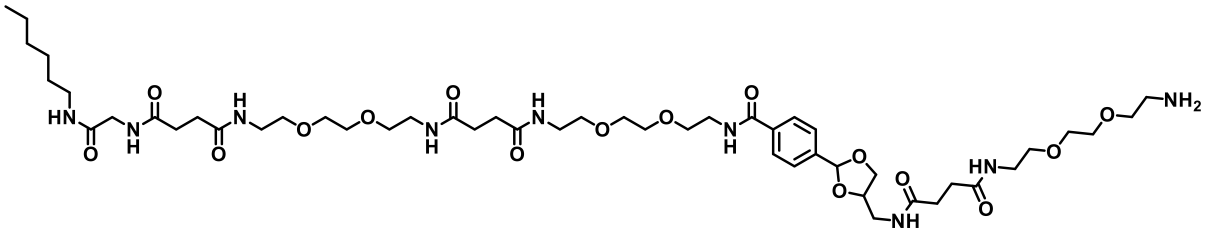

RP-HPLC: t_R = 7.14 min, 77% relative purity (UV), from 95/5 to 5/95 Vol.% Water/acetonitrile with 0.1 % formic acid in 20 min at 25 °C.

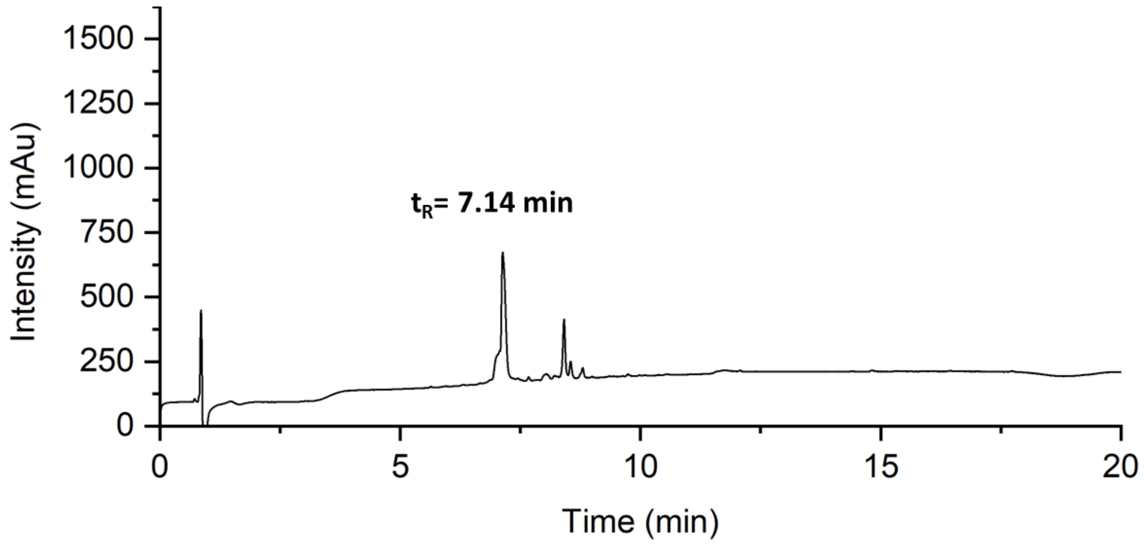

Figure S21 RP-HPLC chromatogram (from 95/5 to 5/95 Vol.% Water/acetonitrile with 0.1 % formic acid in 20 min at 25 °C) of 4(hex). The main byproduct at t_R = 8.41 min can be attributed to fragment 1B(hex).

ESI-MS calc. for $C_{49}H_{83}N_9O_{16}$: 1054.6 $[M+1H]^{1+}$, 527.8 $[M+2H]^{2+}$ 528.2; found 1054.6 $[M+1H]^{1+}$, 528.2 $[M+2H]^{2+}$

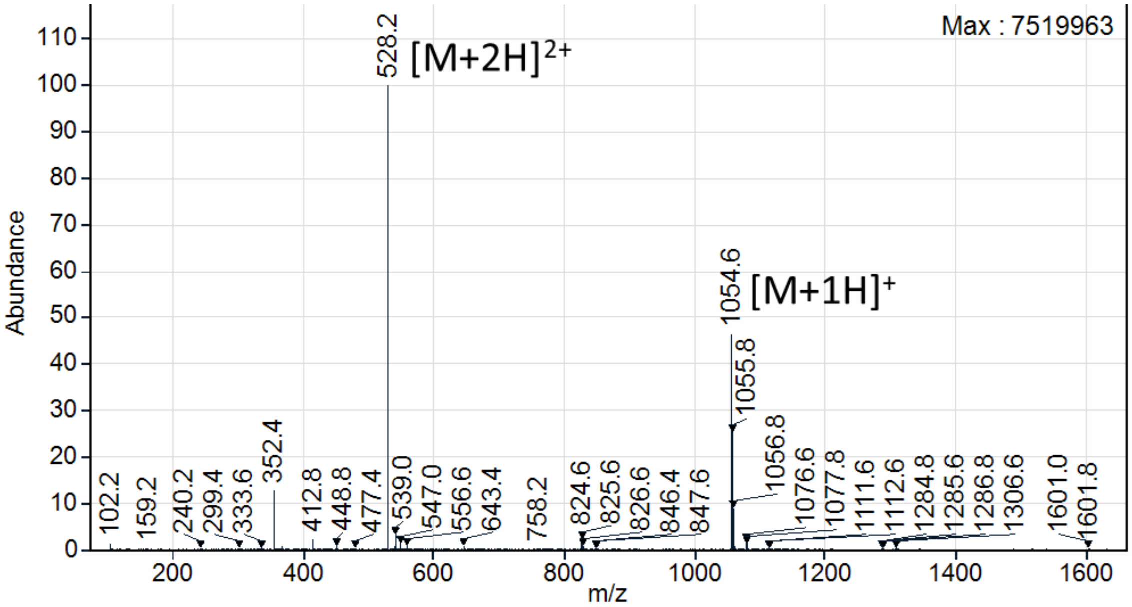

Figure S22 ESI-MS spectrum of the elution peak at t_R = 7.14 min 4(hex).

Oligomer fragment 4a.

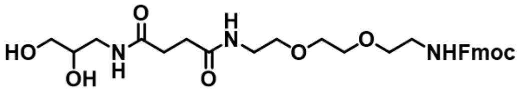

RP-HPLC: t_R = 9.41 min, 95% relative purity (UV), from 95/5 to 5/95 Vol.% Water/acetonitrile with 0.1 % formic acid in 20 min at 25 °C.

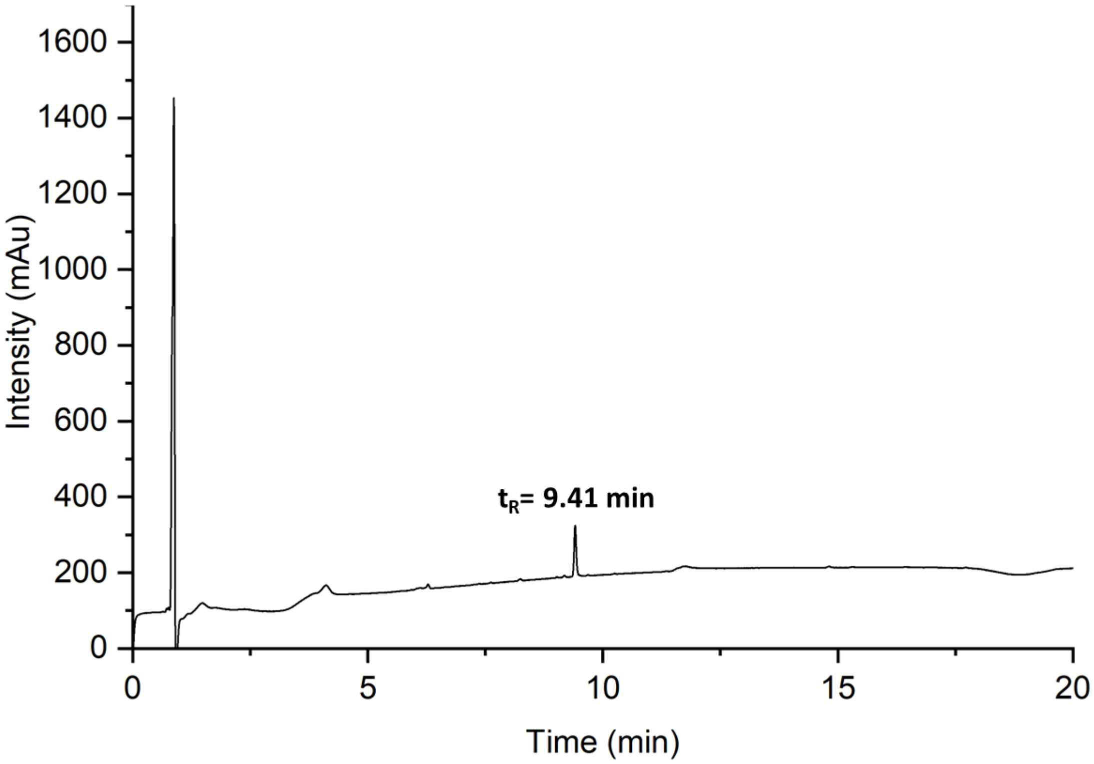

Figure S23 RP-HPLC chromatogram (from 95/5 to 5/95 Vol.% Water/acetonitrile with 0.1 % formic acid in 20 min at 25 °C) of 4a.

ESI-MS calc. for $C_{28}H_{37}N_3O_8$: $[M+1H]^{1+}$ 544.3; found 544.4 $[M+1H]^{1+}$

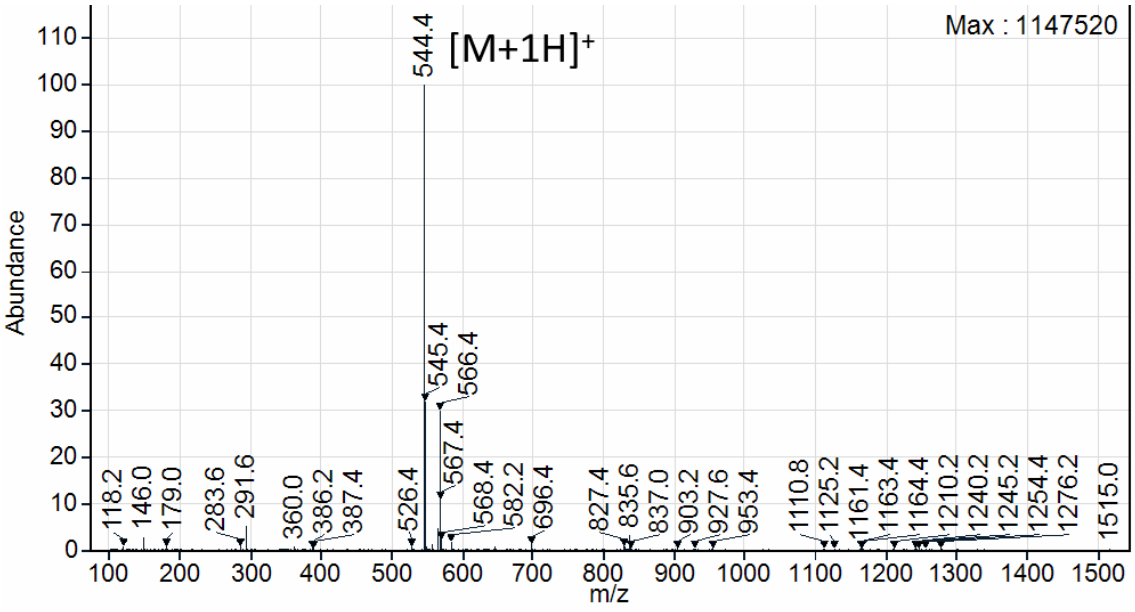

Figure S24 ESI-MS spectrum of the elution peak at t_R = 9.41 min(4a).

c) Dimeric structures synthesized with Tentagel S Amine DBA Resin

Dimer TDS2(1)

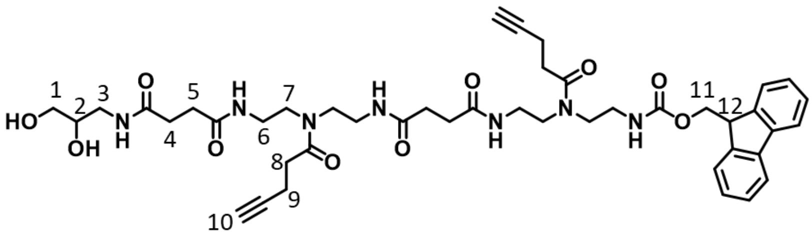

^{1}H NMR (400 MHz, D_2O): δ(ppm) = 7.78 – 6.78 (m, 8H, Ar-*H*), 4.28 – 2.99 (m, 24H, *H*-1-3, *H*-6-7, *H*-11-12), 2.75 – 2.02 (m, 18H, *H*-4, *H*-5, *H8-10*)

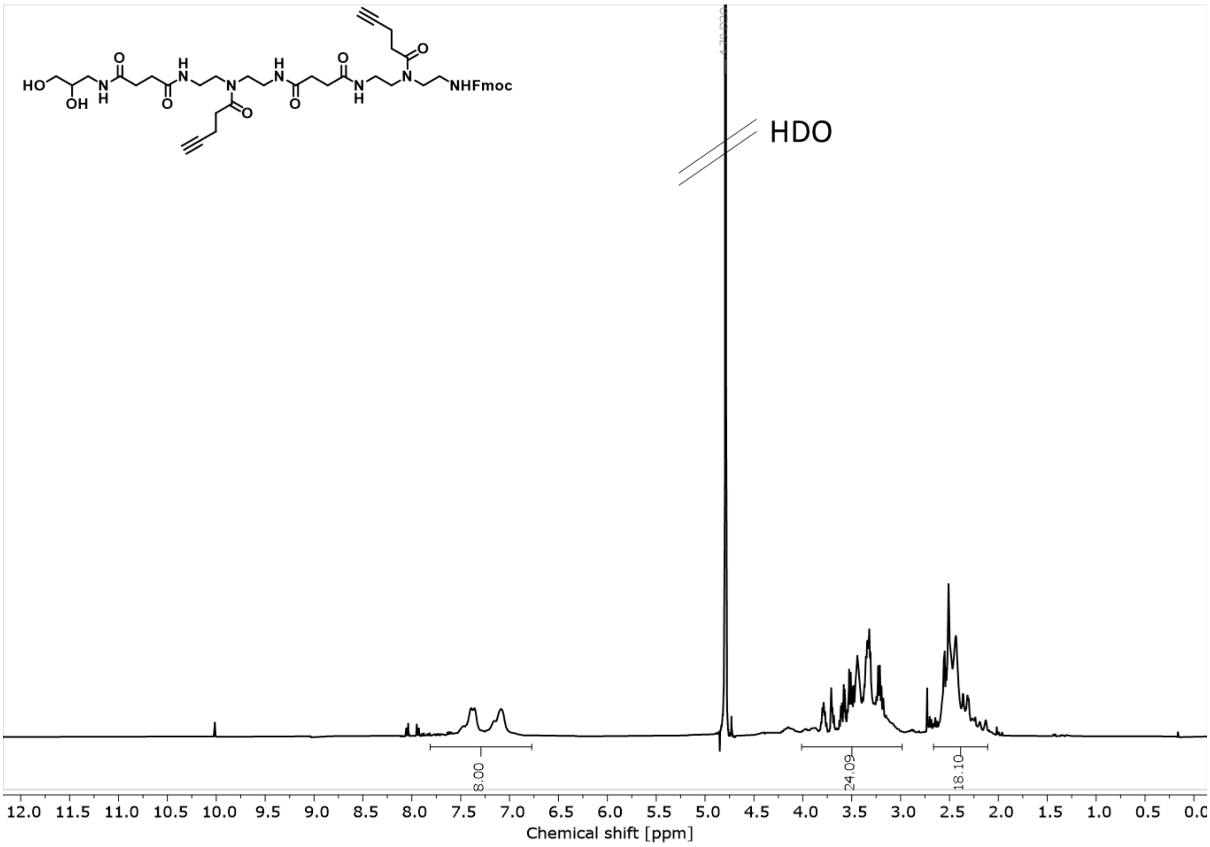

Figure S25 ^{1}H-NMR of TDS2(1) (400 MHz, D_2O, 25 °C).

RP-HPLC: t_R = 9.63 min, 86% relative purity (UV), from 95/5 to 5/95 Vol.% Water/acetonitrile with 0.1 % formic acid in 20 min at 25 °C.

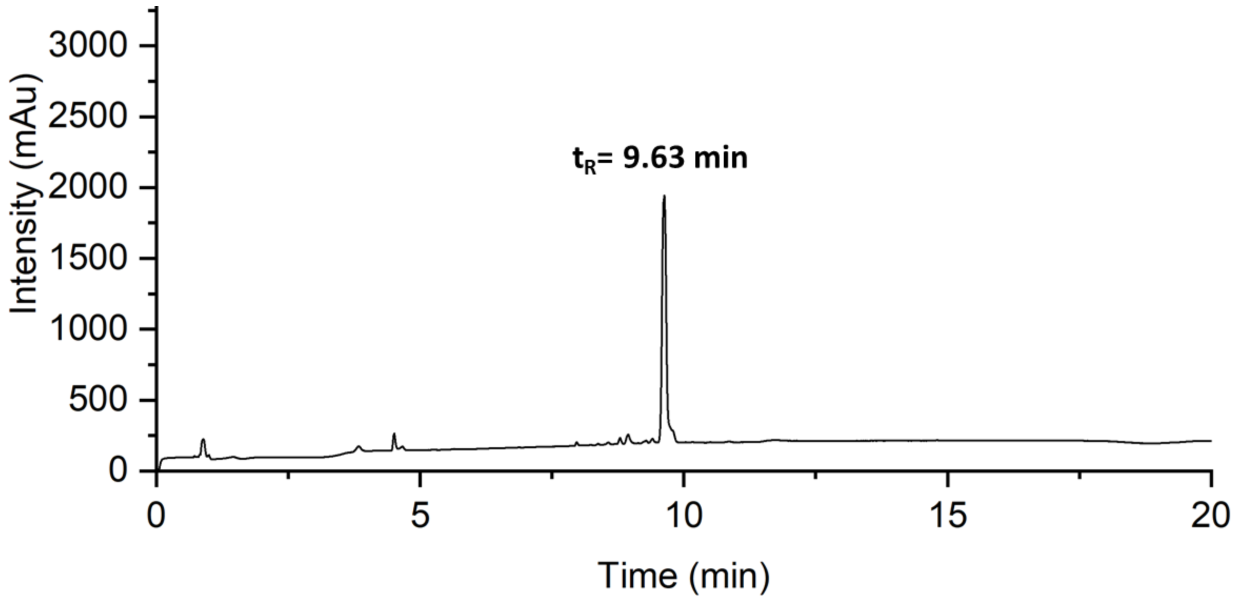

Figure S26 RP-HPLC chromatogram (from 95/5 to 5/95 Vol.% Water/acetonitrile with 0.1 % formic acid in 20 min at 25 °C) of TDS2(1).

ESI-MS calc. for $C_{44}H_{57}N_7O_{10}$: $[M+1H]^{1+}$ 422.7, $[M+2H]^{2+}$ 844.4; found 844.6 $[M+1H]^{1+}$, 422.8 $[M+2H]^{2+}$

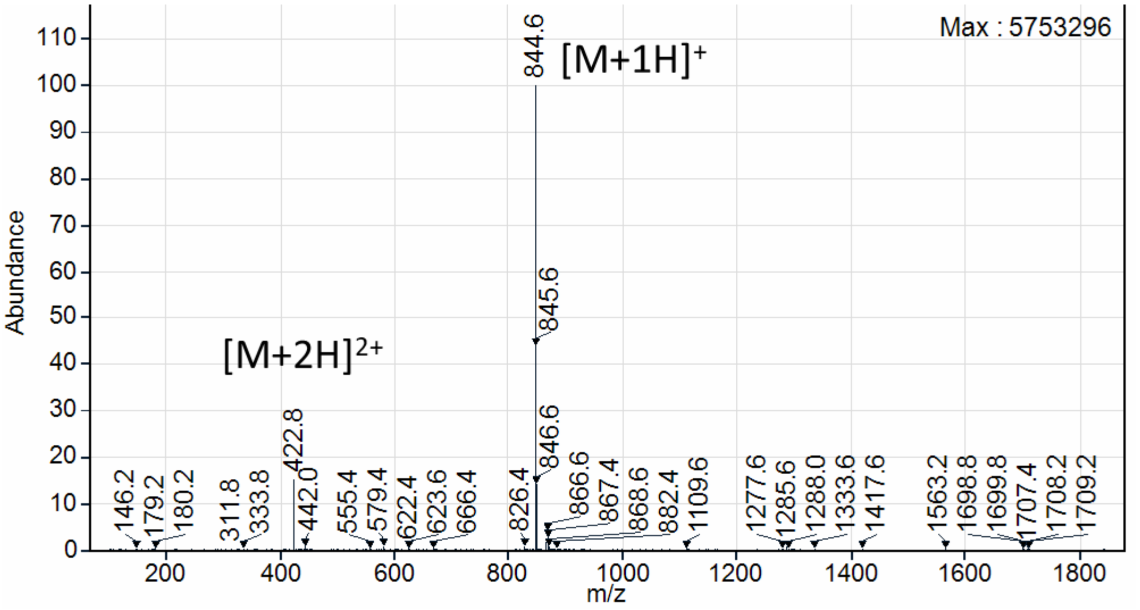

Figure S27 ESI-MS spectrum of the elution peak at t_R = 9.63 min (TDS2(1)).

Dimer TDS2(2)

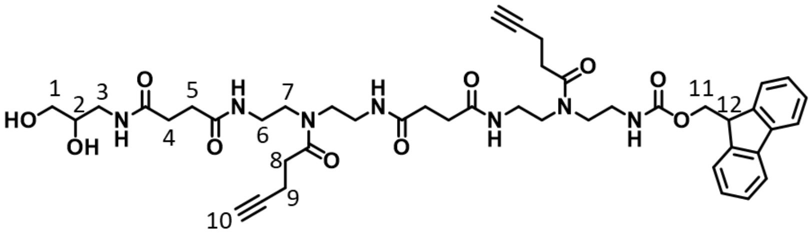

^{1}H NMR (400 MHz, D_2O): δ(ppm) = 7.63 – 6.80 (m, 8H, Ar-*H*), 4.28 – 2.99 (m, 24H, *H*-1-3, *H*-6-7, *H11-12*), 2.75 – 2.01 (m, 18H, *H*-4, *H*-5, *H8-10*)

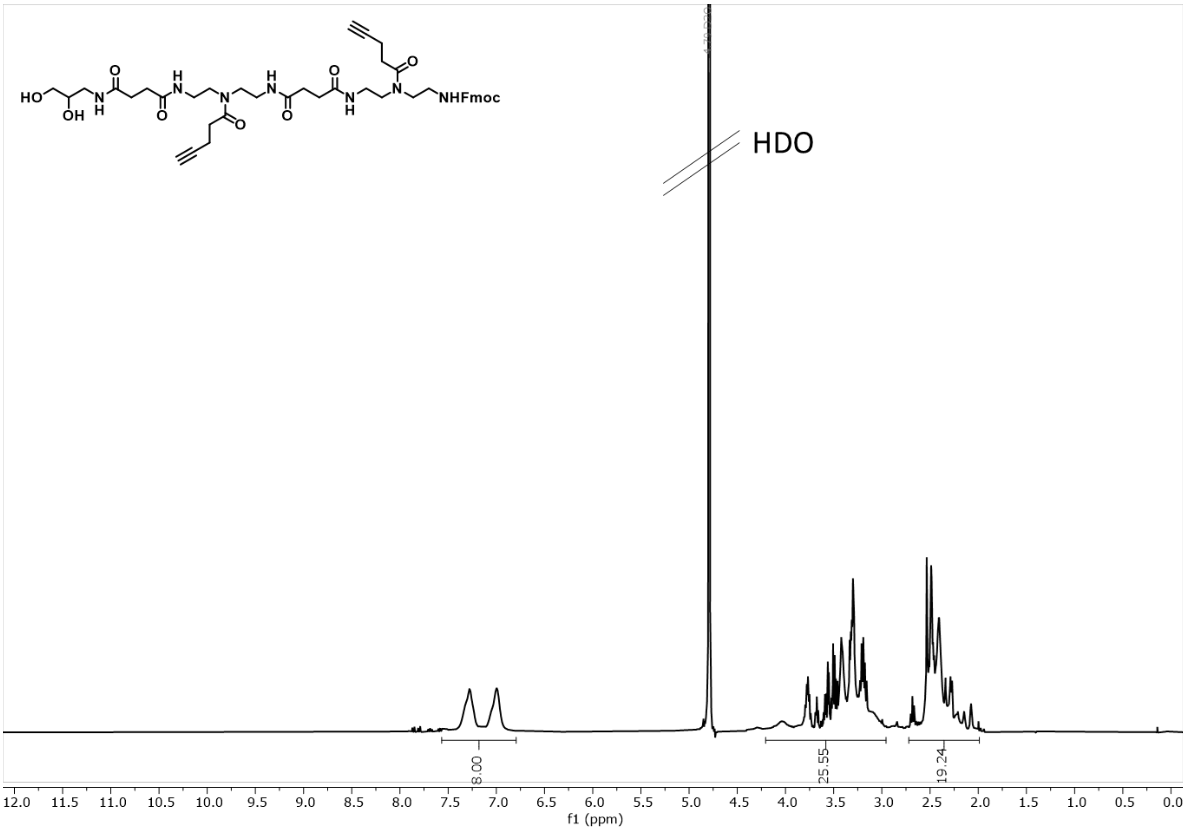

Figure S28 ^{1}H-NMR of TDS2(2) (400 MHz, D_2O, 25 °C).

RP-HPLC: t_R = 9.63 min, 98% relative purity (UV), from 95/5 to 5/95 Vol.% Water/acetonitrile with 0.1 % formic acid in 20 min at 25 °C.

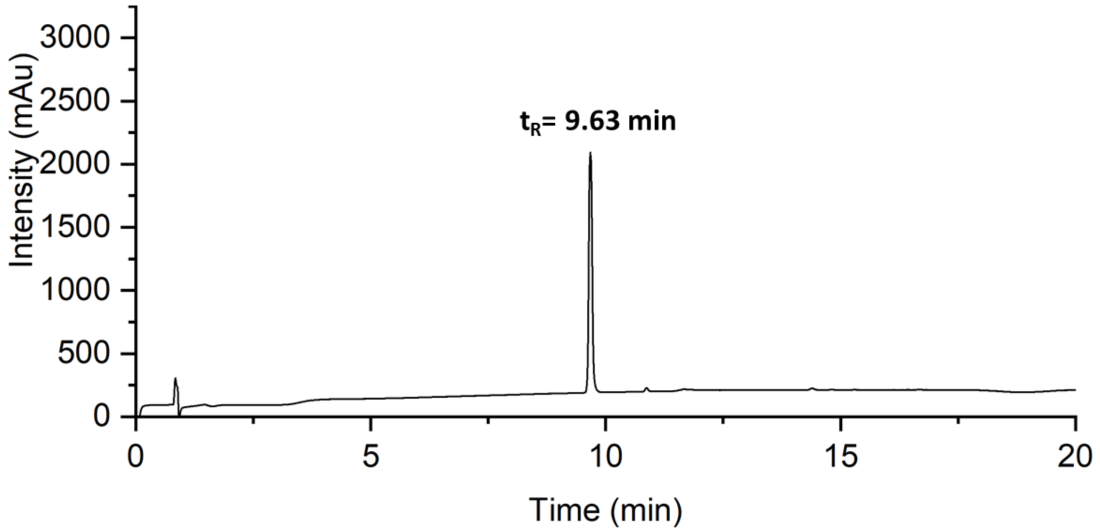

Figure S29 RP-HPLC chromatogram (from 95/5 to 5/95 Vol.% Water/acetonitrile with 0.1 % formic acid in 20 min at 25 °C) of TDS2(2).

ESI-MS calc. for $C_{44}H_{57}N_7O_{10}$: $[M+1H]^{1+}$ 422.7, $[M+2H]^{2+}$ 844.4; found 844.6 $[M+1H]^{1+}$, 422.8 $[M+2H]^{2+}$

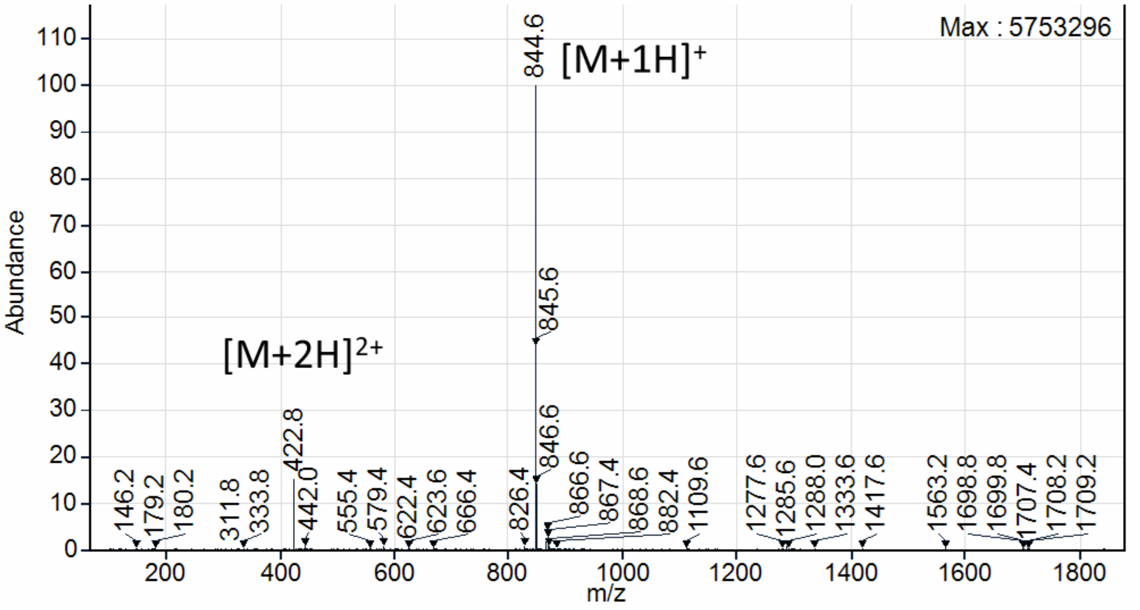

Figure S30 ESI-MS spectrum of the elution peak at t_R = 9.63 min (TDS2(2)).

Dimer EDS2(1)

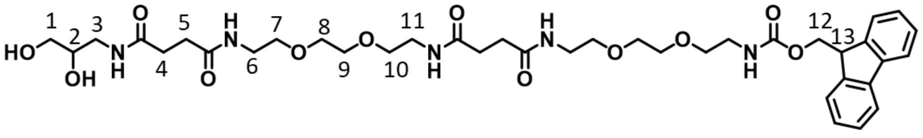

^{1}H NMR (400 MHz, D2O): δ(ppm) = 7.65 – 6.99 (m, 8H, Ar-*H*), 4.20 (m, 2H, *H*-12), 3.98 (m, 1H, *H*-13), 3.85 – 3.10 (m, 29H, *H*-1-3, *H*-6-11), 2.60 – 2.38 (m, 8H, *H*-4, *H*-5)

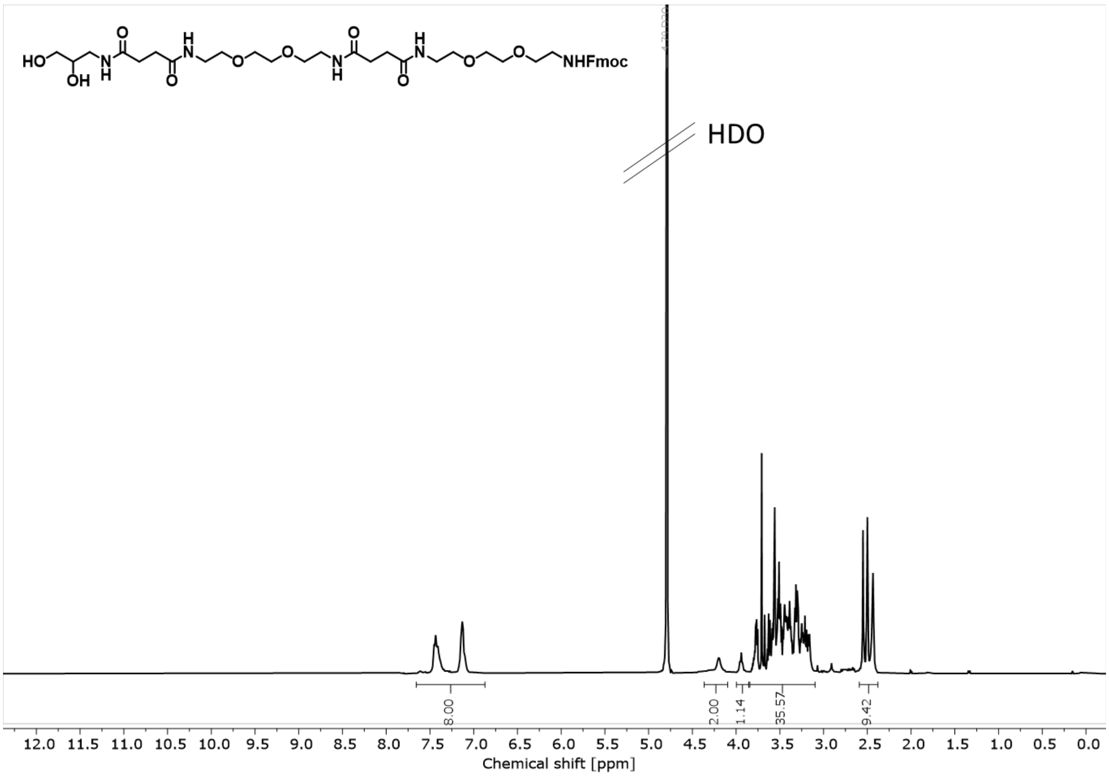

Figure S31 ^{1}H-NMR of EDS2(1) (400 MHz, D_2O, 25 °C).

RP-HPLC: t_R = 9.18 min, 95% relative purity (UV), from 95/5 to 5/95 Vol.% Water/acetonitrile with 0.1 % formic acid in 20 min at 25 °C.

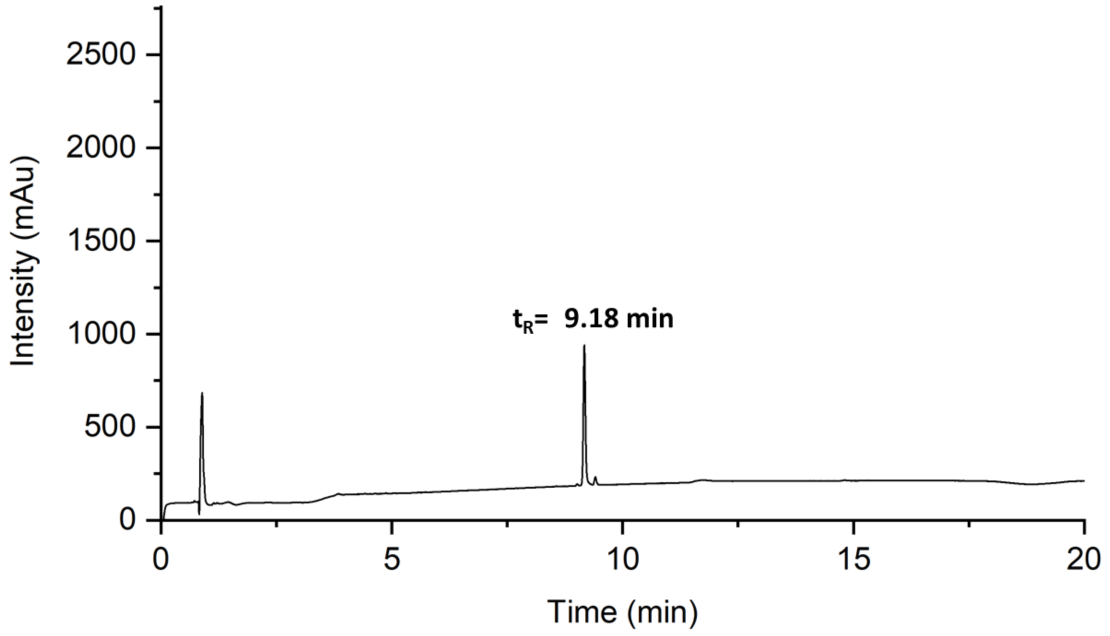

Figure S32 RP-HPLC chromatogram (from 95/5 to 5/95 Vol.% Water/acetonitrile with 0.1 % formic acid in 20 min at 25 °C) of EDS2(1).

ESI-MS calc. for $C_{38}H_{55}N_5O_{12}$: $[M+1H]^{1+}$ 387.7, $[M+2H]^{2+}$ 774.4; found 774.4 $[M+1H]^{1+}$, 387.8 $[M+2H]^{2+}$

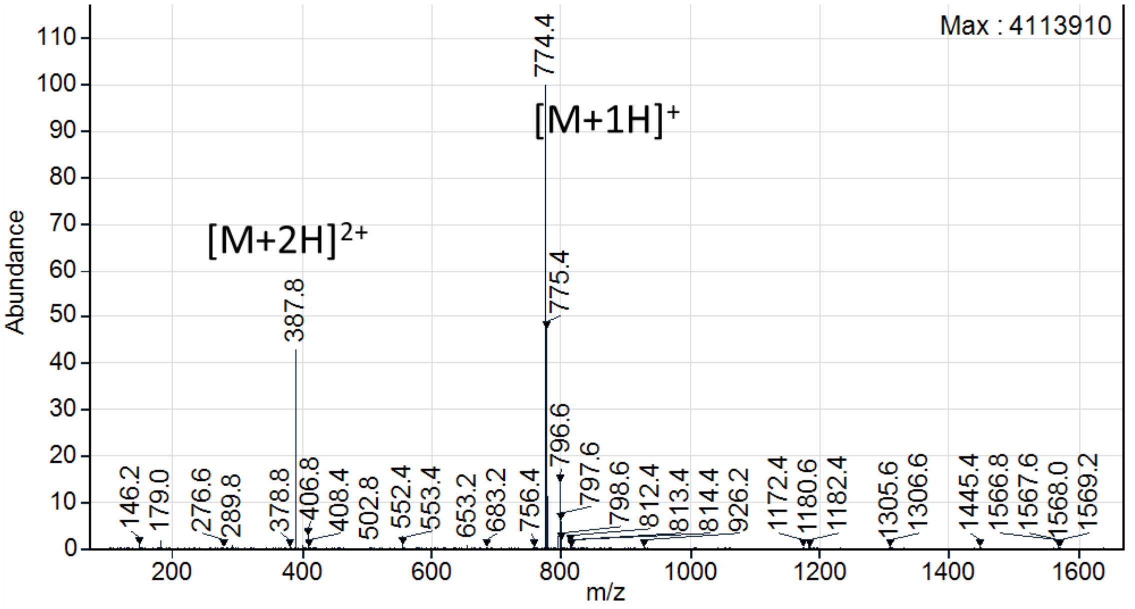

Figure S33 ESI-MS spectrum of the elution peak at t_R = 9.18 min (EDS2(1)).

Dimer EDS2(2)

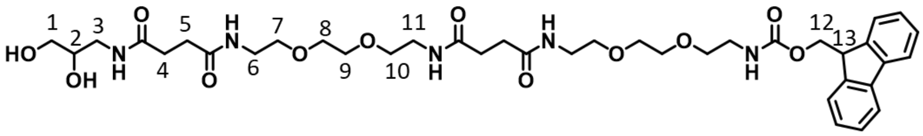

^{1}H NMR (400 MHz, D2O): δ(ppm) = 7.65 – 7.00 (m, 8H, Ar-*H*), 4.20 (m, 2H, *H*-12), 3.95 (m, 1H, *H*-13), 3.83 – 3.10 (m, 29H, *H*-1-3, *H*-6-11), 2.57 – 2.37 (m, 8H, *H*-4, *H*-5)

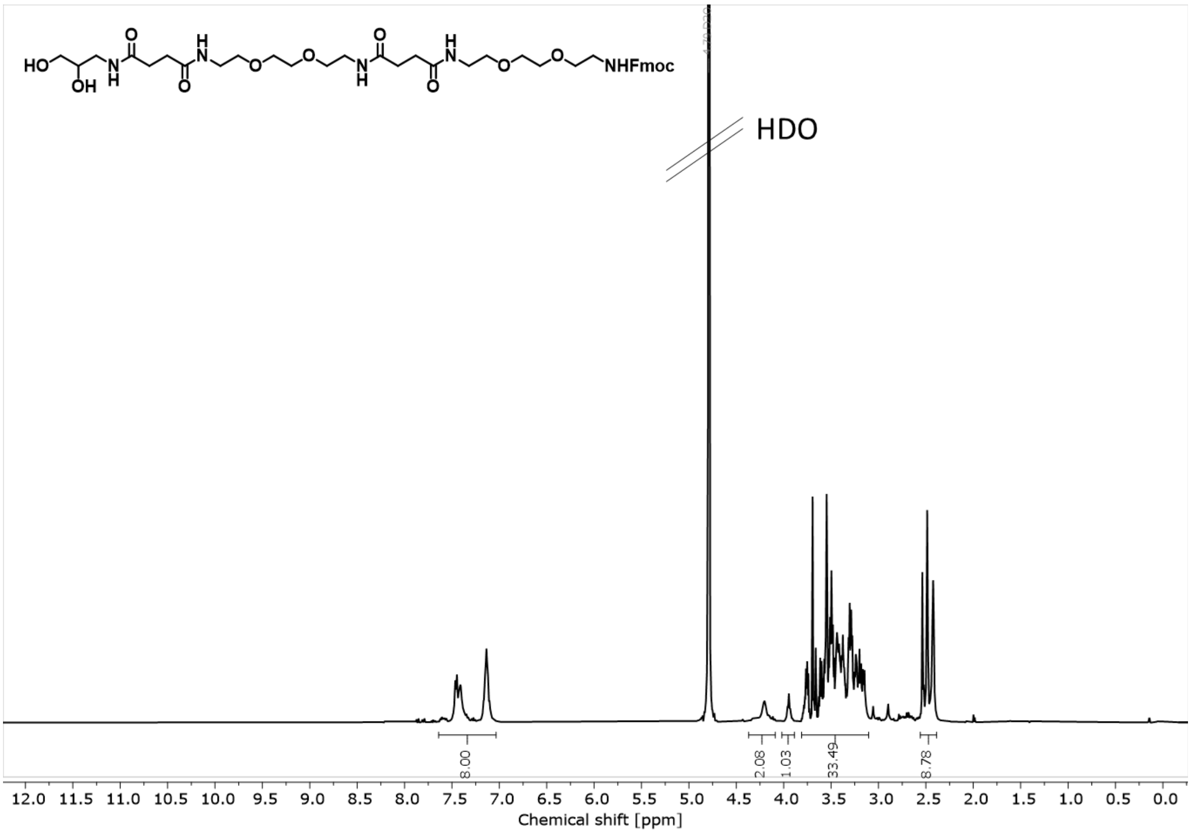

Figure S34 ^{1}H-NMR of EDS2(2) (400 MHz, D_2O, 25 °C).

RP-HPLC: t_R = 9.18 min, 91% relative purity (UV), from 95/5 to 5/95 Vol.% Water/acetonitrile with 0.1 % formic acid in 20 min at 25 °C.

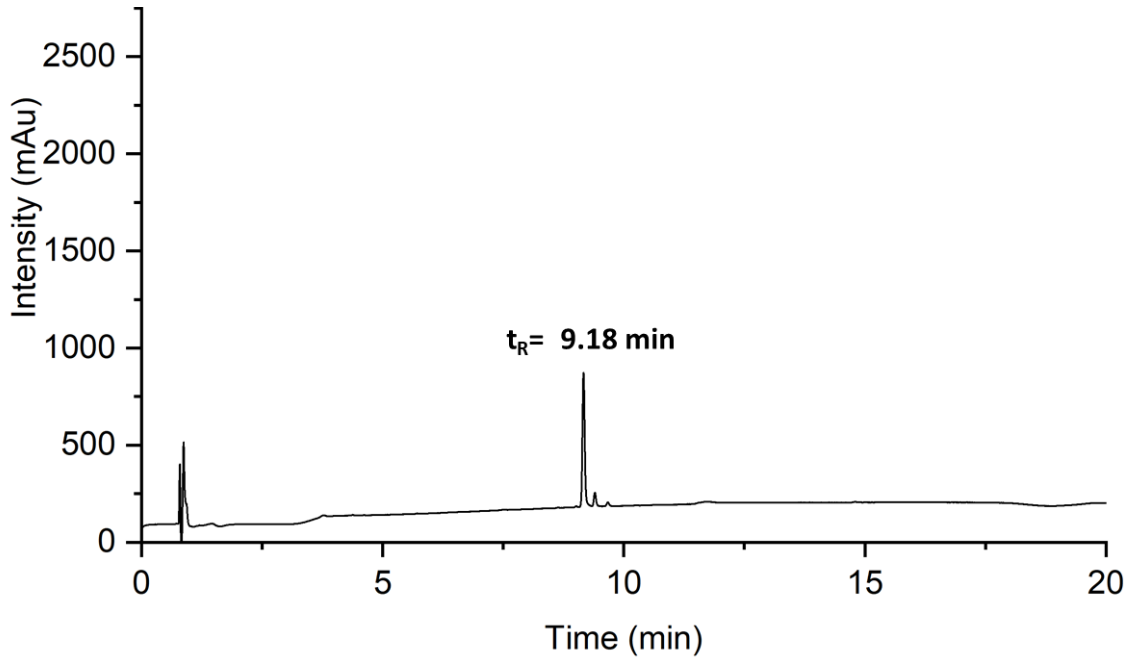

Figure S35 RP-HPLC chromatogram (from 95/5 to 5/95 Vol.% Water/acetonitrile with 0.1 % formic acid in 20 min at 25 °C) of EDS2(2).

ESI-MS calc. for $C_{38}H_{55}N_5O_{12}$: $[M+1H]^{1+}$ 387.7, $[M+2H]^{2+}$ 774.4; found 774.4 $[M+1H]^{1+}$, 387.8 $[M+2H]^{2+}$

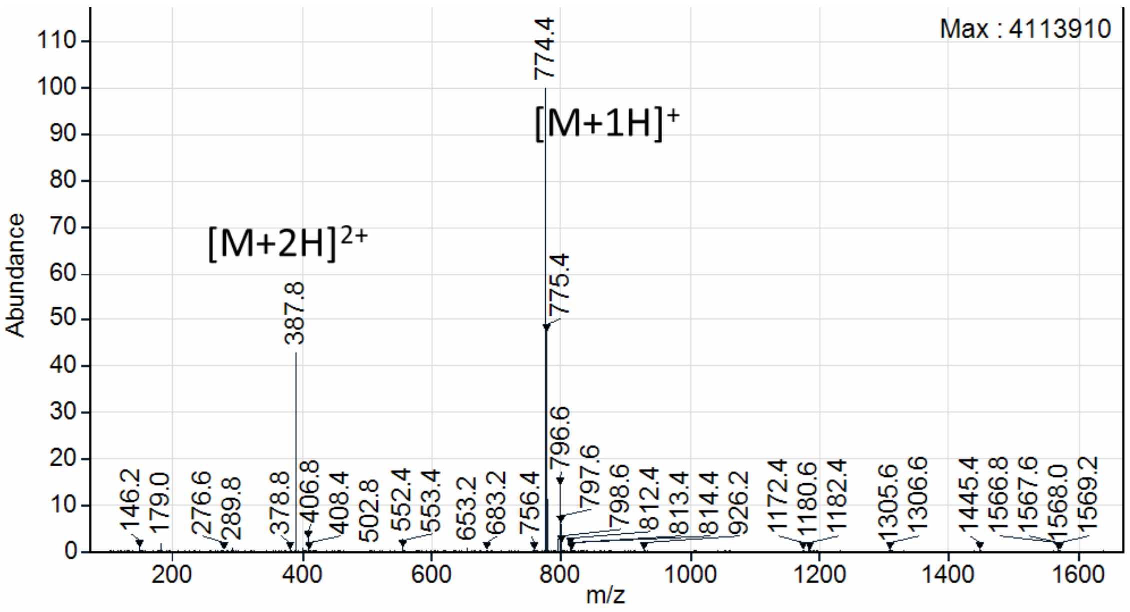

Figure S36 ESI-MS Spectrum of the elution peak at t_R = 9.18 min (EDS2(2)).

d) *Optimization of on-resin DBA cleavage*

TFA incubation of Tentagel® R HMPA resin preloaded with Fmoc-glycine

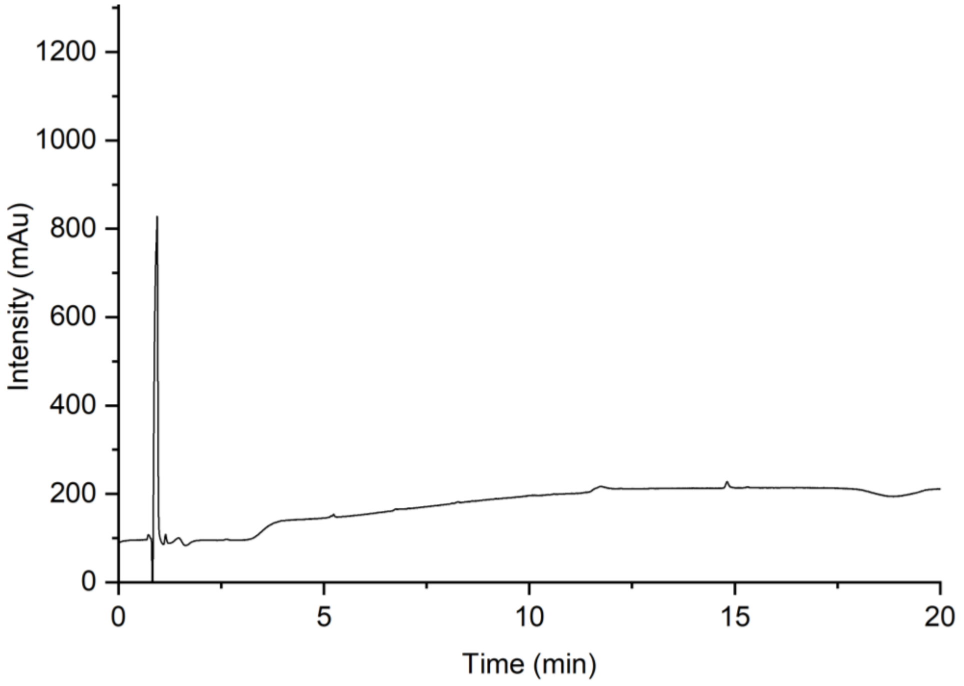

Figure S37 RP-HPLC chromatogram (from 95/5 to 5/95 Vol.% Water/acetonitrile with 0.1 % formic acid in 20 min at 25 °C) of the 3 Vol.% TFA solution after incubation with Tentagel® R HMPA resin preloaded with Fmoc-glycine for 60 min.

TFA incubation of resin-bound ***O1***

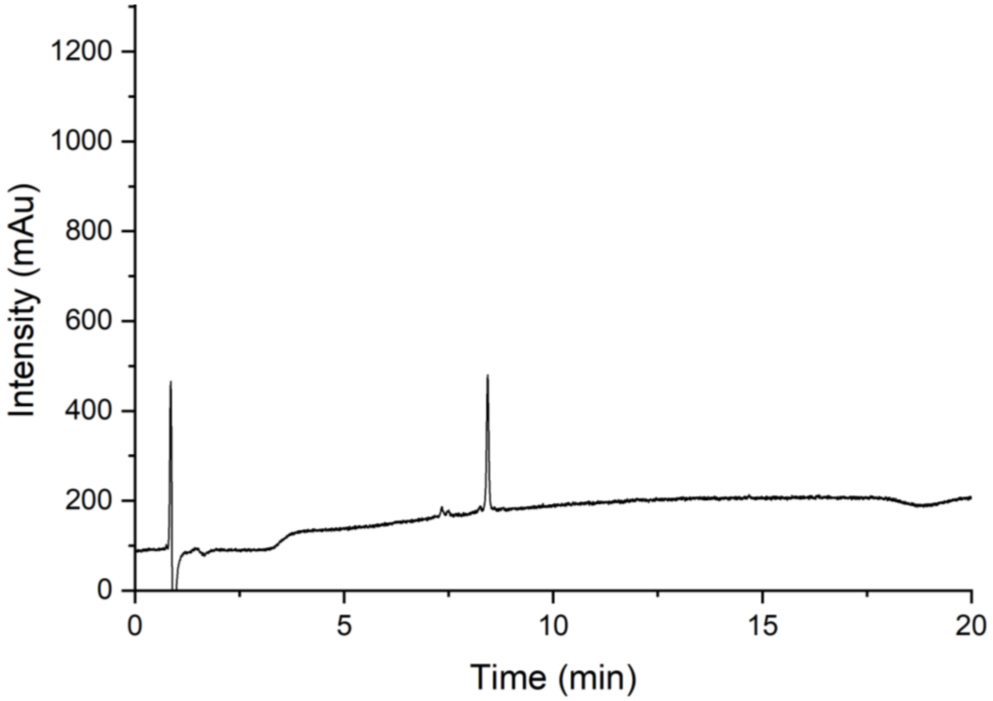

Figure S38 RP-HPLC chromatogram (from 95/5 to 5/95 Vol.% Water/acetonitrile with 0.1 % formic acid in 20 min at 25 °C) of O1(hex) after incubation of resin-bound O1 with 1 Vol.% TFA in water for 30 min and subsequent cleavage with hexylamine. The non-hydrolyzed oligomer O1(hex) can be found at t_R = 7.14 as well as the desired hydrolyzed fragment 1B(hex) at t_R = 8.41 min.

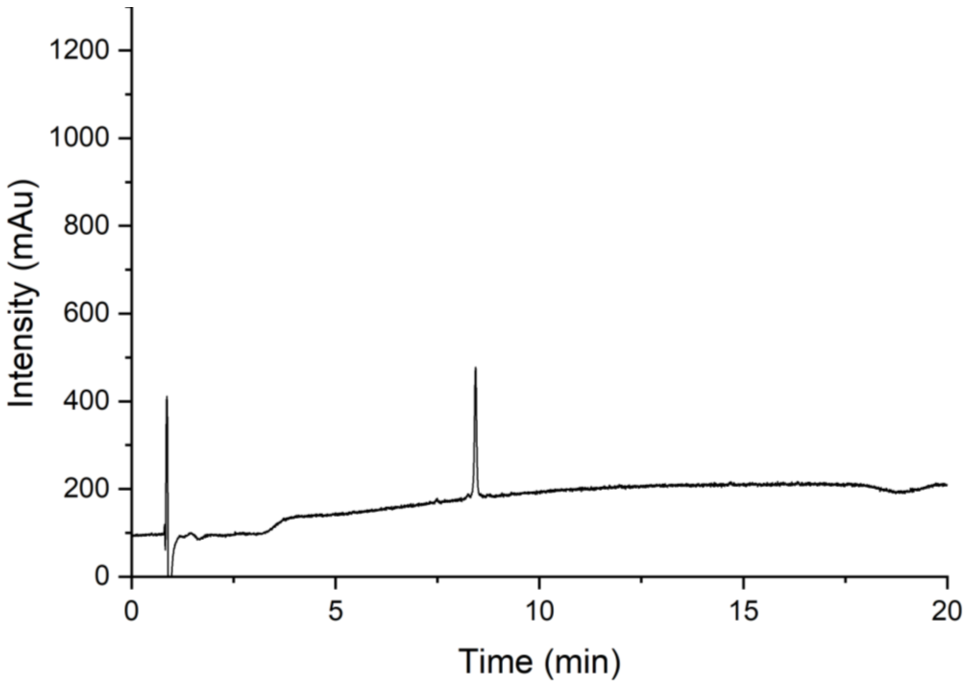

Figure S39 RP-HPLC chromatogram (from 95/5 to 5/95 Vol.% Water/acetonitrile with 0.1 % formic acid in 20 min at 25 °C) of O1(hex) after incubation of resin-bound O1 with 1 Vol.% TFA in water for 2x30 min and subsequent cleavage with hexylamine. The non-hydrolyzed oligomer O1(hex) can be found at t_R = 7.14 as well as the desired hydrolyzed fragment 1B(hex) at t_R = 8.41 min.

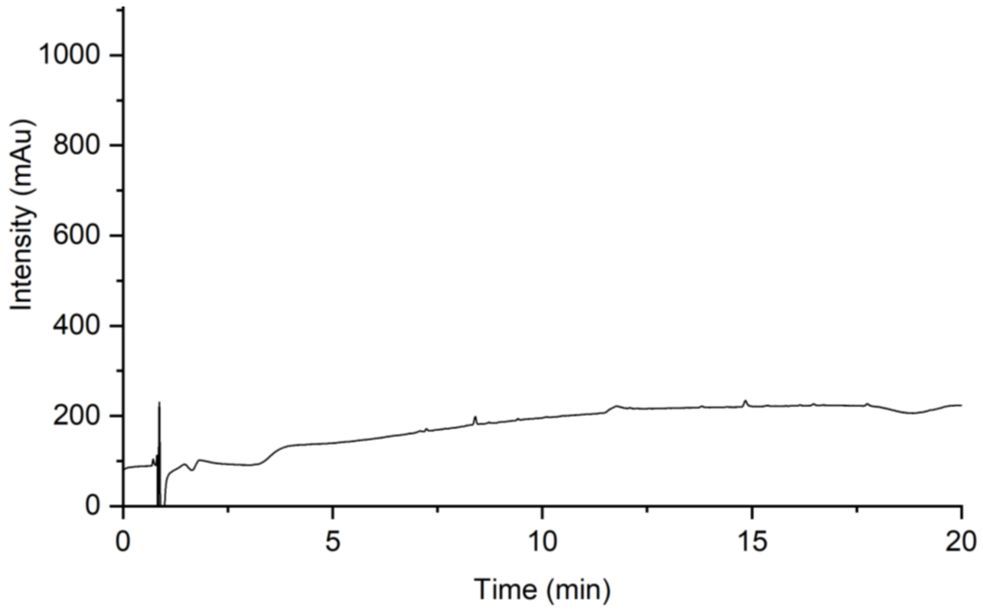

Figure S40 RP-HPLC chromatogram (from 95/5 to 5/95 Vol.% Water/acetonitrile with 0.1 % formic acid in 20 min at 25 °C) of O1(hex) after incubation of resin-bound O1 with 3 Vol.% TFA in water for 30 min and subsequent cleavage with hexylamine. The non-hydrolyzed oligomer O1(hex) can be found at t_R = 7.14 as well as the desired hydrolyzed fragment 1B(hex) at t_R = 8.41 min.

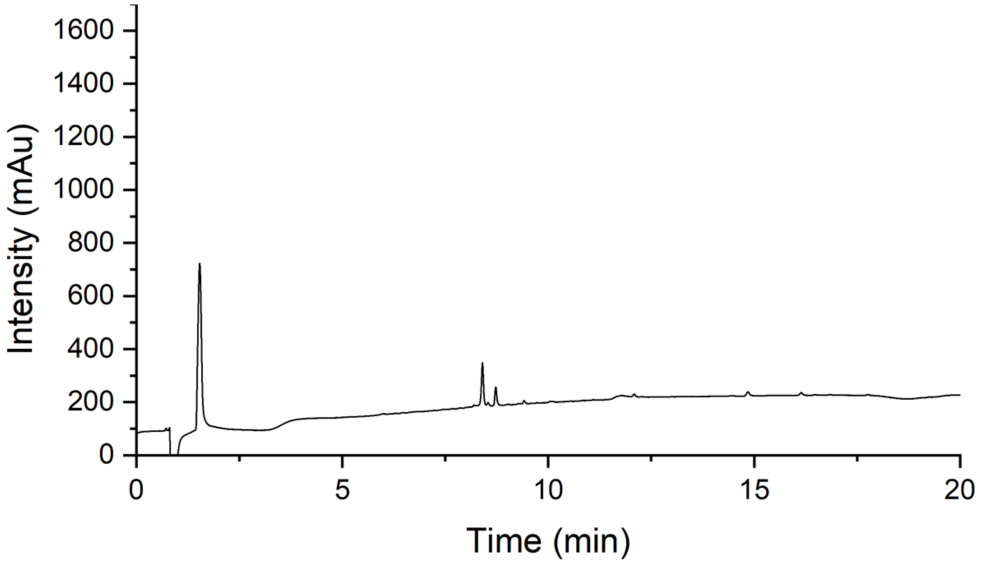

Figure S41 RP-HPLC chromatogram (from 95/5 to 5/95 Vol.% Water/acetonitrile with 0.1 % formic acid in 20 min at 25 °C) of O1(hex) after incubation of resin-bound O1 with 3 Vol.% TFA in water for 2x30 min and subsequent cleavage with hexylamine. The desired hydrolyzed fragment 1B(hex) can be found at t_R = 8.41 min.

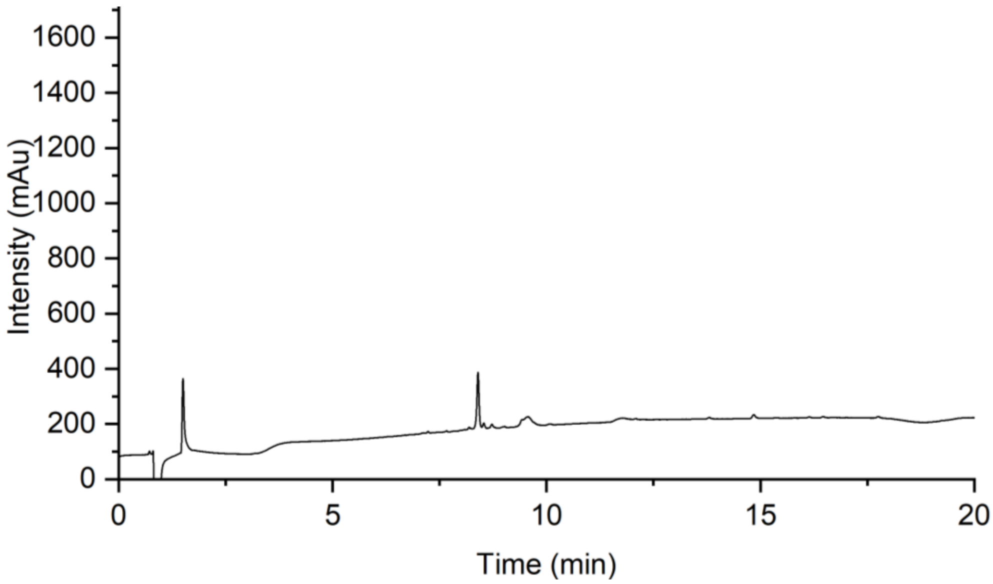

Figure S42 RP-HPLC chromatogram (from 95/5 to 5/95 Vol.% Water/acetonitrile with 0.1 % formic acid in 20 min at 25 °C) of O1(hex) after incubation of resin-bound O1 with 5 Vol.% TFA in water for 30 min and subsequent cleavage with hexylamine. The desired hydrolyzed fragment 1B(hex) can be found at t_R = 8.41 min.

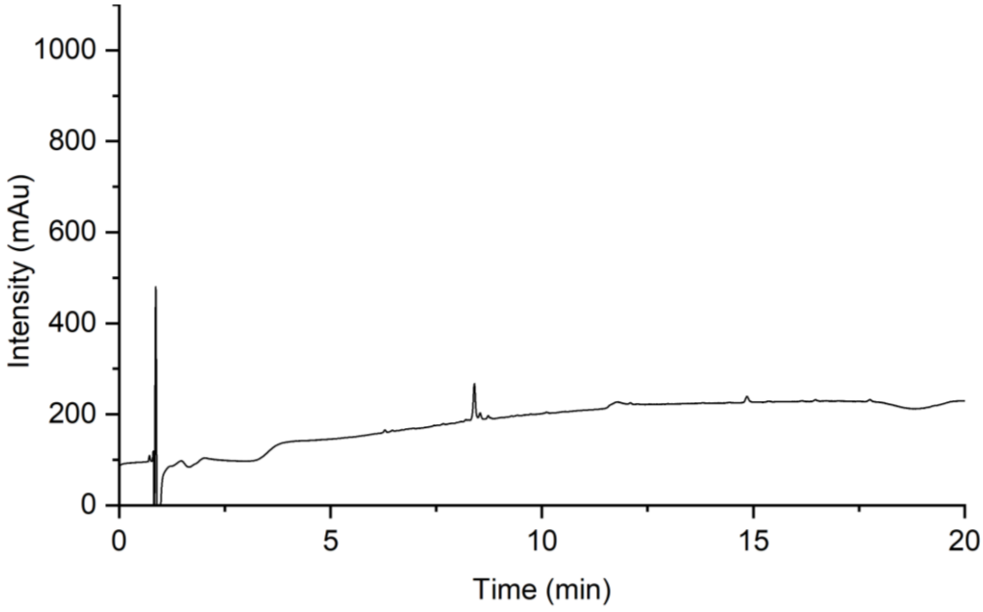

Figure S43 RP-HPLC chromatogram (from 95/5 to 5/95 Vol.% Water/acetonitrile with 0.1 % formic acid in 20 min at 25 °C) of O1(hex) after incubation of resin-bound O1 with 5 Vol.% TFA in water for 2x30 min and subsequent cleavage with hexylamine. The desired hydrolyzed fragment 1B(hex) can be found at t_R = 8.41 min.

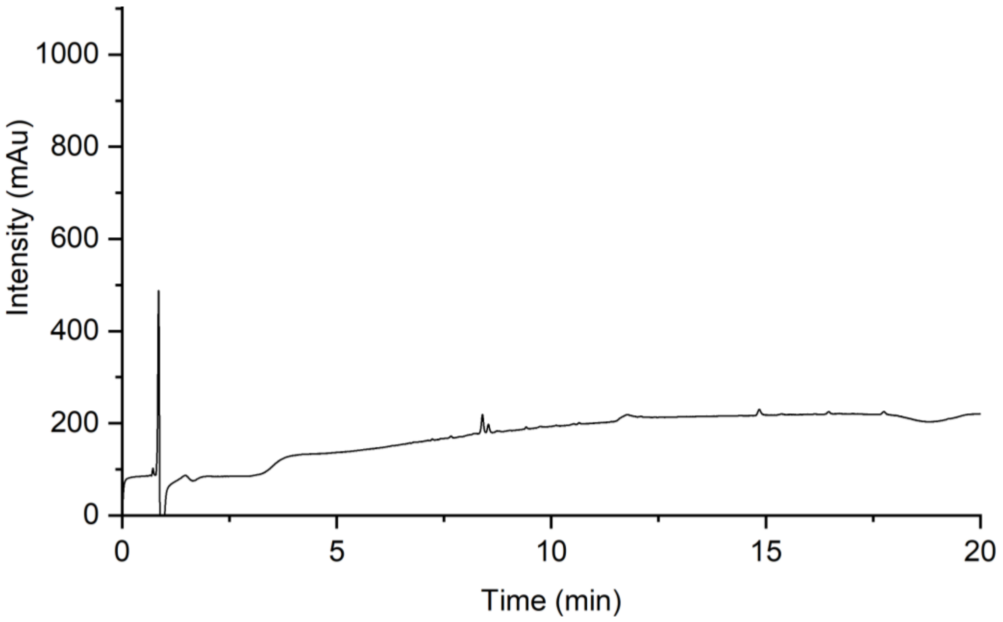

Figure S44 RP-HPLC chromatogram (from 95/5 to 5/95 Vol.% Water/acetonitrile with 0.1 % formic acid in 20 min at 25 °C) of O1(hex) after incubation of resin-bound O1 with 10 Vol.% TFA in water for 30 min and subsequent cleavage with hexylamine. The desired hydrolyzed fragment 1B(hex) can be found at t_R = 8.41 min.

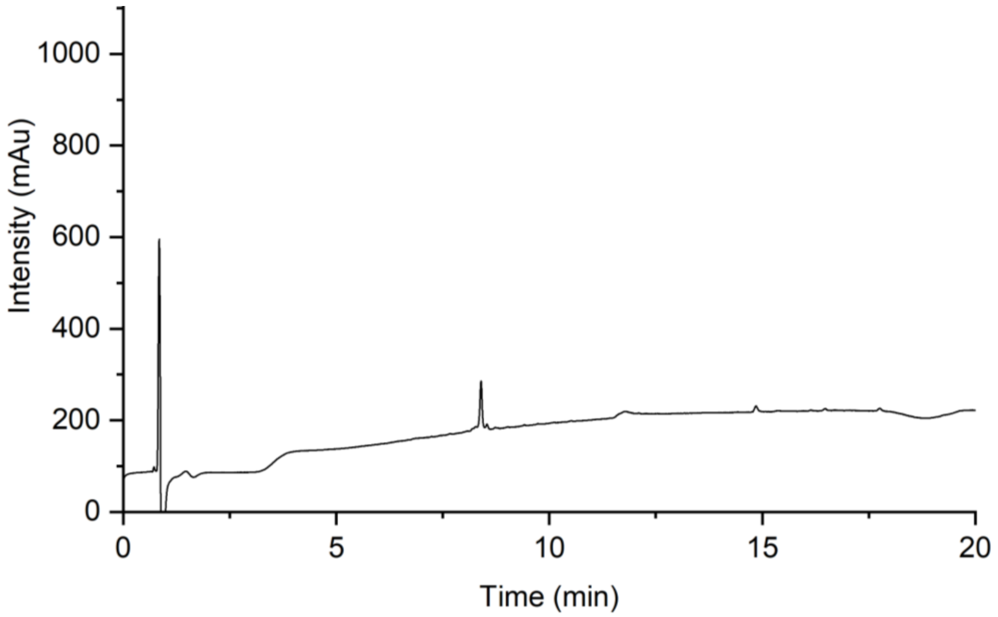

Figure S45 RP-HPLC chromatogram (from 95/5 to 5/95 Vol.% Water/acetonitrile with 0.1 % formic acid in 20 min at 25 °C) of O1(hex) after incubation of resin-bound O1 with 10 Vol.% TFA in water for 2x30 min and subsequent cleavage with hexylamine. The desired hydrolyzed fragment 1B(hex) can be found at t_R = 8.41 min.

e) TMOF activation of resin-bound 1B.

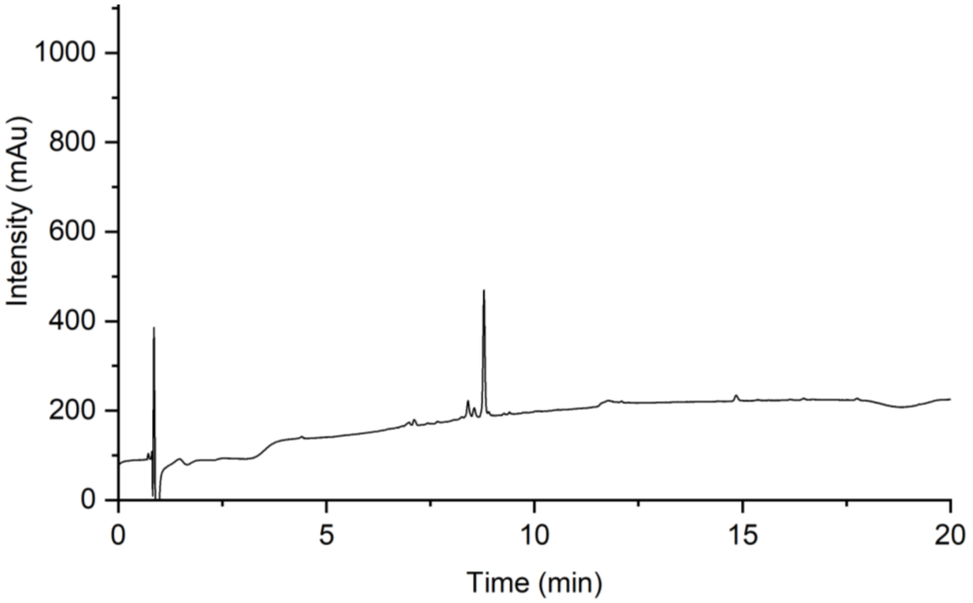

Figure S46 RP-HPLC chromatogram (from 95/5 to 5/95 Vol.% Water/acetonitrile with 0.1 % formic acid in 20 min at 25 °C) of 2 after activation of resin-bound 1B with TMOF and 1 mol% pTSA for 30 min and subsequent cleavage with hexylamine. The main byproduct at t_R = 8.41 min can be attributed to fragment 1B(hex).

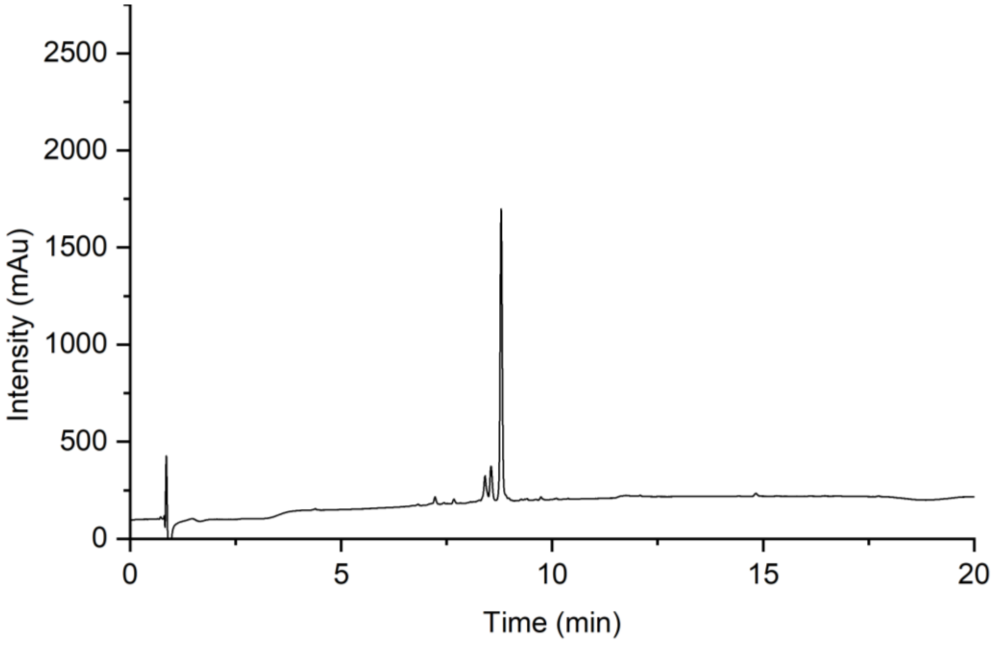

Figure S47 RP-HPLC chromatogram (from 95/5 to 5/95 Vol.% Water/acetonitrile with 0.1 % formic acid in 20 min at 25 °C) of 2 after activation of resin-bound 1B with TMOF and 3 mol% pTSA for 30 min and subsequent cleavage with hexylamine. The main byproduct at t_R = 8.41 min can be attributed to fragment 1B(hex).

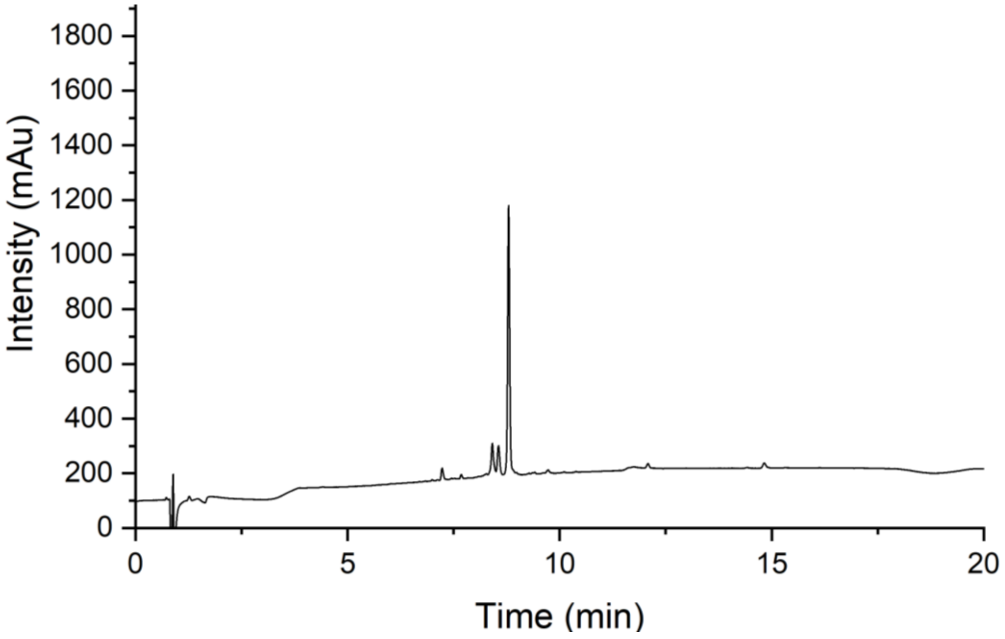

Figure S48 RP-HPLC chromatogram (from 95/5 to 5/95 Vol.% Water/acetonitrile with 0.1 % formic acid in 20 min at 25 °C) of 2 after activation of resin-bound 1B with TMOF and 1 mol% pTSA for 60 min and subsequent cleavage with hexylamine. The main byproduct at t_R = 8.41 min can be attributed to fragment 1B(hex).

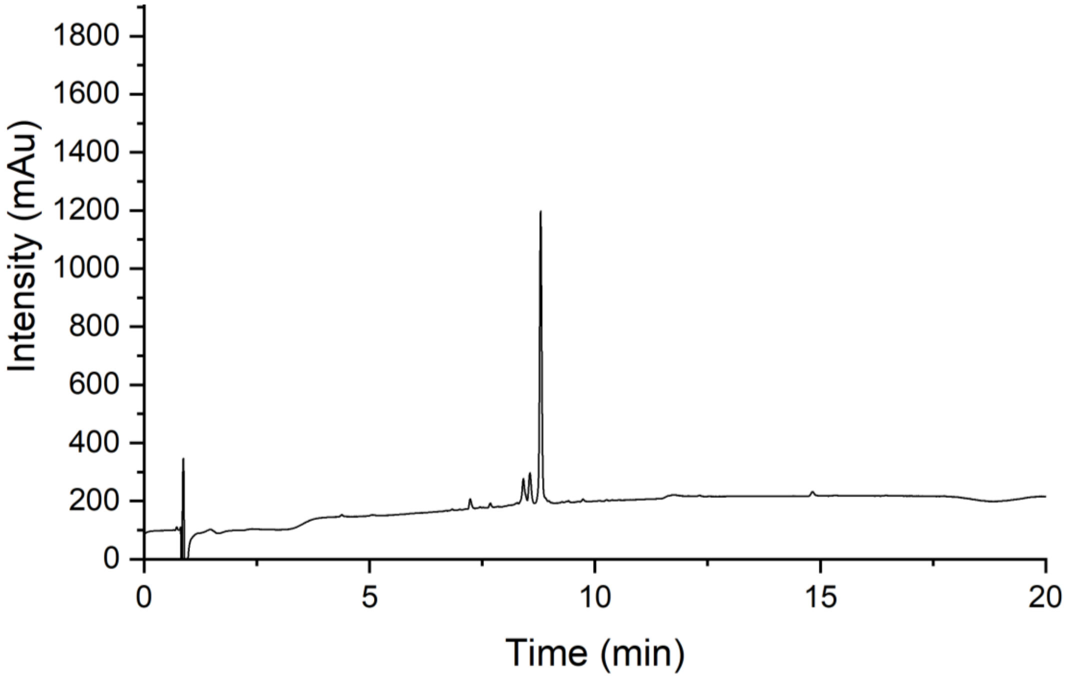

Figure S49 RP-HPLC chromatogram (from 95/5 to 5/95 Vol.% Water/acetonitrile with 0.1 % formic acid in 20 min at 25 °C) of 2 after activation of resin-bound 1B with TMOF and 3 mol% pTSA for 60 min and subsequent cleavage with hexylamine. The main byproduct at t_R = 8.41 min can be attributed to fragment 1B(hex).

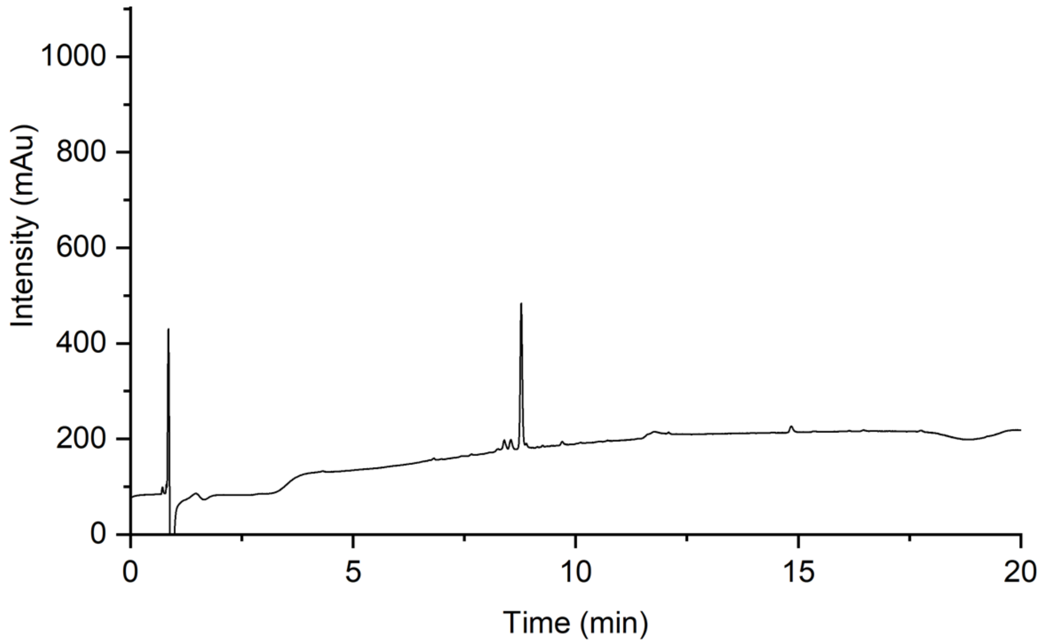

Figure S50 RP-HPLC chromatogram (from 95/5 to 5/95 Vol.% Water/acetonitrile with 0.1 % formic acid in 20 min at 25 °C) of 2 after activation of resin-bound 1Bwith TMOF and 1 mol% pTSA for two 30 min cycles and subsequent cleavage with hexylamine. The main byproduct at t_R = 8.41 min can be attributed to fragment 1B(hex).

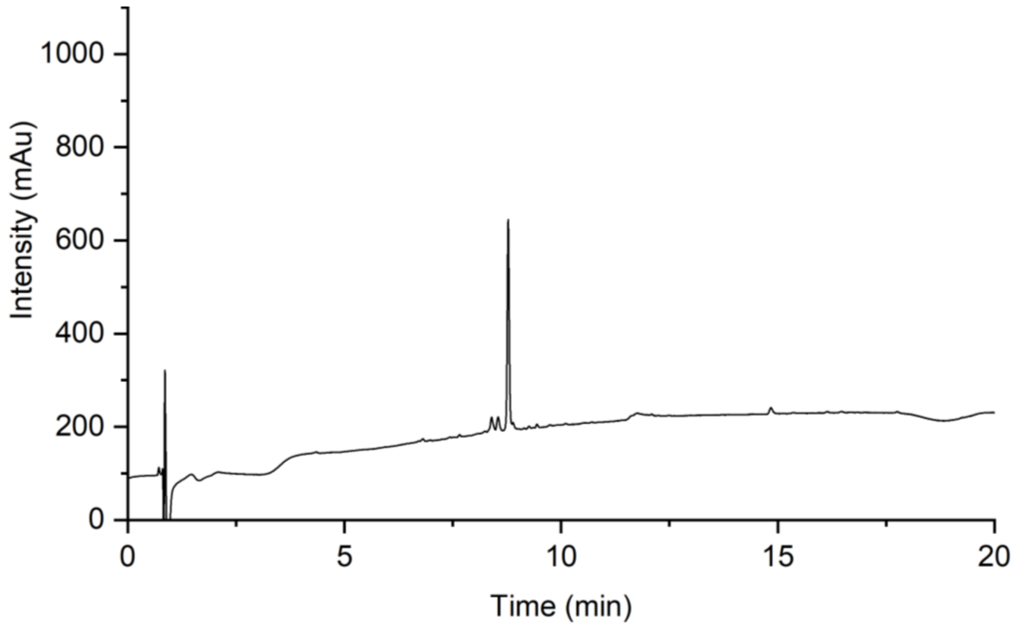

Figure S51 RP-HPLC chromatogram (from 95/5 to 5/95 Vol.% Water/acetonitrile with 0.1 % formic acid in 20 min at 25 °C) of 2 after activation of resin-bound 1B with TMOF and 3 mol% pTSA for two 30 min cycles and subsequent cleavage with hexylamine. The main byproduct at t_R = 8.41 min can be attributed to fragment 1B(hex).

f) *Optimization of on-resin cyclic acetal formation Cyclization*

1) *Variation of reaction time and temperature*

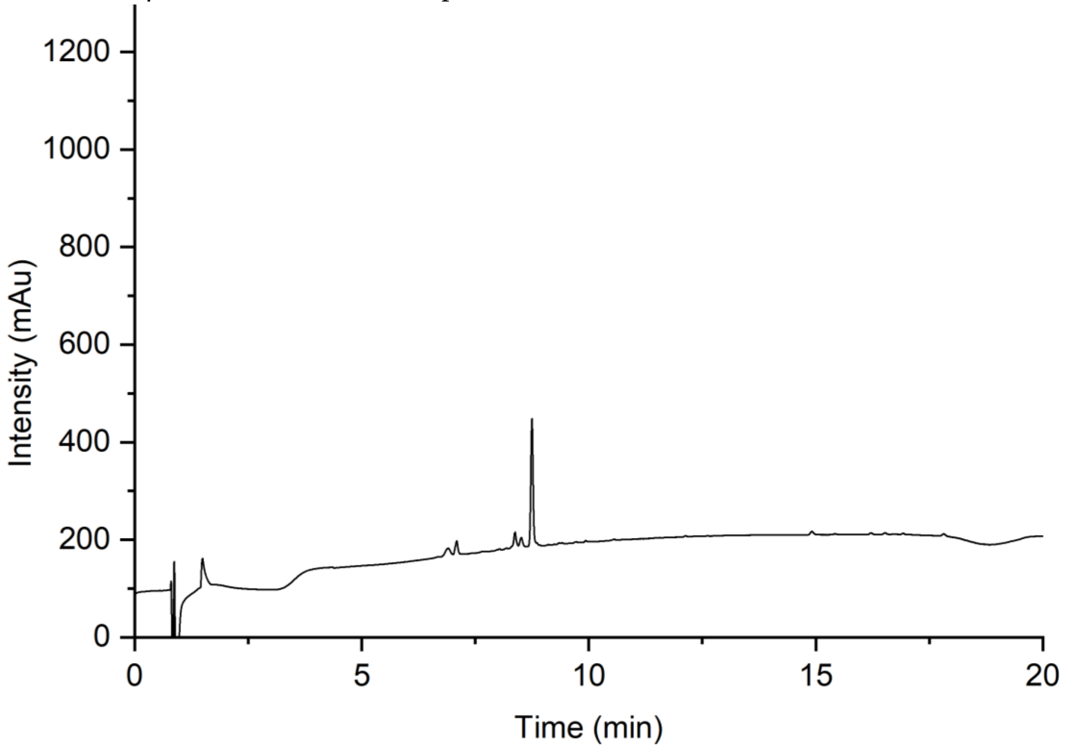

Figure S52 RP-HPLC chromatogram (from 95/5 to 5/95 Vol.% Water/acetonitrile with 0.1 % formic acid in 20 min at 25 °C) after cyclization of 2 with APD – reaction conditions: 12.5 eq. APD, 1 mol% pTSA for **1h** at **37°C** – and subsequent cleavage with hexylamine. Next to the desired product O3(hex) (t_R = 8.41 min) the hydrolyzed aldehyde 1B(hex) at t_R = 8.41 min and the unreacted structure 2 at t_R = 8.80 min can be found in this chromatogram.

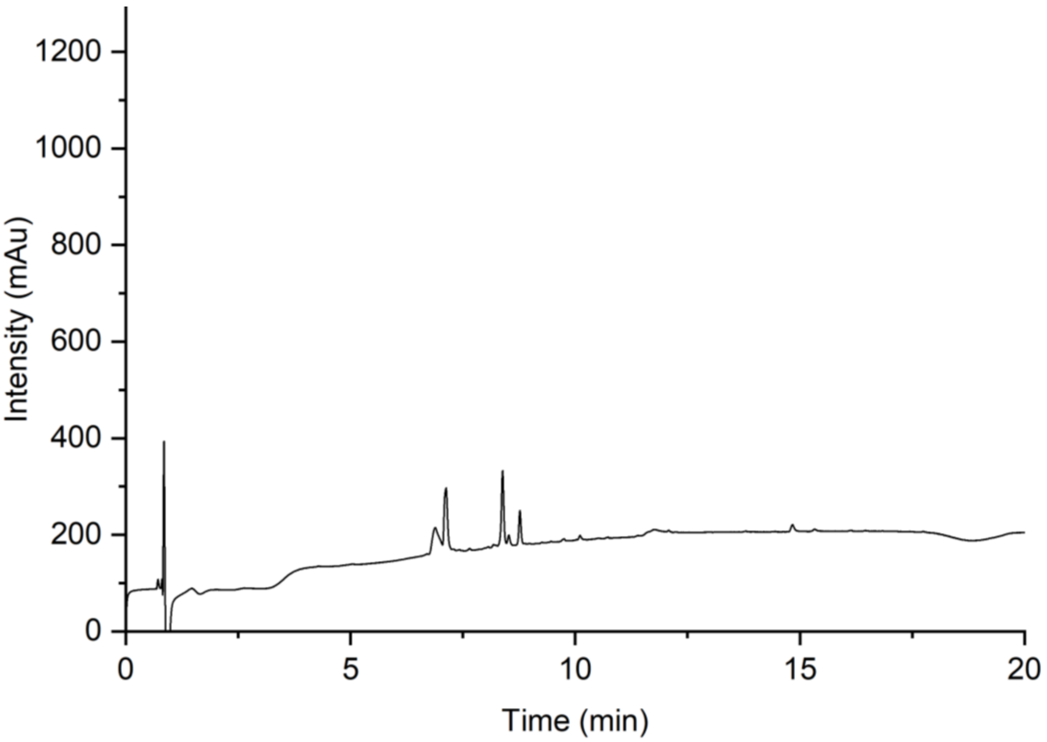

Figure S53 RP-HPLC chromatogram (from 95/5 to 5/95 Vol.% Water/acetonitrile with 0.1 % formic acid in 20 min at 25 °C) after cyclization of 2 with APD – reaction conditions: 12.5 eq. APD, 1 mol% pTSA for **3h** at **37°C** – and subsequent cleavage with hexylamine. Next to the desired product O3(hex) (t_R = 8.41 min) the hydrolyzed aldehyde 1B(hex) at t_R = 8.41 min and the unreacted structure 2 at t_R = 8.80 min can be found in this chromatogram.

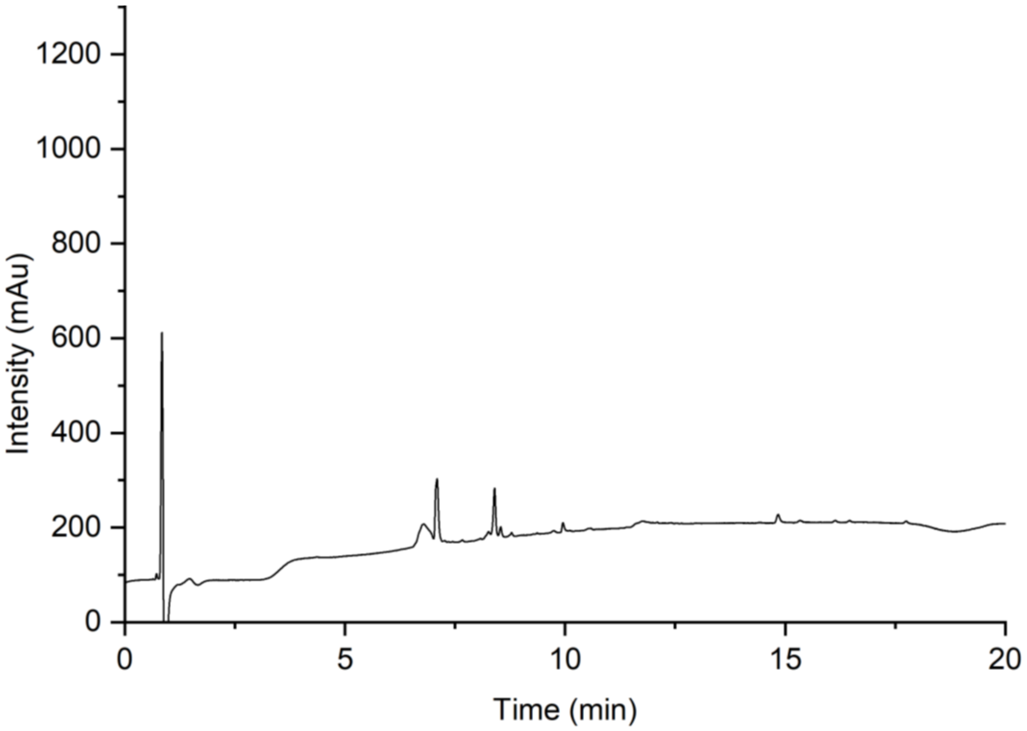

Figure S54 RP-HPLC chromatogram (from 95/5 to 5/95 Vol.% Water/acetonitrile with 0.1 % formic acid in 20 min at 25 °C) after cyclization of 2 with APD – reaction conditions: 12.5 eq. APD, 1 mol% pTSA for **5h** at **37°C** – and

subsequent cleavage with hexylamine. Next to the desired product O3(hex) (t_R = 8.41 min) the hydrolyzed aldehyde 1B(hex) at t_R = 8.41 min can be found in this chromatogram.

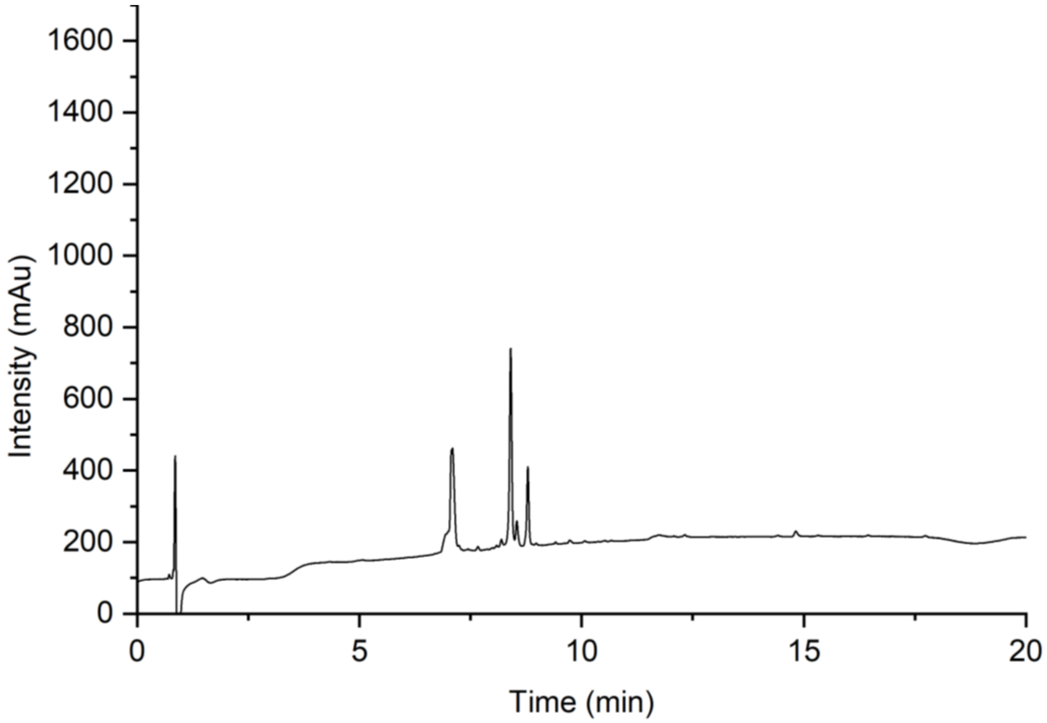

Figure S55 RP-HPLC chromatogram (from 95/5 to 5/95 Vol.% Water/acetonitrile with 0.1 % formic acid in 20 min at 25 °C) after cyclization of 2 with APD – reaction conditions: 12.5 eq. APD, 1 mol% pTSA for **5h** at **RT** – and subsequent cleavage with hexylamine. Next to the desired product O3(hex) (t_R = 8.41 min) the hydrolyzed aldehyde 1B(hex) at t_R = 8.41 min and the unreacted structure 2 at t_R = 8.80 min can be found in this chromatogram.

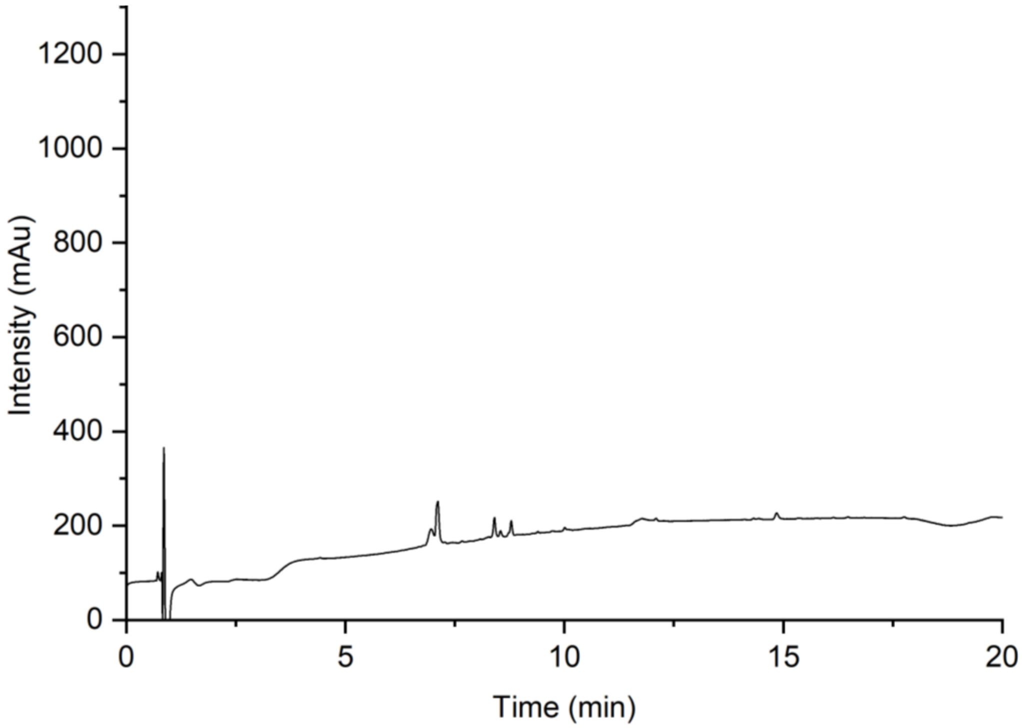

Figure S56 RP-HPLC chromatogram (from 95/5 to 5/95 Vol.% Water/acetonitrile with 0.1 % formic acid in 20 min at 25 °C) after cyclization of 2 with APD – reaction conditions: 12.5 eq. APD, 1 mol% pTSA for **24h** at **RT** – and

subsequent cleavage with hexylamine. Next to the desired product O3(hex) (t_R = 8.41 min) the hydrolyzed aldehyde 1B(hex) at t_R = 8.41 min and the unreacted structure 2 at t_R = 8.80 min can be found in this chromatogram.

2) Catalyst (pTSA)

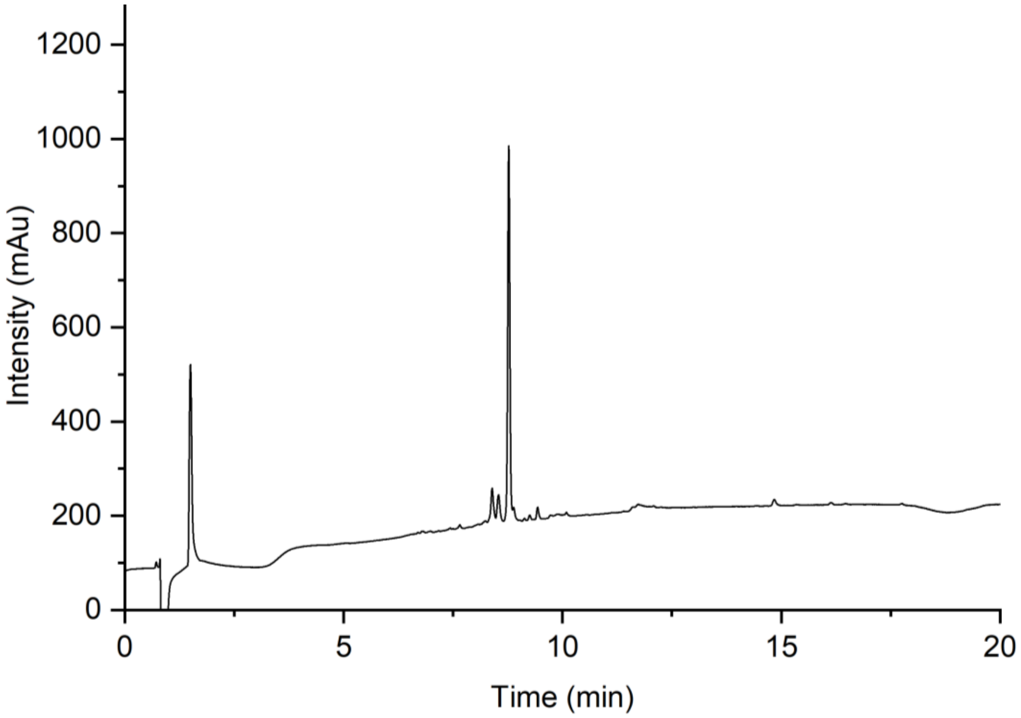

Figure S57 RP-HPLC chromatogram (from 95/5 to 5/95 Vol.% Water/acetonitrile with 0.1 % formic acid in 20 min at 25 °C) after cyclization of 2 with APD – reaction conditions: 12.5 eq. APD, **0 mol% pTSA** for 3h at 37°C – and subsequent cleavage with hexylamine. The desired product can not be found in this chromatogram. The hydrolyzed aldehyde 1B(hex) (t_R = 8.41 min) and the unreacted structure 2 (t_R = 8.80 min) can be found in this chromatogram.

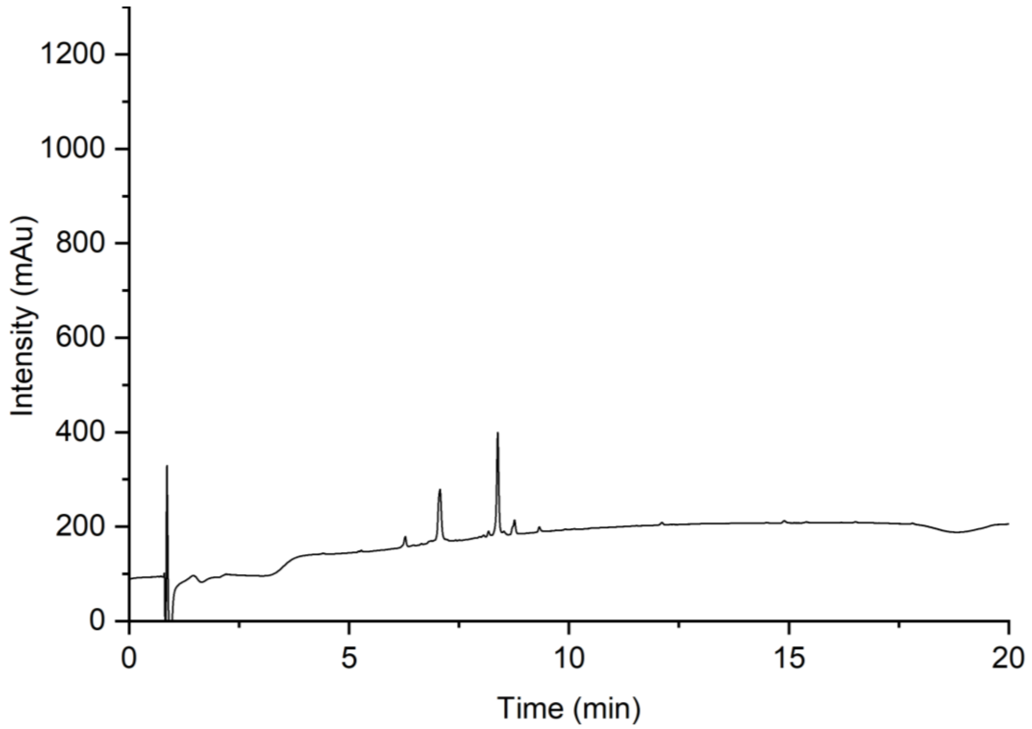

Figure S58 RP-HPLC chromatogram (from 95/5 to 5/95 Vol.% Water/acetonitrile with 0.1 % formic acid in 20 min at 25 °C) after cyclization of 2 with APD – reaction conditions: 12.5 eq. APD, **2 mol% pTSA** for 3h at 37°C – and subsequent cleavage with hexylamine. Next to the desired product O3(hex) (t_R = 8.41 min) the hydrolyzed aldehyde 1B(hex) at t_R = 8.41 min and the unreacted structure 2 at t_R = 8.80 min can be found in this chromatogram.

3) Equivalents of APD

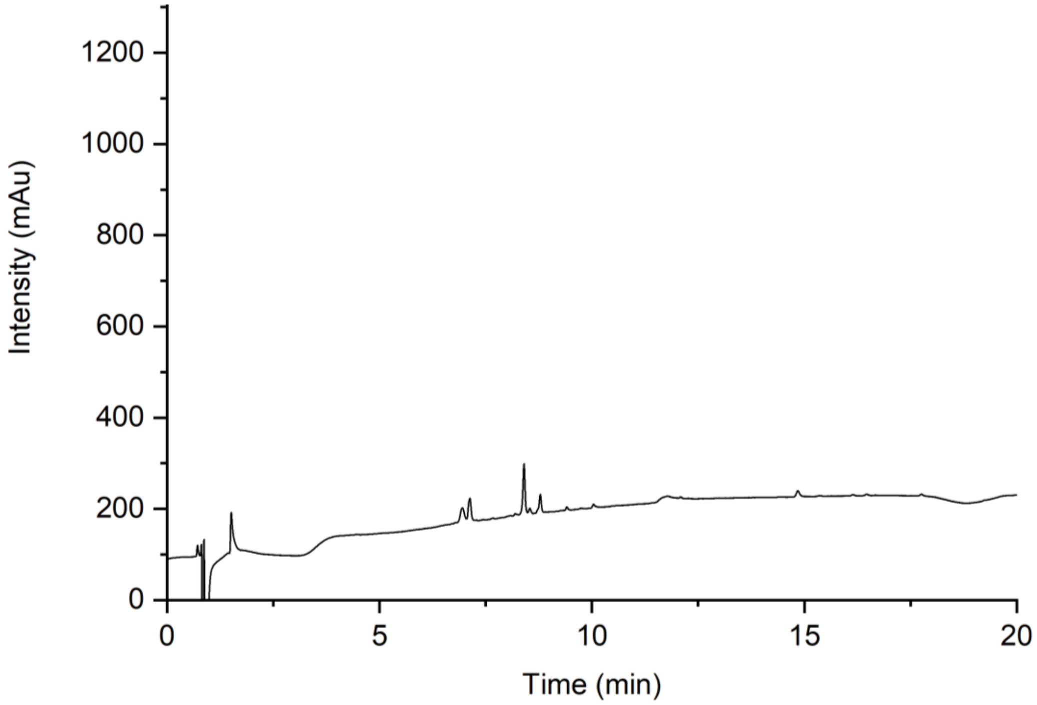

Figure S59 RP-HPLC chromatogram (from 95/5 to 5/95 Vol.% Water/acetonitrile with 0.1 % formic acid in 20 min at 25 °C) after cyclization of 2 with APD – reaction conditions**: 5 eq. APD**, 1 mol% pTSA for 3h at 37°C – and subsequent cleavage with hexylamine. Next to the desired product O3(hex) (t_R = 8.41 min) the hydrolyzed

aldehyde 1B(hex) at t_R = 8.41 min and the unreacted structure 2 at t_R = 8.80 min can be found in this chromatogram.

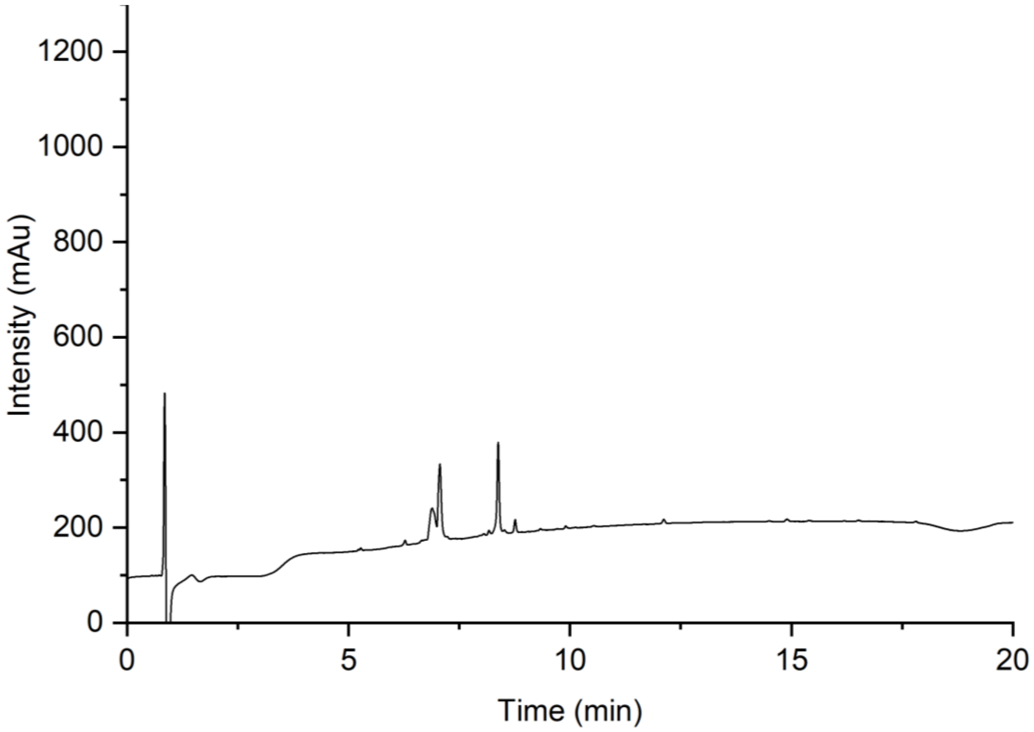

Figure S60 RP-HPLC chromatogram (from 95/5 to 5/95 Vol.% Water/acetonitrile with 0.1 % formic acid in 20 min at 25 °C) after cyclization of 2 with APD – reaction conditions: **25 eq. APD**, 1 mol% pTSA for 3h at 37°C – and subsequent cleavage with hexylamine. Next to the desired product O3(hex) (t_R = 8.41 min) the hydrolyzed aldehyde 1B(hex) at t_R = 8.41 min and the unreacted structure 2 at t_R = 8.80 min can be found in this chromatogram.

4) Combined optimized conditions

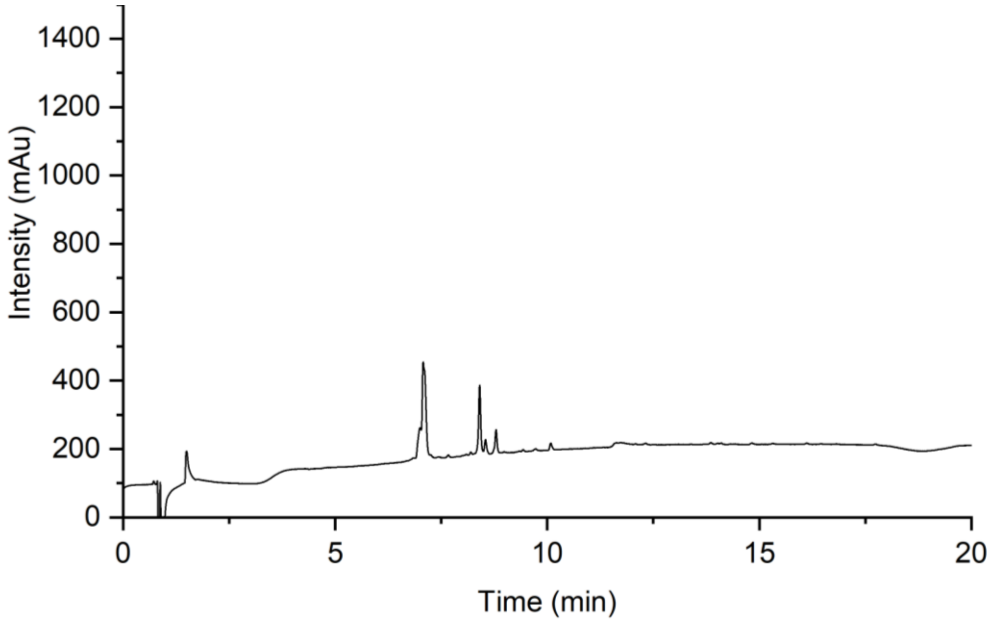

Figure S61 RP-HPLC chromatogram (from 95/5 to 5/95 Vol.% Water/acetonitrile with 0.1 % formic acid in 20 min at 25 °C) after cyclization of 2 with APD – combined optimized reaction conditions: 25 eq. APD, 1 mol% pTSA for 5h at 37°C – and subsequent cleavage with hexylamine. Next to the desired product O3(hex) (t_R = 8.41 min) the hydrolyzed aldehyde 1B(hex) at t_R = 8.41 min and the unreacted structure 2 at t_R = 8.80 min can be found in this chromatogram.

5) Regeneration using recycled APD

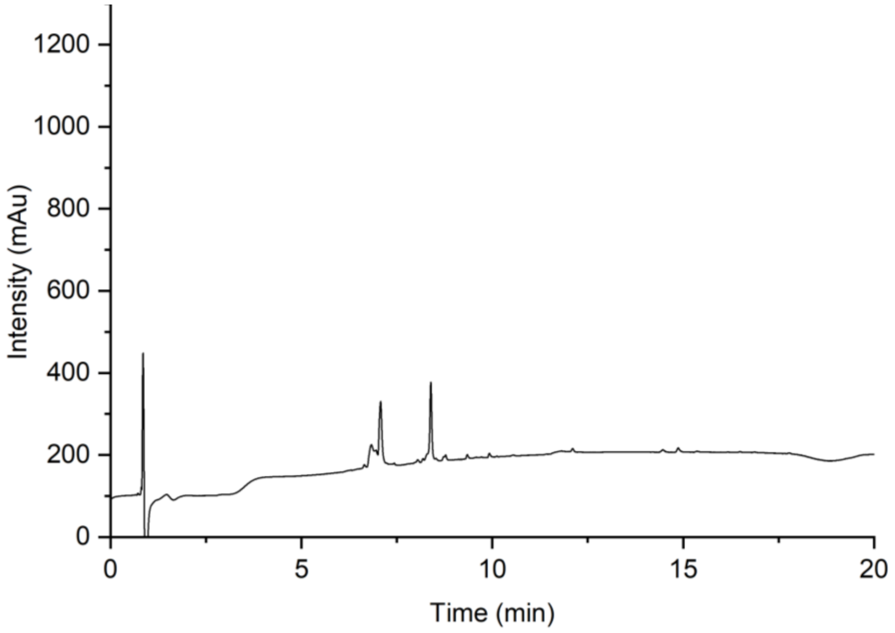

Figure S62 RP-HPLC chromatogram (from 95/5 to 5/95 Vol.% Water/acetonitrile with 0.1 % formic acid in 20 min at 25 °C) after cyclization of 2 with recycled APD (APD_Rec) - Reaction conditions: 12.5 eq. APD_Rec, 1 mol% pTSA for 3h at 37°C – and subsequent cleavage with hexylamine. Next to the desired product O3(hex) (t_R = 8.41 min) the hydrolyzed aldehyde 1B(hex) at t_R = 8.41 min and the unreacted structure 2 at t_R = 8.80 min can be found in this chromatogram.

6) Double cyclization

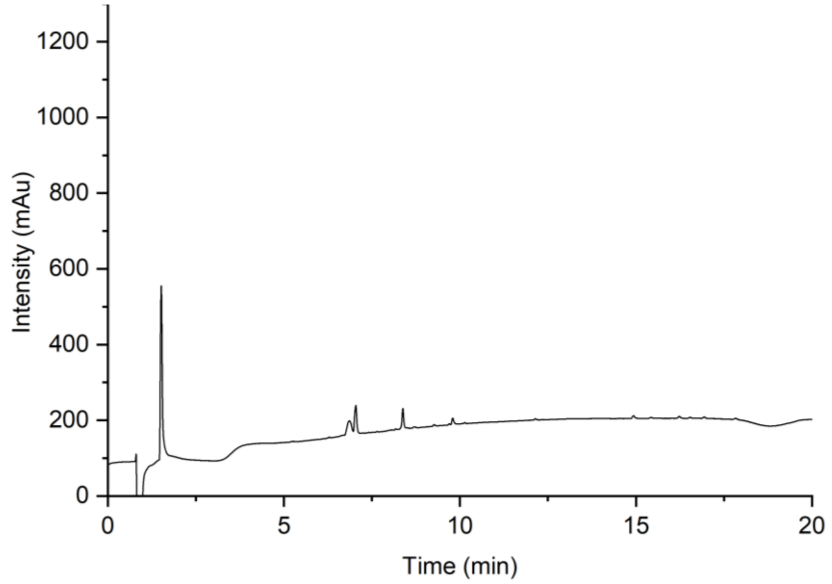

Figure S63 RP-HPLC chromatogram (from 95/5 to 5/95 Vol.% Water/acetonitrile with 0.1 % formic acid in 20 min at 25 °C) after cyclization of 2 with APD – reaction conditions (two cycles with optimal TMOF activation in between): 12.5 eq. APD, 1 mol% pTSA for 3.5h at 37°C – and subsequent cleavage with hexylamine. Next to the desired product O3(hex) (t_R = 8.41 min) the hydrolyzed aldehyde 1B(hex) at t_R = 8.41 min can be found in this chromatogram.

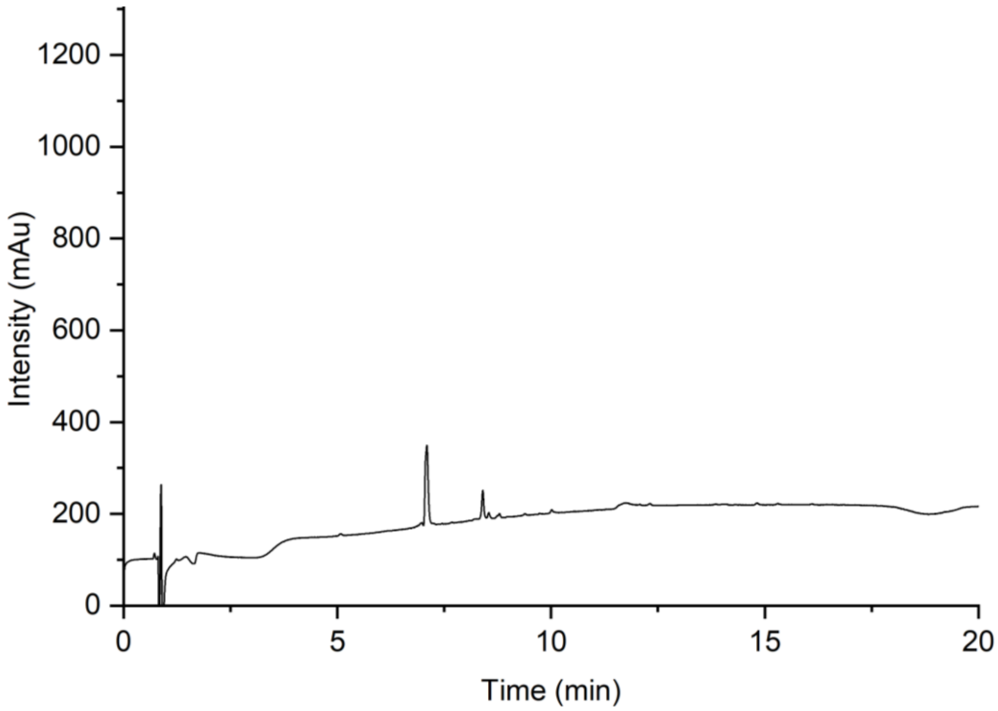

Figure S64 RP-HPLC chromatogram (from 95/5 to 5/95 Vol.% Water/acetonitrile with 0.1 % formic acid in 20 min at 25 °C) after cyclization of 2 with APD – reaction conditions (two cycles with optimal TMOF activation in between): 25 eq. APD, 1 mol% pTSA for 5h at 37°C – and subsequent cleavage with hexylamine. Next to the desired product O3(hex) (t_R = 8.41 min) the hydrolyzed aldehyde 1B(hex) at t_R = 8.41 min and the unreacted structure 2 at t_R = 8.80 min can be found in this chromatogram.

7) Repeatability of DBA regeneration and resin recycling

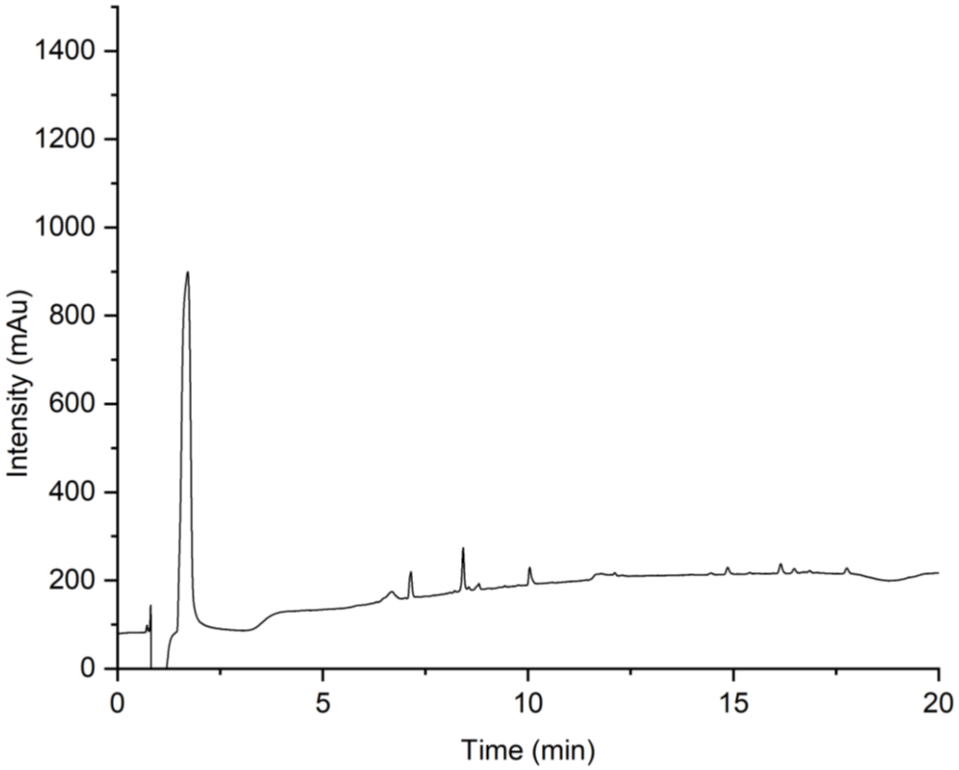

Figure S65 RP-HPLC chromatogram (from 95/5 to 5/95 Vol.% Water/acetonitrile with 0.1 % formic acid in 20 min at 25 °C) after two consecutive cycles of TFA incubation, TMOF activation and cyclization (reaction conditions: 12.5 eq. APD, 1 mol% pTSA for 3h at 37°C) starting from Oligomer O1 – and subsequent cleavage with hexylamine. Next to the desired product O3(hex) (t_R = 8.41 min) the hydrolyzed aldehyde 1B(hex) at t_R = 8.41 min and the unreacted structure 2 at t_R = 8.80 min can be found in this chromatogram.

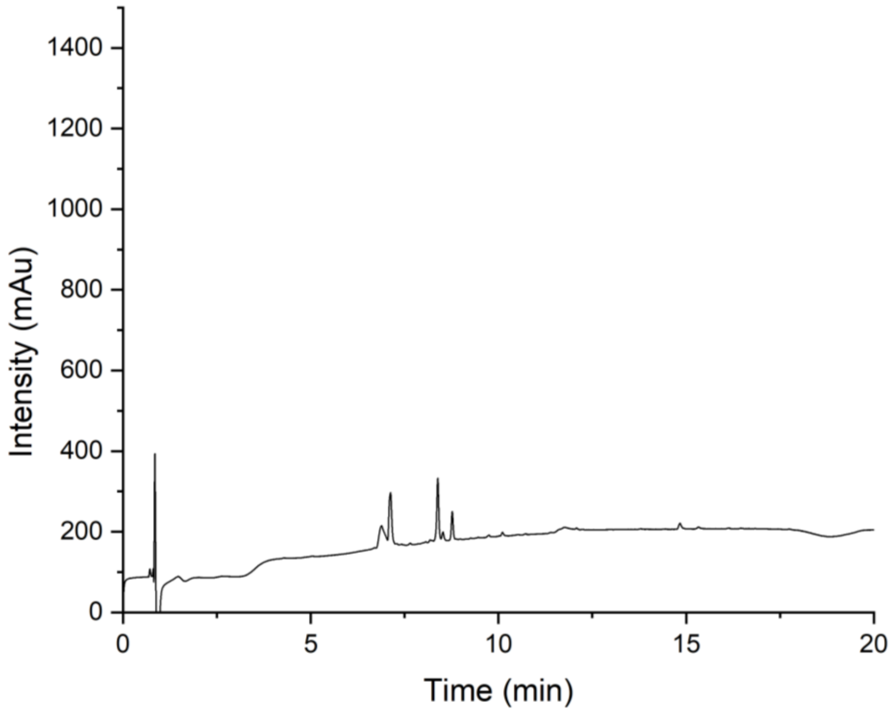

Figure S66 RP-HPLC chromatogram (from 95/5 to 5/95 Vol.% Water/acetonitrile with 0.1 % formic acid in 20 min at 25 °C) after three consecutive cycles of TFA incubation, TMOF activation and cyclization (reaction conditions: 12.5 eq. APD, 1 mol% pTSA for 3h at 37°C) starting from Oligomer O1 – and subsequent cleavage with hexylamine. Next to the desired product O3(hex) (t_R = 8.41 min) the hydrolyzed aldehyde 1B(hex) at t_R = 8.41 min and the unreacted structure 2 at t_R = 8.80 min can be found in this chromatogram.

Supporting Literature

[7] D. Ponader, F. Wojcik, F. Beceren-Braun, J. Dernedde, L. Hartmann, *Biomacromolecules* **2012**, 13, 1845-55.

[20] N. Jäck, A. Hemming, L. Hartmann, *Macromol. Rapid Commun.* **2024**, e2400439.

[23] J. Geng, G. Mantovani, L. Tao, J. Nicolas, G. Chen, R. Wallis, D. A. Mitchell, B. R. G. Johnson, S. D. Evans, D. M. Haddleton, *J. Am. Chem. Soc.* **2007**, 129, 15156-15160.
